# Investigation of the Photoprotective Effects of Various Pigments Against Laser-Marking of Pharmaceutical Tablets

**DOI:** 10.3390/pharmaceutics18060758

**Published:** 2026-06-21

**Authors:** Hadi Shammout, Béla Hopp, Judit Kopniczky, Tamás Smausz, Bence Sipos, Katalin Kristó, János Bohus, Orsolya Jójárt-Laczkovich, Flórián Benkő, Tamás Sovány, Krisztina Ludasi

**Affiliations:** 1 Institute of Pharmaceutical Technology and Regulatory Affairs, University of Szeged, Eötvös u 6, H-6720 Szeged, Hungary; hadishammout1@gmail.com (H.S.); sipos.bence@szte.hu (B.S.); kristo.katalin@szte.hu (K.K.); jojartne.laczkovich.orsolya@szte.hu (O.J.-L.); benko.florian@szte.hu (F.B.); ludasi.krisztina@szte.hu (K.L.); 2 Department of Optics and Quantum Electronics, University of Szeged, Dóm tér 9., H-6720 Szeged, Hungary; b.hopp@physx.u-szeged.hu (B.H.); jkopniczky@titan.physx.u-szeged.hu (J.K.); tomi@physx.u-szeged.hu (T.S.); 3 ELI ALPS, The Extreme Light Infrastructure ERIC, Wolfgang Sandner u. 3., H-6728 Szeged, Hungary; janos.bohus@eli-alps.hu

**Keywords:** nifedipine, film coating, titanium dioxide alternatives, European Union regulations, photostability, laser treatment, pharmaceutical industry

## Abstract

**Background/Objectives**: With the increasing incidence of drug counterfeiting and the emergence of personalized medicine, the need for unique marking of solid dosage forms, e.g., tablets, has attracted considerable interest in the current research and development landscape. Besides traditional printing methods, laser marking offers several advantages, as it eliminates the need for organic solvents and enables the generation of precise patterns. However, laser exposure may raise safety concerns regarding the stability of photosensitive drugs in the irradiated dosage forms. Therefore, the aim of the present study was to test the photoprotective effect of titanium dioxide (TiO_2_) and its various alternatives, e.g., talc, calcium carbonate (CaCO_3_), zinc oxide (ZnO), and black iron oxide (Fe_3_O_4_), alongside a ready-to-use reference formulation, Opadry^®^ Brown, which contains TiO_2_ (titanium-containing, TC) on nifedipine, a light-sensitive model drug. **Methods**: Laser marking or short-term laser ablation at different wavelengths (193 nm, 248 nm, 532 nm, and 781 nm) was applied to different coating formulations. As a positive control, prolonged exposure to daylight was applied. The properties and photostability of these formulations were evaluated using several analytical methods (i.e., surface profilometry, Raman spectroscopy, and high-performance liquid chromatography (HPLC)). **Results**: The TiO_2_, ZnO, Fe_3_O_4_, and Opadry^®^ TC Brown coatings maintained their color during the long-term study under all conditions. Furthermore, the prepared formulations exhibited different ablation depths and morphological changes depending on the coating and laser type. HPLC measurements confirmed significant differences in the protective ability of various pigments against sunlight and different types of lasers. Nevertheless, the obtained Raman spectra were not in complete agreement with HPLC results, which can be attributed to spectral overlap between key nifedipine degradation markers and excipient signals in the tablet core. **Conclusions**: Overall, laser treatment of tablets containing photosensitive drugs may induce API decomposition; however, this effect can be minimized or avoided by careful selection of the appropriate combination of laser type and photoprotective pigment. Under the applied experimental conditions, Ti:Sa laser treatment was associated with the lowest degree of nifedipine degradation among all formulations, while ZnO-containing coatings demonstrated the most consistent photoprotective performance against the majority of the tested laser types, while Fe_3_O_4_-containing coatings provided superior protection during prolonged sunlight exposure and Nd:YAG laser irradiation.

## 1. Introduction

Pharmaceutical film coatings play a crucial role in taste- or odor-masking, in modifying the drug release of certain active pharmaceutical ingredients (APIs), and in improving product stability and shelf life by protecting the core from various environmental parameters such as moisture or light [1,2,3,4,5].

More than 250 APIs are classified as light-sensitive according to the European Pharmacopeia (Ph. Eur.). Photo instability of drugs may result in decreased or complete loss of therapeutic efficacy due to chemical decomposition or the formation of toxic photodegradation products [6].

Titanium dioxide (TiO_2_), talc, calcium sulfate, calcium carbonate (CaCO_3_), aluminum silicate, aluminum hydroxide, or iron oxides are multifunctional, water-insoluble, inorganic components of pharmaceutical products and coating formulations [2,3,4]. Talc is used as a lubricant and adsorbent in tablets and stabilizes emulsions. It is considered the softest mineral (Mohs hardness 1), and its pharmaceutical grade is asbestos-free and meets the USP/EP/JP standards [7,8]. Furthermore, calcium sulfate dihydrate is used in the formulation of tablets and capsules. Its granular form exhibits moderate disintegration capabilities and good compaction properties. This biocompatible excipient comes in a range of rheology, permeability, pore shapes, and particle sizes, and has FDA approval for nutritional applications. The anhydrous modification serves as a desiccant and is hygroscopic [7,9]. Additionally, calcium carbonate (CaCO_3_) is mostly utilized as a filler in SDFs, as a buffering agent, and as a dissolution aid in dispersible tablets, and as a bulking agent in the sugar coating process. It is applied as a base for medicated dental preparations, as a food additive, and therapeutically as an antacid and calcium supplement. Besides that, iron oxides are frequently employed as colorants and UV absorbers in food, medicine, and cosmetics. This group can be found as a powder that is yellow, red, black, or brown. The size, shape, and crystal structure of the particles determine the color [7]. Aluminum hydroxide has low toxicity, high chemical stability, and broad compatibility. It functions as an adjuvant for vaccines, an antacid, and a phosphate binder that controls hyperphosphatemia [7,10]. Moreover, magnesium aluminum silicate is considered a good emulsifier, suspending/thickening agent, adsorbent, and pharmaceutical carrier. Its multifaceted functionalities, inert nature, broad pH tolerance, and compatibility with a wide range of APIs make it an exceptional pharmaceutical excipient [11,12].

As pigments and opacifiers they key function is to enable photoprotection of photoprotective components of film coating formulations, but also serve as fillers that increase the total amount of solids in the coating formulation without significantly affecting the final viscosity of the dispersion [3], may participate in reducing moisture permeability [4], and prevent unwanted mottling [2].

TiO_2_ is the gold standard colorant and opacifier in film coatings [3,13,14,15,16], with the best photoprotection and cover capacity among all white pigments [3,17,18], due to its high refractive index, optimal small particle size, wide bandgap, and high density and stability [14,15]. TiO_2_ effectively improves the stability of coated products at relatively low concentrations [16,19,20], and provides a consistent aesthetic appearance and color for film coatings, improving patient confidence [17,20,21]. TiO_2_ offers several attractive and advantageous characteristics in addition to UV resistance, such as photocatalysis potential and thermal stability. Therefore, it is often used in commercial goods and industrial procedures [22]. In addition, the physicochemical properties of TiO_2_ can vary depending on the primary particle size, crystallinity, and formulation of the material. For example, the scattered light range can be altered by varying the particle size of the powder. Furthermore, TiO_2_ is widely used in confectionery, cosmetics such as sunscreens, and foods, in the plastics industry, and in topical and oral pharmaceutical formulations. It is also present in several veterinary medicinal products, for example, for bees [7]. Moreover, separating out the different functionalities of TiO_2_ for those products in which it serves more than one function is difficult or might not be possible at all [23].

However, the use of TiO_2_ has been associated with some potential health risks [3,13,15,19,21]. Although the European Medicines Agency (EMA) recently (2024) confirmed that pharmaceutical grades of TiO_2_ do not pose a carcinogenic risk in concentrations used in medical products [23], the European Commission (EC) has requested companies to evaluate potential alternatives of this excipient without compromising the safety, quality, efficacy, and availability of medicines [16,18,19,21]. Nevertheless, the Food and Drug Administration (FDA) and regulatory authorities of many other countries allow the further use of TiO_2_ as a food additive, if its content does not exceed 1% by weight [13,24,25].

Therefore, several excipients, including CaCO_3_, calcium hydrogen phosphate, talc, starch, iron oxides, and ZnO [16,23], are intensively evaluated as potential alternatives. Unfortunately, no current alternative covers the full range of applications of TiO_2_, which poses a significant challenge to pharmaceutical scientists. Furthermore, some other issues are associated with possible candidates, such as the risk of API complexation by Ca^2+^ ions in the case of CaCO_3_ [26], or regulatory challenges related to ZnO, which is considered as an API in topical applications [18,27]. Nevertheless, recent studies confirmed that TiO_2_-free (TF) coating formulations demonstrated satisfactory coverage and whiteness that were largely comparable with TC ones, without affecting dissolution [3,28]. Furthermore, the color stability of hypromellose films containing alternative opacifiers such as CaCO_3_ and rice starch was similar to that of TiO_2_, with minor exceptions. This was also found to depend mainly on the type of polymer and the used formulation [24].

Nevertheless, it should be noted that, independently of visual appearance, the photoprotective effect of coating formulations depends on the type of polymer as well as on the type, amount, absorption range, and refractive index of the pigment [18,29,30]. For example, hydroxypropyl methylcellulose (HPMC) exhibits almost complete transparency within the range of 290–450 nm [31], and Béchard et al. found that TiO_2_ and iron oxides have provided the highest contrast for HPMC films and therefore the best photoprotection for nifedipine (≈98%) [32], a well-known photosensitive API, which undergoes photochemical oxidation upon exposure to ultraviolet (UV)/visible (Vis) or daylight, resulting in a decrease in its potency [33]. The process is rapid and not affected by the presence of oxygen [34]. The API primarily decomposes into nitrophenylpyridine and nitrosophenylpyridine through photochemical condensation [18,35]. This confirms the need for intensive testing of various API–pigment combinations, since a wider range of solar radiation reaching the Earth’s surface including the infrared (700 nm–1 mm), the visible (400–700 nm), the ultraviolet A (UVA, 315–400 nm), and a minor fraction of the ultraviolet B (UVB, ~280–315 nm) [36]. Furthermore, it should be noted that although the most energetic ultraviolet C (UVC, 100–280 nm) and the majority of UVB radiation are absorbed by atmospheric ozone and oxygen before reaching ground level, but some of potential laser equipment used to apply individual markings on solid dosage forms by pigment discoloration or surface ablation [37] for anticounterfeiting or personalization purposes [38], works within this range.

Nevertheless, to the best of our knowledge, only a few research studies have been published on the evaluation of the stability of light-sensitive APIs in TF coating formulations, and no studies have evaluated the photoprotective effect against laser-based irradiation. Therefore, the aim of the present study was to evaluate the photoprotective effect of various opacifiers on nifedipine tablets during marking with various lasers available in ELI-ALPS and University of Szeged facilities, such as the following: titanium–sapphire (Ti:Sa) laser (781 nm), neodymium-doped yttrium aluminum garnet (Nd:YAG) laser (532 nm), krypton fluoride (KrF) excimer laser (248 nm) and argon fluoride (ArF) excimer laser (193 nm), against dark-stored samples as negative- and sunlight-exposed tablets as a positive control (PC). The four wavelengths cover both the UV (ArF and KrF excimer), visible (Nd:YAG), and near-infrared (Ti:Sa) regions, thus the full range relevant to pharmaceutical laser marking [12,25]. Nd:YAG and excimer lasers are most widely employed in industrial product marking and coding [30,32], while ultrashort-pulse Ti:Sa systems represent an emerging high-precision alternative for tamper-evident and personalized marking of solid dosage forms [31]. The comparison of these systems therefore reflects realistic processing scenarios.

The chosen wavelengths interrogate distinctly different light–matter interaction regimes: at UV wavelengths several of the studied pigments (e.g., TiO_2_, ZnO, iron oxides) display strong electronic (band-gap) absorption, favoring photochemical/photo-ablative coupling, while at 532 nm absorption becomes highly pigment-selective; and at 781 nm most of the pigments are essentially non-absorbing, so that ablation proceeds through nonlinear (multiphoton/avalanche) mechanisms. This contrast is precisely what allows the photoprotective behavior of the different pigments to be differentiated.

## 2. Materials and Methods

### 2.1. Materials

All materials were used as received from the suppliers. The tablet core consisted of nifedipine (Nifedipine micronized, EGIS Pharmaceuticals, Budapest, Hungary) as API, microcrystalline cellulose MCC (Vivapur^®^ 102, JRS Pharma, Rosenberg, Germany) as filler and dry binder, crospovidone (Kollidon^®^ CL-M, BASF, Ludwigshafen, Germany) as super disintegrant, magnesium (Mg) stearate (Molar Chemicals, Halásztelek, Hungary) as lubricant and anti-adhesion agent, and talc (Molar Chemicals, Halásztelek, Hungary) as glidant at 4%, 87%, 5%, 1%, and 3%, respectively. Nifedipine was chosen as a model drug for this study because of its well-known photosensitivity. Therefore, API samples were carefully handled during the preparation and characterization to avoid further degradation in addition to the applied conditions in the stability studies.

In this study, the aqueous dispersions of film coatings were mainly composed of HPMC (Pharmacoat^®^ 606, Shin-Etsu Chemical Co. Ltd., Tokyo, Japan) as an immediate release polymer, and polyethylene glycol 300 (PEG 300, Fluka AG, Buchs, Switzerland) as a plasticizer. Furthermore, different opacifiers/pigments were added separately to the previous dispersions as follows: Talc, CaCO_3_, ZnO (all from Molar Chemicals, Halásztelek, Hungary) and black iron oxide (Fe_3_O_4_, Sicovit Black 80 E172, Huntsman, Torino, Italy). Dimethicone (Q7-9120 Silicone fluid 20 CST, Dow Corning, Midland, MI, USA) was added as an antifoam agent in the case of the coating dispersion containing CaCO_3_. Although there is no pharmaceutical coating formulation on the market that contains ZnO yet, this excipient was applied here due to its promising potential in this area [18]. Additionally, TiO_2_ (E171, EGIS Pharmaceuticals, Budapest, Hungary) was used as a reference pigment in this investigation. Furthermore, an HPMC-based ready-to-use coating formulation (Opadry^®^ 03F265034 TC Brown, Colorcon, Budapest, Hungary) was also applied as a secondary reference for comparison.

### 2.2. Tabletting by Direct Compression

The nifedipine tablets were prepared by direct compression. After weighing the amounts, the API, filler, and disintegrant were homogenized in a Turbula mixer (Willy A. Bachofen Maschienenfabrik, Muttenz, Switzerland) at 50 rpm for 15 min. The lubricant and glidant were then added and remixed for an additional 2 min. Subsequently, the final mixture was compressed into round biconvex tablets with a mass of 250 mg using a Korsch EK0 single-punch eccentric tablet press (E. Korsch Maschienenfabrik, Berlin, Germany) with a dual tip slightly biconvex punch of 8 mm diameter. The target hardness of the tablets was 90–100 N, which was achieved with 8 mm filling and 3 mm immersion depth.

### 2.3. Film Coating

The prepared tablet cores were coated immediately with HPMC-based aqueous coating dispersions of various opacifiers/pigments (Table 1). Uncoated reference tablets were considered as formulation I, while formulation II was a pigment-free coated reference.

In terms of preparation, the polymer and plasticizer were weighed and gradually added to purified water under continuous stirring using a digital mixer (DLS stirrer, VELP Scientifica, Usmate Velate, Italy). The opacifier/pigment was separately dispersed in purified water using a high shear mixer (Ultra-Turrax^®^ T 25 basic, IKA^®^ Werke, Breisgau, Germany) at 9500 rpm for 30 min. The pigment concentrate was then added to the plasticized polymer solution, and the weight was adjusted to the target with purified water. The mixing speed was set to create a vortex on the dispersion surface that nearly reached the bottom of the mixing vessel (600–650 rpm). After initial mixing, the stirring speed was reduced to 250–300 rpm, and the suspension was deaerated, with a slight vortex to prevent gelation or setting at room temperature. Mixing times ranged from 30 to 120 min, depending on the type of coating. In the case of the CaCO_3_-containing coating (IV), 390 mg of antifoaming agent was added dropwise to the formulation. The Opadry^®^ TC Brown coating dispersion was prepared according to the manufacturer’s recommendations. After preparation, all dispersions were sieved through a 250 µm mesh before the coating process to eliminate aggregates. In addition, gentle stirring was applied throughout the coating process to maintain homogeneity.

Film coatings of batches of 400 g tablets were carried out in a 4M8 Pancoat perforated coating pan (ProCepT, Zele, Belgium) under the specific conditions displayed in Table 2. A 0.5 mm spray nozzle was used, and the atomizing air pressure and airflow rate were 2.0 bar and 0.70 m^3^/min, respectively. The drying and cooling processes lasted 15 min.

A Nikon flagship upright microscope (Nikon Europe B.V., Amstelveen, the Netherlands) was used at a magnification of ×20 to measure the final thickness of the coated tablets. Three tablets from each formula were cut in half along the middle, and each half was measured at a minimum of 10 points (*n* = 10 per half), then the average thickness was calculated [38,39]. Additionally, 20 tablets of each formulation were weighed before and after coating, and the weight gain (*WG*) was calculated using the following formula [40] (Equation (1)):

(1) $$WG\%=\left[\frac{final\;weight-initial\;weight}{initial\;weight}\right]\times 100$$

### 2.4. Storage Conditions and Light Exposure Test

Nifedipine tablet cores and the film-coated tablets were protected from light exposure by storing them in light-resistant glass containers inside cardboard boxes, and these tablets were considered as a negative control (NC) throughout the study. These boxes were kept under ambient conditions (25 °C/40–50% rH) in a dark environment for the desired study period. Nifedipine is not affected by changes in temperature in the absence of light [41] and is the most sensitive at the wavelength of 455 nm [42], but since nifedipine tablets can be exposed to a wider range of wavelengths during various stages of manufacturing, transportation, distribution, and storage, general conditions were selected for photostability studies.

As PC, 10 tablets of each (uncoated and coated) formulation were placed into the window in a single glass container to be exposed to sunlight for 6 months. These samples were daily flipped over to ensure uniform light irradiation from all sides. Three tablets of each were withdrawn after 4, 5 and 6 months, and the API content was determined by HPLC. In addition, the API content of the dark-stored NC samples was also determined. The concentration values were calculated relative to the original amount of API in the tablets.

### 2.5. Laser Treatment of Film-Coated Tablets

QR code marking of nifedipine film-coated tablets was done by a Ti:Sa chirped pulse amplification (CPA) laser (ELI-ALPS Research Institute, Szeged, Hungary), according to the same experimental setup shown in our previous study [38]. Briefly, the laser system provides 92 fs, 4 mJ laser pulses at a 1 kHz repetition rate and a central wavelength of 781 nm. The system consists of an erbium-doped fiber oscillator (C-fiber, Menlo Systems, Martinsried, Germany), an Öffner-stretcher, a regenerative amplifier, a multipass amplifier, and a grating compressor. The beam was moved with the help of a galvo mirror to make the QR code. In the present investigation, a constant laser pulse energy of 280 μJ and a number of pulses (*n* = 100) were applied. The used laser scanning speed was 2.5 mm/s. Furthermore, the laser fluence (F) at the tablet surface can be calculated according to Equation (2).

F = E/πr^2^, (2)

where E is the pulse energy, and r is the radius of the beam on the focal plane. Based on the focused spot diameter of ~100 µm (measured using the knife-edge method at 781 nm), the calculated fluence was around 3.57 J/cm^2^ across the experimental parameter space.

To evaluate the effect of different laser types on the stability and drug content, the same samples were also irradiated with three other types of lasers working at different wavelengths and pulse durations (all of them are nanosecond lasers). These lasers were as follows: a pulsed Lambda Physik COMPEX 205 (Göttingen, Germany) type ArF excimer laser (193 nm), a LLG TWINAMP (Göttingen, Germany) KrF excimer laser (248 nm), and a Quantel Q-smart 450 (Les Ulis, France) Nd:YAG laser (532 nm). The marking speed did not allow the preparation of a complete QR code in these cases; thus, a constant number of holes (*n* = 3) was ablated into the coatings using all three lasers under fixed irradiation conditions (radiation energy: 6.5 mJ, spot size: 0.6 mm, pulse count *n* = 50). Nevertheless, it should be noted that pulse duration, fluence, wavelength, and spot size varied across the tested laser systems, especially if the Ti:Sa laser is compared with the three other systems, which limits direct comparison of their effects during the study.

### 2.6. Surface Profilometry Measurements

For the quantitative analysis of laser ablation depth and microscopic changes in surface roughness, cross-sectional profile readings were taken using contact profilometry (Veeco Dektak 8 Advanced Development Profiler^®^, Veeco, Plainview, NY, USA) along various lines on the ablated areas. The measurement conditions were as follows: tip radius of curvature: ≈2.5 μm, cone angle: 45°, applied force to the surface during scanning: 29.4 μN, scan length: 2000 µm, vertical resolution: 40 Å, and horizontal resolution: 0.1–0.13 μm.

### 2.7. Physical Properties of Uncoated and Coated Tablets

The uncoated and coated tablets (*n* = 20) were randomly weighed using an analytical scale. The breaking force (or hardness ‘H’), diameter, and height of the same tablets were also determined using an MT50 tablet hardness tester (SOTAX AG., Aesch, Switzerland). Additionally, tablet friability (F) was measured by a friability tester (Erweka, Langen, Germany). This test was carried out by placing approximately 6.5 g of tablets from each formula (<650 mg) according to Ph. Eur. in the test drum and rotating 100 times. The weight loss (%) was then calculated.

Furthermore, the residual moisture of the uncoated and coated tablets was investigated with a moisture analyzer (RADWAG MAC 50/NH, Radom, Poland) based on the loss-by-drying method. The duration of the test was around 8 min and the maximum temperature was 105 °C. This test was carried out three times for each formulation (*n* ≈ 10) after storage in dark glass containers.

### 2.8. In Vitro Disintegration Test

The uncoated and coated tablets of nifedipine were tested using a disintegration apparatus (Erweka ZT 71, Erweka GmbH, Langen, Germany) to determine the disintegration time (DT) according to Ph. Eur., 11th Edition (General Chapter 2.9.1 ‘Disintegration of tablets and capsules’). This test was carried out on 6 tablets of each formulation at 37 ± 0.5 °C in 900 mL of distilled water for 15 min.

### 2.9. HPLC Analysis (Photostability Studies)

All tested samples were handled with the exclusion of direct light during the following protocol. Three individually prepared samples from each formulation required for analysis were crushed using a mortar and pestle, dissolved, and diluted with acetonitrile to 25 mL. The samples were then shaken for 2 min to ensure adequate dissolution. Subsequently, they were passed through a microfilter (poly(ether-sulfone) (PES); pore size: 0.45 µm) into light-protected amber glass vials (capacity: 1.5 mL), to prepare the samples for quantification by HPLC.

#### Quantification of Nifedipine

To determine the concentration of nifedipine, HPLC analysis was performed using an Agilent 1260 device (Agilent Technologies, Santa Clara, CA, USA). The stationary phase was a Kinetex^®^ Evo C18 column (5 µm, 150 mm × 4.6 mm, Phenomenex, Torrance, CA, USA). The injection volume was 20 µL. The temperature was set at 30 °C. As mobile phases, purified water (pH 3.0) (A) and acetonitrile (B) were used. Isocratic separation was performed with an A: B ratio of 60:40 for 10 min. The eluent flow rate was 1 mL/min, and chromatogram detection was carried out at 254 ± 4 nm using a UV-Vis diode array detector. Data were evaluated using ChemStation B.04.03 software (Agilent Technologies, Santa Clara, CA, USA). The retention time of nifedipine was detected at 6.209 min. The determined limits of detection (LOD) and quantification (LOQ) were 12 ppm and 37 ppm, respectively.

### 2.10. Raman Microscopy

Raman spectroscopic measurements were performed to detect potential chemical changes in the API structure of both laser-treated and untreated (dark-stored) tablets, with particular focus on spectral markers associated with nifedipine photodegradation. Raman spectra were acquired using a Thermo Fisher DXR dispersive Raman spectrometer (Thermo Fisher Scientific Inc., Waltham, MA, USA), equipped with a CCD detector and a diode laser operating at a wavelength of 780 nm. Measurements were made with a laser power of 12 mW through the 10× microscope objective lens and a slit aperture size of 25 μm.

Spectra were collected for untreated and laser-treated tablets, using an exposure time of 3 s per acquisition. The covered spectral range was 900–1800 cm^−1^, and automated fluorescence correction was applied during the measurements. Data were evaluated with Spectragryph v. 1.2 optical spectroscopy software [43]. Each spectrum represents an average of 6 individual scans. Three samples of each formulation were examined using Raman spectroscopy before and after different laser treatments.

### 2.11. Statistical Analysis

In this research, most of the results were expressed as mean ± standard deviation using Microsoft Excel 2021 software. In addition, statistical analysis was performed with Student’s *t*-test for the comparison of two groups, and one-way ANOVA for more than two groups. Furthermore, the statistical differences in the photostability of samples were analyzed by factorial ANOVA with LDS post hoc comparisons using Statistica v. 14.0.1.25 (Tibco Software Inc, Palo Alto, CA, USA). Differences with *p* < 0.05 were considered statistically significant.

## 3. Results and Discussion

### 3.1. Physical Properties of Uncoated and Coated Tablets (TC and TF)

The tablet cores were yellowish, biconvex, round, without score lines, uniform in size, smooth in texture, and without visible defects. The nominal mass was 250 mg, with a thickness of 3.939 ± 0.02 mm and a diameter of 8.00 ± 0.01 mm. The uniformity of the tablet mass complied with the standards accepted in Ph. Eur. (±5% for tablets ≥250 mg).

Table 3 shows the main physical parameters of the studied formulations. Various formulations required different coating times to achieve complete coverage of the tablets, due to variations in the opacifying ability of the applied pigment. The results are consistent with other studies [38,44].

All formulations showed good resistance to attrition, as no measurable weight loss was detected for any of the formulations [2]. In contrast, significant differences (*p* < 0.05) were observed in the DTs, which correlated well with the coating thickness [15], but all formulations met the USP requirements in the DT of immediate release tablets (within 15 min) [45].

The moisture content of the cores was in good agreement with the acceptable range for nifedipine (≈2.45–2.7%) or even MCC tablets (≤5%) according to the literature [46,47,48,49]. The presence of a polymer barrier around the tablet core further reduced the moisture content and increased the hardness depending on the nature, composition, and thickness of the coating [50,51], enabling suitable stability, as some literature suggests that nifedipine is moisture-sensitive only when stored under unsuitable conditions [52,53]. As it is visible, no significant differences (*p* > 0.05) were found between the moisture content of various formulations, indicating that the type of pigment does not affect moisture absorption, which is consistent with previous studies [15,38].

In terms of mechanical properties, pigment-free coating formulations significantly outperform pigmented ones (*p* < 0.05), which is consistent with the literature, which suggests that the pigment may reduce the mechanical strength of the film, acting as a filler that disrupts the continuous polymer network in the coating [54,55]. Nevertheless, the breaking hardness of the pigmented formulation was in good correlation with the coating thickness, which does not completely support the findings of Khalid et al., who observed that replacing TiO_2_ with other alternatives can negatively affect the mechanical properties of coatings [56]. This discrepancy may be explained by differences in pigment concentration, polymer type, and coating thickness between the two studies, as Khalid et al. employed different formulation conditions, which are known to significantly influence the mechanical performance of pigmented film coatings [47,49]. Gibson et al. also found that the mechanical properties of pigmented HPMC coatings depend on the amount, shape, and size of the pigment particles, as well as the presence of interactive forces between the polymer and the pigment [54]. However, it is not possible to definitively conclude whether the difference between the TC and TF coatings in hardness values will have consequences in the future. These aspects only become apparent during application, when the tablets are packaged, stored, transferred, and exposed to different storage conditions (different temperatures or humidity). It should be noted that the properties of coated tablets after laser treatment have not been evaluated here, as this line has previously been studied in detail [38].

### 3.2. Visual Inspection

**Figure 1:**
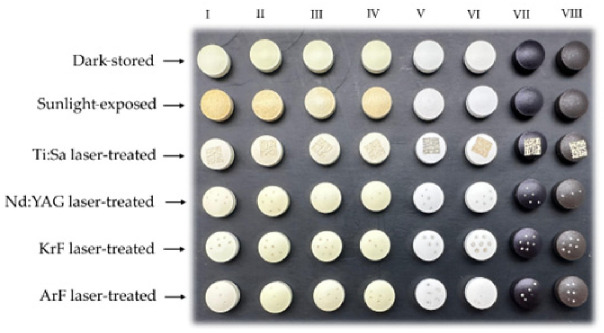
The appearance of the nifedipine formulations (I–VIII) from left to right under different conditions (from top to bottom), respectively.

The detailed appearance of the nifedipine tablets is shown in Figure 1. This picture was taken with an iPhone 14 Pro 9 MP Ultra-Wide Camera (Apple Inc., Cupertino, CA, USA). No color correction was applied, and the tablets were arranged in six rows for easy comparison. The first row represents the uncoated tablets that were stored in the dark. The following rows represent the various film-coated tablets after exposure to sunlight (for 6 months), Ti:Sa laser marking, Nd:YAG laser, KrF laser, and ArF laser ablation, respectively. It should be noted that in the case of the last three lasers, several holes were ablated to the tablet surface instead of a complete QR code, due to the slow marking speed.

No color changes were observed in the case of tablets stored under dark conditions (row 1). However, tablets exposed to sunlight showed considerable color changes, particularly in the first four formulations, possibly due to the photodegradation of the API, which produces colored by-products, such as nitroso derivatives, which range from yellow to brown [57]. This result is consistent with previous findings that HPMC-based coatings did not fully block light on nifedipine tablets, allowing drug degradation that leaches color through the film [58]. In addition, light may also induce degradation of HPMC itself, exacerbated by some pigments that act as photocatalysts [59]. Talc coatings offer poor opacity, while CaCO_3_ coatings provide some cover but still less than TiO_2_, so both pigments permit photodegradation during a long exposure time, especially if the coating is thin (<50–145 µm) [17,29]. Nevertheless, the results of Chuvalo-Abraham et al. confirmed that coatings containing TiO_2_ or even CaCO_3_ maintained their white color under conditions of light and environmental stress [14]. However, it should be noted that the TiO_2_-containing samples (V) exhibited slight browning around the edges of the tablet and a loss of uniform whiteness on the surface. This observation may be due to the different coating thicknesses around the edges or may be explained by the findings of Matsushima et al., who reported that the color of the water-soluble HPMC coatings changed considerably when exposed to sunlight over time. They found that TiO_2_ may be a key participant in this discoloration process. However, these changes were not observed when HPMC was replaced with PVA or Kollicoat IR (immediate release), or when TiO_2_ was replaced with talc, highlighting the importance of the combined effect of these two specific components. Since TiO_2_ is a photocatalyst, it can induce or enhance chemical changes in HPMC when exposed to light. Additionally, although both HPMC and PVA structures are equally susceptible to oxidation, the presence of an oxygen atom adjacent to the secondary carbon atom in the structure of the HPMC polymer facilitates its oxidation more readily. However, the amount of light sensitive amlodipine besylate did not decrease substantially in these tablets [59].

The last three coating formulations demonstrated high stability and no significant color changes until the end of the study. These coatings provided ideal opacity for the nifedipine cores and did not exhibit any other problems related to gloss or color distribution on the tablet surface (row 2). Moreover, all the prepared TF coating formulations retained their original color after the laser treatment under different processing conditions, which is important, as the different pigments may differently absorb the various components of light.

The white color is generally related to the UV protection efficiency of the coating. Talc-based coatings are known to have a lower contrast than TiO_2_-based coatings [3,29], and also have poor or no coverage in the visible or IR range [36]. The opacity and coating whiteness also decrease when TiO_2_ is replaced with CaCO_3_ [29]. This is also consistent with our study, but CaCO_3_ was still ranked the second-best alternative to TiO_2_ after short-term sunlight exposure [18,44], and some literature mentioned that CaCO_3_-based coatings showed the best coverage in a positive correlation with the amount used. On the other hand, a higher amount was required to achieve coverage similar to the reference opacity [44,60]. ZnO coatings showed insufficient opacity in a WG of less than 7%, and did not completely cover the red color of tablet cores [18]. This is in accordance with our results, as a WG of approximately 11% was required to cover the yellow color of the cores in the present study. In contrast to most white pigments, iron oxides exhibit exceptionally high opacity and chemical stability, and can withstand high temperatures, humidity, and light exposure without undergoing significant physical or chemical changes [14,29]. This group covers black, red, or yellow pigments, which could be used in low concentrations for coloring, but limit the achievable color range, and are associated with the dark appearance of tablets [61]. Their use should be encouraged by their global acceptance by regulatory authorities and the distinctive and varied colors they impart to dosage forms to distinguish different strengths and create a brand image [62].

Finally, given that Opadry^®^ TC Brown coatings contain a mixture of pigments (i.e., TiO_2_, Fe_3_O_4_, etc.) [14], their behavior should be between those coatings that contain each of the above pigments individually.

### 3.3. Surface Profilometry Measurements

The determination of the ablation depth may be crucial to fine-tune the laser processing parameters to achieve the desired penetration without causing any damage to the API or the dosage form [63,64]. Table S1 (Supplementary Materials) shows the average values of the ablation depths in the case of Ti:Sa laser-marked tablets in different regions of the QR codes (e.g., center ‘A’, edges ‘B’, and corners ‘C’) for all coating formulations. The values were found to be similar (*p* > 0.05), which indicates the consistency of the coating thickness and its spatial distribution on the surface [65,66].

However, significant differences were observed between these pigmented coating formulations in terms of ablation depths under the same treatment conditions (*p* < 0.05). The order (from highest to lowest) was as follows: VII > VIII > III ≈ IV ≈ V > VI. This can possibly be attributed to differences in optical absorption and scattering properties of the pigments at the near-infrared wavelengths, which could affect the interaction behavior with this laser [39,67]. The interactions of pigments with various lasers differ considerably from those of other components of coating formulations. Pigments that show higher absorption at a specific wavelength undergo greater energy deposition, resulting in deeper ablation, while strong scattering decreases effective penetration and ablation efficiency, resulting in the formation of shallow craters [68]. The intrinsic optical properties of pigments, such as the absorption spectrum and wavelength-dependent light absorption, directly control the efficiency of laser energy absorption and consequently the ablation depth [69]. The mechanism of the laser ablation of the TiO_2_ (reference) has been extensively discussed in the literature [38]. Unfortunately, there is insufficient information on the potential mechanisms of other pigments.

Nevertheless, the narrow band gap of Fe_3_O_4_ allows effective absorption along NIR wavelengths and all visible light [61]. The complex composition of Opadry^®^ coatings and thus the combined absorption wavelength range of the pigments may also justify our results. Other pigments are mostly transparent at this applied wavelength due to the lack of active chromophore groups in this range. Nevertheless, each material has its characteristic ablation threshold, which is specific to the material, to the type of laser, to the ablation method, to the wavelength, and to the fluence. Therefore, the desired ablation depth can be achieved by selecting the appropriate parameters [67]; thus, the ablation was highly efficient because of the high peak intensities of pulsed NIR lasers, which drive nonlinear processes without relying on linear absorption. Moreover, multiphoton absorption dominates, creating seed electrons that trigger avalanche ionization. This leads to plasma formation at the focus, rapid energy dumping, and ablation depths that scale with pulse energy, even in ‘transparent’ media [70]. It should be noted that the mechanistic interpretations of optical absorption, scattering, multiphoton absorption, and plasma formation are based on speculative theoretical considerations and are not definitive conclusions. In this study, no direct optical or spectroscopic characterization was performed; thus, these interpretations require further experimental validation in the future.

It should also be mentioned that the difference in the coating thickness that is required to achieve full coverage for each formulation also seems to contribute to ablation effectiveness, as thicker films increase the optical path length, decrease fluence at the ablation site, and limit depth under fixed conditions. Meanwhile, thinner layers allow a higher energy density to be captured by pigments, which improves photothermal vaporization [16,71]. This can be observed through the corresponding values of the Fe_3_O_4_ and ZnO coatings, which show the lowest and highest thicknesses, respectively. The remaining formulations fall between these two coatings because of their similar thickness. In addition, the Opadry^®^ coating comes in second place because it contains a mixture of pigments (Fe_3_O_4_ and TiO_2_ [14]) and has a lower thickness.

Table S2 shows the ablation depths of coatings after laser treatment at different wavelengths. Depth values appear to be greater with NIR and UV lasers compared to Vis ones [72] due to differences in processing conditions and the nature of the used materials.

However, iron oxides, which are good absorbers in the visible range [36,61], enabled a greater depth of ablation in the visible spectrum, especially along with the thinner coating thickness compared to other formulations. Other studied pigments are almost transparent in this spectral range [29,36,73,74], and consequently, the thickness of the coating also played a considerable role in this case. Formulation VI appears to be the least affected again due to its greater thickness.

On the other hand, the same pigments exhibit varying degrees of absorption in the UV range. For example, ZnO absorbs at wavelengths of 320–400 nm (UVA) [74], while CaCO_3_ absorbs at wavelengths lower than 214 nm [73,75]. Additionally, iron oxides are good absorbers at wavelengths below 400 nm [29,76], while TiO_2_ absorbs at wavelengths of 290–320 nm (UVB) [74]. However, talc has limited absorption in bulk powder form due to the narrow optical band gap it exhibits [77].

Furthermore, shorter UV wavelengths (193 nm) deliver a higher photon energy, enabling direct bond breaking in many inorganic materials, such as oxides, through fast photochemical processes. This results in cleaner ablations with minimal thermal damage, higher efficiency, and less waste compared to 248 nm, which often involves more photothermal effects [78]. Photochemical and photothermal degradation differ fundamentally in the way the absorbed energy drives bond breaking. In photochemical degradation, light directly breaks bonds through electronic excitation (a quantum process), while in photothermal degradation, light first transforms to heat, then heat breaks bonds by a thermal process initiated by light. In addition, the photochemical degradation requires photons with energy ≥ bond dissociation energy (typically UV, ≈290–700 nm) and can be associated with ambient temperature. In contrast, photothermal degradation is associated with a broader spectrum (including IR) and is based more on the efficiency of heat conversion rather than specific electronic transitions, thus temperature is critical in this degradation type [79,80].

**Figure 2:**
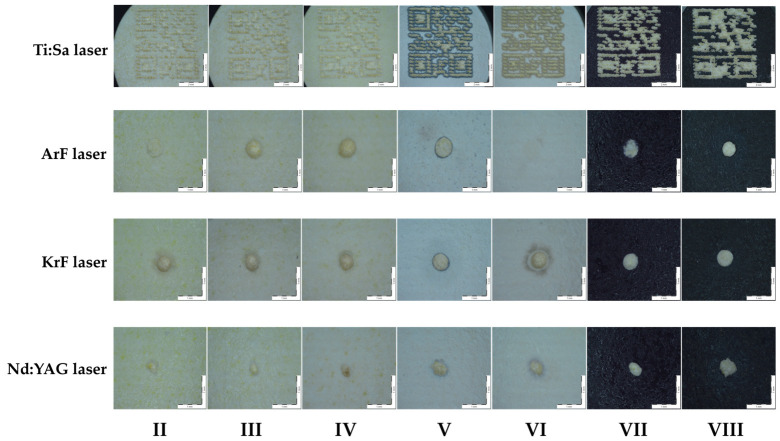
Surface morphology images of all prepared formulations treated by Ti:Sa laser (1st row), ArF laser (2nd row), KrF laser (3rd row) and Nd:YAG laser (4th row) in case of (II) HPMC, (III) Talc, (IV) CaCO_3_, (V) TiO_2_, (VI) ZnO, (VII) Fe_3_O_4_, and (VIII) Opadry^®^ TC Brown coated tablets at 2.5× magnification, from left to right, respectively.

Figure 2 displays images taken from the ablated pattern on the surface of formulations II–VII from left to right, respectively, using a Zeiss stereomicroscope at a magnification of 2.5×. The second row supports the previous hypothesis by showing the absence of thermal degradation in the coating structure. However, it should be noted that the surface of the coating containing TiO_2_ was smoother if treated with the Nd:YAG laser (image V, 4th row) compared to those treated with the UV lasers. This can be explained by the negligible absorption of TiO_2_ at 532 nm, which resulted in selective ablation of the polymer matrix without effective removal of TiO_2_ particles. In contrast, UV lasers operate within the absorption range of TiO_2,_ but even the ArF laser remained below the ablation threshold of TiO_2_, at the applied fluence, and therefore its use is also limited to TF coatings under the current processing conditions [67].

From a microscopic point of view, the ablated areas on the surface of Ti:Sa laser-treated tablets (first row) have a square or rectangular shape. The ablated holes are arranged in a regular, repetitive grid, or matrix pattern. The color of the ablated areas differs from that of the untreated regions. However, in the case of formulation II–IV, the QR code appears faint and discontinuous with poor edge clarity, which can be due to the weak laser-material coupling, indicating low light absorption at this laser wavelength. Localized heating may also contribute to the formation of microbubbles and thus, increased surface roughness. Therefore, these formulations show the lowest potential to be marked with this type of laser due to their light color. However, the contrast becomes higher and more consistent in the case of formulations V and VI. The QR code pattern appears clear, uniform, and well-defined, with sharp, clean edges and little evidence of fusion or irregularity, indicating good absorption and effective ablation. Regarding the formulations VII and VIII, excessive ablation with surface damage is evident. There is a partial loss of structural details. Although the QR code seems to be visible, it is accompanied by a high surface roughness and is somewhat distorted. This indicates high energy absorption and agrees with the averaged values of ablation depths.

Some images of ArF laser-treated formulations (second row) show clean, circular ablated holes with sharp, well-defined borders, while others show irregular edges (II, VI, and VII). This depends on the absorbance of the pigment itself. Pigments with high UV absorbance, such as TiO_2_, cause faster localized heating, larger diameters, and irregular edges. Furthermore, thicker coating layers require more energy to penetrate completely (as in the case of the ZnO coating), resulting in smaller or incomplete crater formation and sometimes leaving a residual layer underneath.

Regarding samples treated with KrF laser (row 3), the thermal contribution has a more pronounced effect due to the lower photon energy. Signs of melting and thermal damage are visible around the craters. The craters also appear smaller, better-defined, and the ablated holes are less deep (especially in the case of formulations VI and VII).

In the case of the Nd:YAG laser (4th row), the photon energy is even lower, so the ablation is based largely on photothermal effects. Since the absorption of most coating polymers is poor in this range, the response will be highly selective for the pigment used. The damage caused by the pigment (burning and melting) can be distinguished. The craters appear smooth, less pronounced, or less precise compared to the previous lasers. The damage is often superficial. The coating may be damaged but not completely removed. The ablated holes are usually irregular. More pronounced thermal halos and color changes can be observed here because of the very localized burning.

Thus, it can be said that the three different lasers also gave different microscopic pictures for the same formulations. These wavelength-dependent differences clearly demonstrate the transition from photochemical ablation at 193 nm to a strong and selective thermal modification of the pigment at 532 nm. Furthermore, no pronounced structural damage was observed after surface ablation, and the coating structure appeared to be intact. This is consistent with our previous studies, where the ArF laser outperformed the continuous wave semiconductor laser (wavelength: 405 nm, spot size: 73 μm, power: 1000 mW, irradiation time: 15–20 ms) and pulsed Nd:YAG laser (wavelength: 1064 nm, power: 1–2.6 W, frequency: 1 kHz). In addition, the semiconductor laser caused black flashes or coating discoloration due to its higher wavelength and greater thermal degradation. Furthermore, the latter type of laser completely burned the same coatings [39,67].

Nevertheless, it should be noted that the measured coating thicknesses were insufficient to protect the tablet core from the effect of laser treatment under the applied conditions. The ablation depths (Tables S1 and S2) clearly indicate that the laser penetrated the coating layer and reached the core. Thus, although laser treatment was primarily intended to mark the coating surface, it is important to consider the possibility that laser-induced photothermal or photochemical effects may penetrate into the tablet core. Nifedipine is well-known for its photosensitivity, and photodegradation forms products such as cis- and trans-azoxy derivatives, nitro derivatives, and lactam derivatives upon exposure to UV or visible light [18,35,57]. Additionally, photothermal effects of laser treatment potentially raise the temperature of the underlying drug core and may also induce thermal degradation or alter the crystalline form of nifedipine [81]. However, the extent of these effects depends on several factors, including the laser wavelength, irradiance, exposure time, and optical properties (absorption and scattering coefficients) of both the coating and the core ingredients [38,72]. Thus, the effect of the laser treatment on the materials in the core needs to be investigated.

### 3.4. Raman Spectra

Raman spectroscopy was employed to assess whether laser irradiation induced localized structural changes in nifedipine within the tablet core, providing spatially resolved chemical information at defined positions relative to the ablation zone, which complements the bulk-averaged quantitative data obtained by HPLC.

**Figure 3:**
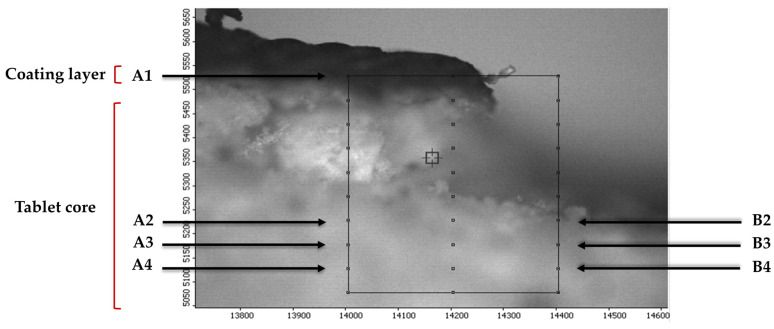
Sampling locations for Raman spectroscopy on the nifedipine halved-tablet surface after the laser treatment.

Spectral data were collected from the halved surface of the tablets, involving both intact and lasered areas. The coating layer and the tablet core are indicated. The following panel (Figure 3) shows the cross-section of a laser-treated tablet with a clearly visible ablation hole on the coating layer. Raman spectra were collected from three positions in the tablet cores. In case of laser-treated tablets, spectra were collected from three points aligned in a row in the intact region of the tablet core adjacent to the ablation zone (A2–A4) and three spectra from points (B2–B4) directly below the lasered area. A1 indicates the intact coating layer. The same procedure was applied for the non-laser-treated tablets, but without B points.

Prior to spectral acquisition, the cross-section of each halved tablet was examined under the optical microscope of the Raman instrument, and measurement points were manually selected to coincide with visually identifiable nifedipine crystals in the tablet core. This targeted approach was necessary because of the low API content (only 4%) of the tablet core, and in the case of random sampling, the spectrum of the heterogeneous matrix would be dominated mostly by signals of the excipients rather than API-specific Raman bands. To minimize the disturbing effect of the surrounding excipients, spectra collected from three different positions were averaged. The evaluation is focused on the region of 900–1800 cm^−1^.

The characteristic peaks of bulk talc powder can be observed at 362 cm^−1^, and 676 cm^−1^ [82], of CaCO_3_ at 282–285 cm^−1^, 712 cm^−1^, and 1085 cm^−1^ [83], while the hexagonal ZnO powder exhibits characteristic peaks around 433–437 cm^−1^ [84], and the peaks of the anatase modification of TiO_2_ can be observed at 398 cm^−1^, 515 cm^−1^, and 640 cm^−1^ [18], all located outside the region evaluated in this study.

**Figure 4:**
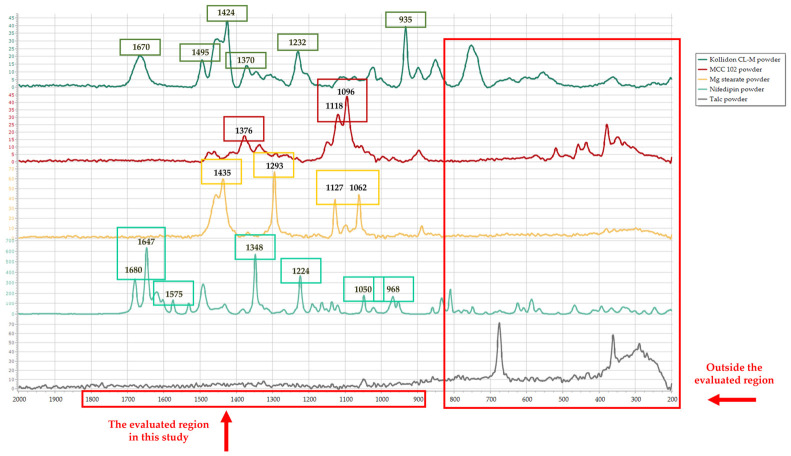
Raman spectra of pure HPMC, Kollidon^®^ CL-M, MCC 102, Mg stearate, nifedipine and talc powders, respectively.

Figure 4 shows the Raman spectra of the nifedipine powder and the excipients in the tablet core. The Raman spectra of the pigments may be found in the Supplementary Materials (Figure S2). The characteristic peaks of nifedipine correspond to the dihydropyridine ring (968 cm^−1^), 1,2-substituted ring (1050 cm^−1^), C-C-O bond in the ester (1224 cm^−1^), NO_2_ symmetric stretching (1350 cm^−1^), NH out-of-plane scissoring vibration (1575 cm^−1^), C=C stretching of the dihydropyridine ring (1647 cm^−1^), and C=O stretching of the ester (1680 cm^−1^).

It should be mentioned that there is no expected interference between the signal of the API and the excipients in the tablet core [85], except the characteristic peak of Kollidon^®^ CL-M at 1670 cm^−1^, which might interfere with the characteristic peaks of the API at 1647 and 1680 cm^−1^. Nevertheless, it is important to note that, according to the literature [81], the degradation of nifedipine can be mainly followed by the change in the intensity ratio of the peaks at 1224 cm^−1^ and 1647 cm^−1^, and by the appearance of a new signal at 1063 cm^−1^ belonging to the breathing of the lactam ring. However, it should be noted that Paraschiv et al. [81] identified these spectral markers in pure nifedipine samples that had been physically separated from excipients prior to measurement, by vacuum evaporation of aqueous solutions. In the present study, Raman spectra were collected directly from the tablet core cross-section in the presence of all formulation components, which fundamentally limits the direct comparability of the two datasets. Unfortunately, this signal shares the position with one characteristic peak of Mg stearate, and together with the interference of the Kollidon^®^ CL-M spectrum, this makes the identification of minor degradation products more difficult if these excipients are present in the studied area.

**Figure 5:**
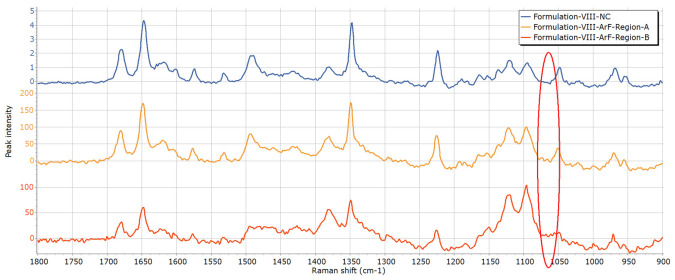
Point-specific averaged Raman spectra recorded directly from nifedipine crystals within the tablet cores for the Opadry^®^ TC Brown coated tablet (formulation VIII) in the case of the dark-stored tablets, and the ArF laser-treated tablets in the regions A and B, respectively, the red circle highlights the area of interest.

Figure 5 displays the Raman spectra of formulation VIII in regions A and B after treatment with the ArF laser as a representative example. In this case, the appearance of the new signal is considerably visible, although its intensity is weak, and so the effect of the Mg stearate cannot be excluded. Raman spectra of other formulations and laser-treated tablets can be seen in the Supplementary Materials (Figures S3–S31). Overall, the Raman spectroscopic approach employed in this study was insufficient to unambiguously confirm or exclude chemical decomposition in the laser-treated tablets. This limitation arises primarily from spectral overlaps between key degradation markers of nifedipine and excipient signals, rather than from an intrinsic insensitivity of the technique itself. These interferences were absent in the reference study by Paraschiv et al. [81], where excipients were removed prior to measurement. Future work should consider the use of confocal Raman mapping or reformulation of the tablet core with spectrally non-interfering excipients to enable unambiguous detection of degradation products in situ.

### 3.5. Photostability Studies and HPLC Measurements

HPLC chromatograms were obtained for extracts of uncoated nifedipine tablets (ground sample) before and after 6 months of exposure to sunlight, as shown in Figure S32 in the Supplementary Materials. In addition, Figure S33 also shows the chromatograms of one of the best formulations (formulation VII, for example) before and after exposure to sunlight, and the different applied laser treatments in this study (Ti:Sa, Nd:YAG, KrF, and ArF laser, respectively) under the same conditions for each treatment. Taken together, these chromatograms illustrate how different wavelengths of light affect the stability of nifedipine, considering the different standards of the applied laser systems.

It should be noted that in the case of the Ti:Sa laser, a full QR code was generated by scanning, whereas the other three lasers were used to ablate a fixed number of holes at discrete positions. This difference introduces not only a variation in total deposited energy, but also in the geometry and temporal distribution of laser exposure across the tablet surface. Consequently, the effectively irradiated volume within the tablet core is not equivalent across the four laser systems, which may also influence the overall decrease in drug content as measured by HPLC, since the proportion of the tablet mass directly exposed to laser irradiation differs between treatments.

The dark-stored samples exhibit a strong main peak around 6 min with an absorbance value exceeding 200 mAU, which may indicate the safety of the nifedipine structure. However, samples subjected to various treatments show approximately the same retention time, confirming that they still have nifedipine, but the peak height decreases to varying degrees depending on the presence or absence of the film, coating thickness, opacity, pigment properties, and different treatment conditions.

Surprisingly, even under dark storage conditions, nifedipine exhibited some degradation or minimal impurities (coated or uncoated samples). A single characteristic peak should be obtained without visible degradation products (a relatively pure baseline). This may explain the increased sensitivity of the API to light over time. It also underscores the need for a much thicker coating on the tablet core (since this formulation shows a relatively small thickness <40 µm), and improved storage conditions to ensure optimal photoprotection. Although known nitroso and nitro derivatives of nifedipine are likely involved, further isolation is required to identify these products. Furthermore, all of these products appear to be more polar than the original compound, which explains their appearance at the beginning of the chromatograms. These findings are consistent with published data [32,57].

#### 3.5.1. Sunlight Exposed Tablets

**Figure 6:**
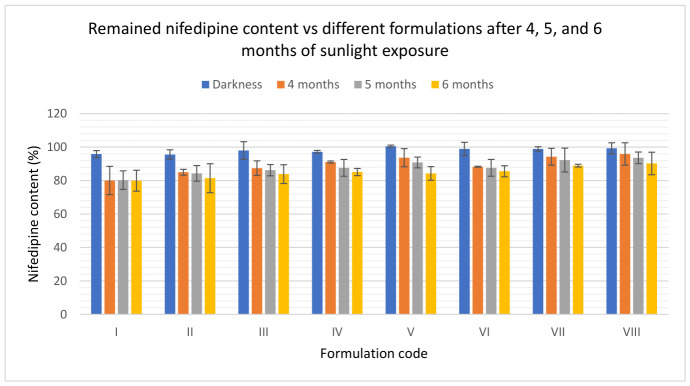
The remaining content of nifedipine (%) for all formulations before and after sunlight exposure under the same conditions. Data are presented as average ± SD (*n* = 3).

Figure 6 displays the results of the photostability tests vs. NC after 4, 5 and 6 months, respectively.

It is well visible that a strong reduction (approx. 20%) was observed in the case of the uncoated tablets (I) after 4 months, but no further decrease was observed until the end of the study, which may indicate that the radiation mostly affected the surface of the tablets, and did not penetrate the deeper layers of the product. The results revealed that the pigment-free HPMC coating (II) provided some protection, but the difference was insignificant (*p* > 0.05) compared to the uncoated tablets. Overall, after 4 months, formulations IV, V, VII, and VIII provided significantly (*p* < 0.05) higher drug content than the uncoated formulation. After 5 months, only tablets coated with formulations V, VII, and VIII showed significantly better drug content compared to uncoated tablets, while after 6 months, only formulations VII and VIII were able to prevent a significant decrease in drug content exposed to sunlight. Although degradation occurred during sunlight exposure, the studied coating formulations retained more than 80% of the API content even after 6 months, indicating relatively good photostability under these conditions. From a pharmaceutical perspective, this suggests that these formulations may maintain acceptable quality and potency during storage or real-world handling with exposure to sunlight, which is critical to ensure consistent therapeutic performance. In addition, this meets the ICH photostability guidelines (ICH Q1B) for pharmaceutical quality [86].

This result is in good accordance with the findings of several previous studies, which described that iron oxides exhibit excellent coverage (compared to TiO_2_ [29]) due to their high refractive index, and degree of protection/contrast ratios by overlapping their absorption spectra with that of the target drug [31,62,76].

Nevertheless it should be noted that the results obtained may also depend on the applied coating thickness, as several studies suggest that the coating thickness should be at least 90–100 μm [32] or even around 134 μm [36], as the TiO_2_ coating formulation failed to protect nifedipine from photodegradation in case of low thickness (≈24–68 μm) [18], which may explain the relatively worse results of formulation V, as it was also reported that the photodegradation rates are higher at lower WG values [62].

The results also confirmed the previous finding that talc and CaCO_3_ do not play the same role as TiO_2_ due to their different chemical structures and refractive index [15,21,24,29]. Galata et al. noted in their study that replacement of TiO_2_ with CaCO_3_ presents a challenge to drug protection against light [15]. However, it should also be noted that our results disagreed with the findings of Radtke et al., who announced that ZnO showed a potential comparable to that of TiO_2_ to protect nifedipine from UV wavelengths (315–400 nm), while all other used pigments (APP117 and APP123) in the same study failed [18].

#### 3.5.2. Laser-Treated Tablets

Before comparing the effects of different laser types on drug stability, it should be acknowledged that the experimental parameters, including pulse duration, wavelength, fluence, and spot size, were not fully equivalent across the tested laser systems, as detailed in Section 2.5.

**Figure 7:**
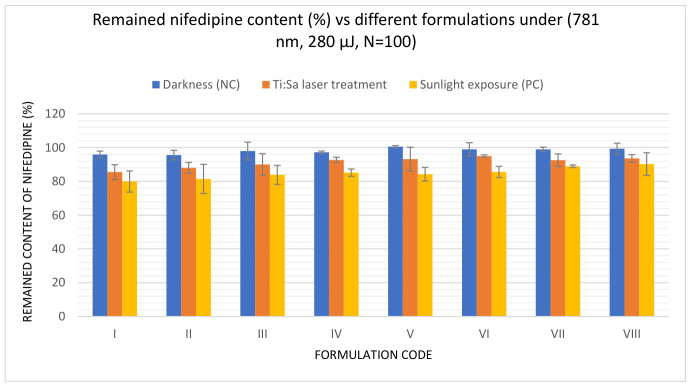
The remaining content of nifedipine (%) for all formulations before and after the Ti:Sa laser treatment under the same conditions. Data are presented as average ± SD (*n* = 3).

**Figure 8:**
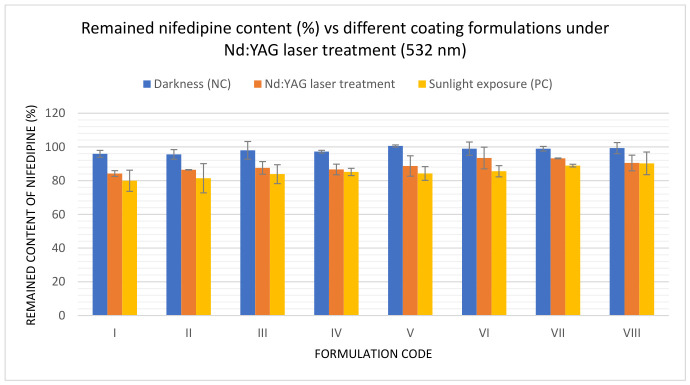
The remaining content of nifedipine (%) for all formulations before and after the Nd:YAG laser treatment under the same conditions. Data are presented as average ± SD (*n* = 3).

**Figure 9:**
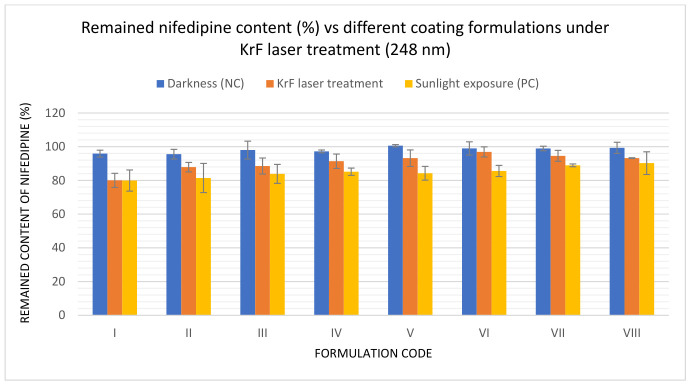
The remaining content of nifedipine (%) for all formulations before and after the KrF laser treatment under the same conditions. Data are presented as average ± SD (*n* = 3).

**Figure 10:**
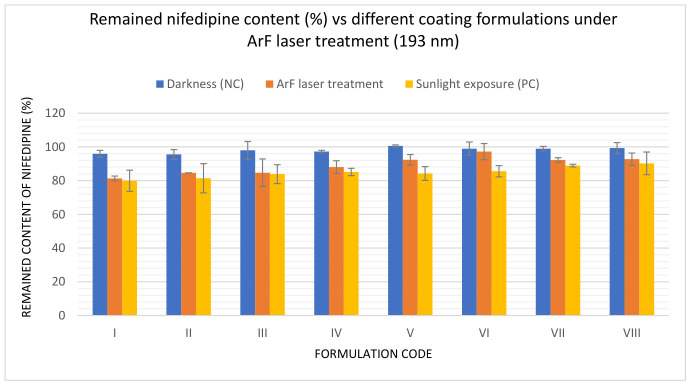
The remaining content of nifedipine (%) for all formulations before and after the ArF laser treatment under the same conditions. Data are presented as average ± SD (*n* = 3).

Figure 7, Figure 8, Figure 9 and Figure 10 display the remaining drug content of the laser-marked tablets in comparison with the dark-stored tablets, as well as the NC and sunlight-exposed tablets after 6 months as a PC. The statistical results (see Supplementary Materials) revealed that the one-time laser treatment of the tablets significantly (*p* < 0.05) decreased the drug content against NC in general, but the results were still significantly better than PC. Furthermore, there was no significant difference between the tested laser types in general, which may be due to the high variability in the response of various coatings to the effects of various laser types.

Nevertheless, the deeper analysis revealed interesting differences between the various pigments and laser types. The lowest decrease in nifedipine content was observed under the applied Ti:Sa laser processing conditions, but the observed differences cannot be attributed exclusively to wavelength-dependent effects. Due to the short exposure time by femtosecond pulses and the relatively long wavelength—as nifedipine is known to have strong absorption in the UV and Vis ranges [87], be the most sensitive between 290–500 nm, and exhibit the highest degradation at 455 nm—the proportions of the decomposition products vary depending on the applied wavelength [18,32,87]. In this case, an insignificant (*p* > 0.05) decrease was observed in drug content compared to NC in the case of formulations IV–VII, which suggests that these pigments were able to absorb the laser energy in a manner that minimally affects the tablet core. This is in good accordance with the fact that most of the studied pigments are essentially transparent (i.e., non-absorbing) at the applied Ti:Sa wavelength of 781 nm (with the exception of Fe_3_O_4_ and Opadry^®^ TC Brown). HPMC itself, like many biocompatible and biodegradable polymers, absorbs in the IR and NIR ranges (≥900 nm) due to its characteristic functional groups [88,89,90], but its absorption appears to be negligible at 781 nm, which is very close to the visible spectrum [91].

In contrast, the wavelength of the Nd:YAG laser (532 nm) falls within the visible range, and it is close to the sensitivity range of nifedipine. Accordingly, this laser type was associated with the most pronounced decrease in drug content among the tested laser systems. Only ZnO (VI) and Fe_3_O_4_ (VII) were able to suitably prevent API degradation. In these cases, the decrease in drug content was insignificant (*p* > 0.05) in comparison with NC. This result may be associated with the higher thickness of the ZnO-containing coating and the dark color of black iron oxide, which offers better energy absorption at this wavelength.

Regarding the UV lasers, the original hypothesis was that the KrF laser, whose wavelength (248 nm) is closer to the sensitivity range of nifedipine, would cause smaller damage than ArF (193 nm), but the results showed the opposite, which may be related to the higher radiation energy of the shorter wavelength laser. Nevertheless, in the case of the KrF laser, formulation V (TiO_2_) and VI (ZnO) were found to provide effective protection, and the drug content after exposure differed insignificantly from NC and was significantly (*p* < 0.05) better than PC. For the ArF laser, only ZnO provided such a level of protection. Nevertheless, it should be noted that the degradation induced by high-energy excimer lasers, particularly the ArF laser (193 nm), is not mechanistically equivalent to conventional solar photodegradation of nifedipine. Conventional photodegradation is a single-photon photochemical process driven by sub-band-gap absorption of UVA/visible photons, promoting oxidation of the dihydropyridine ring to nitroso- and nitrophenylpyridine derivatives over prolonged exposure under low irradiance. In contrast, ArF excimer irradiation at 193 nm delivers high-energy photons (≈6.4 eV per photon) at high peak fluence within nanosecond pulses, sufficient to directly cleave covalent bonds through photochemical/ablative mechanisms, in addition to localized photothermal effects [62,70]. Consequently, although the same nitroso/nitro decomposition products are expected to dominate, since the API chromophore is identical, the energy-deposition pathway, the timescale (nanoseconds versus months), and the spatial confinement differ fundamentally from ambient photodegradation [28]. The laser-induced degradation results should therefore be interpreted as drug instability under high-irradiance ablative conditions, rather than as an accelerated equivalent of conventional photodegradation.

#### 3.5.3. Limitations of the Study

Comparing the results obtained by HPLC and Raman spectroscopy, only partial agreement was observed between the two analytical approaches. While HPLC provided quantitative evidence of nifedipine degradation in several laser-treated formulations, Raman spectroscopy was unable to unambiguously confirm or exclude chemical changes in the API structure in most cases, as discussed in Section 3.4. This discrepancy is not unexpected, given the fundamentally different nature of the two methods: HPLC measures bulk-averaged drug content across the entire ground tablet, whereas Raman spectroscopy samples only a microscopic volume at defined points within the tablet core. Consequently, localized photochemical changes detectable at specific measurement points may not translate into a statistically significant decrease in overall drug content, and vice versa. For these reasons, HPLC remains the more reliable quantitative tool for assessing photodegradation in this system, while Raman spectroscopy, under the current experimental conditions and in the presence of spectrally interfering excipients, served as a complementary but limited method for spatially resolved chemical characterization. Moreover, the Raman sampling strategy and spatial variability could contribute to the interpretation of the observed discrepancies in this context (discussed before).

It should also be noted that the laser systems applied in this study operated under substantially different irradiation conditions, including pulse duration, pulse energy, spot size, irradiation geometry, and fluence. Therefore, the observed differences in nifedipine degradation cannot be attributed solely to wavelength or laser type. In particular, the lower degradation observed after Ti:Sa laser treatment may also be related to the ultrashort femtosecond pulse duration, lower effective thermal load, and different scanning configuration compared to the nanosecond single-spot ablation experiments. Consequently, the present results should be interpreted as comparative observations under practical processing conditions rather than as a direct physicochemical comparison of laser wavelengths.

In addition, a direct optical, spectroscopic or thermal characterization was not performed in this study; thus, many mechanistic interpretations should be considered in future work.

## 4. Conclusions

Although the EU has maintained the use of TiO_2_ in medicines, it has recommended an ongoing search for suitable alternatives. The current study demonstrated the feasibility of replacing TiO_2_ in coatings with different alternatives (talc, CaCO_3_, ZnO and Fe_3_O_4_) to achieve complete coverage and optimal photoprotection of nifedipine tablets from sunlight exposure and from Ti:Sa, Nd:YAG, KrF and ArF laser treatment.

The TC and TF coating formulations exhibited similar properties, and the color stability of the coatings is not affected by the applied treatments, although minor color changes were observed for formulations I–IV after exposure to sunlight, which was probably due to the photodegradation of the drug. On the other hand, the formulations V–VIII maintained their color throughout the study period, and the overall formulations containing TiO_2_ and/or black iron oxide (V, VII, and VIII) were able to suitably protect nifedipine-containing tablets for at least 4 months from direct sunlight exposure. Nevertheless, more specific results were associated with laser treatments. Among the applied experimental conditions, the Ti:Sa laser treatment resulted in the lowest observed nifedipine degradation; it did not affect the appearance of any of the coatings applied, and most of the pigments were able to protect the core from the destructive effect despite the complete ablation of the coating layer. In contrast, the Nd:YAG laser was the most destructive, and only Fe_3_O_4_ exhibited suitable protection against it. The TiO_2_ and ZnO containing coatings exhibited a small colored ring around the ablation region after treatment with UV lasers, which is related to the effective UV absorption of these materials in this wavelength region, which was in good accordance with their good protective effect against these types of lasers.

Nevertheless, this research had several limitations, most notably that the pulse duration, fluence, wavelength, and spot size varied across the tested laser systems, which limits direct comparison of their effects during the study. In addition, HPLC remains the more reliable quantitative tool for assessing photodegradation in this system, while Raman spectroscopy, under the current experimental conditions and in the presence of spectrally interfering excipients, served as a complementary but limited method for spatially resolved chemical characterization. However, future work should consider the application of confocal Raman mapping or reformulation of the tablet core with spectrally noninterfering excipients to enable unambiguous detection of degradation products in situ.

Overall, it can be stated that laser marking of tablets containing photosensitive materials may be done safely, but considerable care must be taken to select the appropriate combination of the applied type of laser and photoprotective pigments.

A future study with matched fluence and irradiation geometry would be necessary for direct comparison of wavelength-specific effects. Furthermore, although direct thermal measurements would strengthen the mechanistic discussion of this research paper, these studies were not considered here, but would also be considered in our future work.

## Figures and Tables

**Table 1:** Components of the coatings.

Coating Composition (%)	I	II	III	IV	V	VI	VII	VIII
**HPMC**		6.5	6.5	6.5	6.5	6.5	6.5	
**PEG 300**		0.65	0.65	0.65	0.65	0.65	0.65	
**Talc**			3					
**CaCO_3_**				5				
**TiO_2_**					9			
**ZnO**						3		
**Fe_3_O_4_**							0.5	
**Opadry^®^ TC Brown**								15

**Table 2:** Coating process parameters.

Step	Inlet Air Temperature (°C)	Exhaust Air Temperature (°C)	Product Temperature (°C)	Drum Speed (rpm)
**Preheating**	60	50	50–55	3
**Coating**	55–60	45	42–45	10
**Drying**	38	33	27	3
**Cooling**	25	25	25	3

**Table 3:** Physical properties of uncoated and coated nifedipine tablets.

Formulation Code	Coating Time (min)	Weight Gain (%)	Coating Thickness (µm)	Moisture Content (%)	Hardness (N *)	DT ** (s)
I ***	-	-	-	2.14 ± 0.16	89.8 ± 3.99	15
**II**	60	4.28	31	1.60 ± 0.24	189.9 ± 6.65	21
**III**	60	4.52	47.33	1.47 ± 0.24	155.9 ± 9.40	26
**IV**	70	5.66	39.33	1.55 ± 0.09	141.2 ± 8.39	24
**V**	60	5.52	38	1.68 ± 0.13	115.3 ± 8.68	80
**VI**	100	11.66	73.33	1.55 ± 0.02	178.9 ± 4.72	110
**VII**	60	3.33	28.33	1.66 ± 0.20	109.4 ± 7.90	28
**VIII**	60	2.04	42	1.70 ± 0.05	112 ± 5.37	100

* N = newton, ** DT = disintegration time, *** Formulation I represents uncoated tablet cores; therefore, coating-related parameters were not determined.

## Data Availability

Data created during the study are displayed within the manuscript and/or in the Supplementary Materials.

## Supplementary Materials

The following supporting information can be downloaded at: https://www.mdpi.com/article/10.3390/pharmaceutics18060758/s1. Figure S1: The measured values of physical properties of nifedipine uncoated and coated tablets (formulations I–VIII): coating time (min), weight gain (%), coating thickness (µm), moisture content (%), hardness (N), and DT (sec), respectively. Table S1: Ablation depth values (µm) for different positions of the QR code region at 280 µJ, and *n* = 100 for each pigmented coating (AV ± SD). Table S2: Ablation depth values (µm) for each pigmented coating under three different laser types and constant conditions (AV ± SD). Figure S2: Raman spectra of pure Fe_3_O_4_, CaCO_3_, Opadry^®^ TC Brown, nifedipine, talc, TiO_2_, and ZnO powders, respectively. Figure S3: Point-specific averaged Raman spectra recorded directly from nifedipine crystals within the tablet cores for the HPMC-coated tablet (formulation II) in the case of darkness, and the ArF laser treatment in the regions A and B, respectively. Figure S4: Point-specific averaged Raman spectra recorded directly from nifedipine crystals within the tablet cores for the HPMC-coated tablet (formulation II) in the case of darkness, and the KrF laser treatment in the regions A and B, respectively. Figure S5: Point-specific averaged Raman spectra recorded directly from nifedipine crystals within the tablet cores for the HPMC-coated tablet (formulation II) in the case of darkness, and the Nd:YAG laser treatment in the regions A and B, respectively. Figure S6: Point-specific averaged Raman spectra recorded directly from nifedipine crystals within the tablet cores for the HPMC-coated tablet in (formulation II) the case of darkness, and the Ti:Sa laser treatment in the regions A and B, respectively. Figure S7: Point-specific averaged Raman spectra recorded directly from nifedipine crystals within the tablet cores for the talc-coated tablet (formulation III) in the case of darkness, and the ArF laser treatment in the regions A and B, respectively. Figure S8: Point-specific averaged Raman spectra recorded directly from nifedipine crystals within the tablet cores for the talc-coated tablet (formulation III) in the case of darkness, and the KrF laser treatment in the regions A and B, respectively. Figure S9: Point-specific averaged Raman spectra recorded directly from nifedipine crystals within the tablet cores for the talc-coated tablet (formulation III) in the case of darkness, and the Nd:YAG laser treatment in the regions A and B, respectively. Figure S10: Point-specific averaged Raman spectra recorded directly from nifedipine crystals within the tablet cores for the talc-coated tablet (formulation III) in the case of darkness, and the Ti:Sa laser treatment in the regions A and B, respectively. Figure S11: Point-specific averaged Raman spectra recorded directly from nifedipine crystals within the tablet cores for the CaCO_3_-coated tablet (formulation IV) in the case of darkness, and the ArF laser treatment in the regions A and B, respectively. Figure S12: Point-specific averaged Raman spectra recorded directly from nifedipine crystals within the tablet cores for the CaCO_3_-coated tablet (formulation IV) in the case of darkness, and the KrF laser treatment in the regions A and B, respectively. Figure S13: Point-specific averaged Raman spectra recorded directly from nifedipine crystals within the tablet cores for the CaCO_3_-coated tablet (formulation IV) in the case of the darkness, and the Nd:YAG laser treatment in the regions A and B, respectively. Figure S14: Point-specific averaged Raman spectra recorded directly from nifedipine crystals within the tablet cores for the CaCO_3_-coated tablet (formulation IV) in the case of darkness, and the Ti:Sa laser treatment in the regions A and B, respectively. Figure S15: Point-specific averaged Raman spectra recorded directly from nifedipine crystals within the tablet cores for the TiO_2_-coated tablet (formulation V) in the case of the darkness, and the ArF laser treatment in the regions A and B, respectively. Figure S16: Point-specific averaged Raman spectra recorded directly from nifedipine crystals within the tablet cores for the TiO_2_-coated tablet (formulation V) in the case of darkness, and the KrF laser treatment in the regions A and B, respectively. Figure S17: Point-specific averaged Raman spectra recorded directly from nifedipine crystals within the tablet cores for the TiO_2_-coated tablet (formulation V) in the case of darkness, and the Nd:YAG laser treatment in the regions A and B, respectively. Figure S18: Point-specific averaged Raman spectra recorded directly from nifedipine crystals within the tablet cores for the TiO_2_-coated tablet (formulation V) in the case of darkness, and the Ti:Sa laser treatment in the regions A and B, respectively. Figure S19: Point-specific averaged Raman spectra recorded directly from nifedipine crystals within the tablet cores for the ZnO-coated tablet (formulation VI) in the case of darkness, and the ArF laser treatment in the regions A and B, respectively. Figure S20: Point-specific averaged Raman spectra recorded directly from nifedipine crystals within the tablet cores for the ZnO-coated tablet (formulation VI) in the case of darkness, and the KrF laser treatment in the regions A and B, respectively. Figure S21: Point-specific averaged Raman spectra recorded directly from nifedipine crystals within the tablet cores for the ZnO-coated tablet (formulation VI) in the case of darkness, and the Nd:YAG laser treatment in the regions A and B, respectively. Figure S22: Point-specific averaged Raman spectra recorded directly from nifedipine crystals within the tablet cores for the ZnO-coated tablet (formulation VI) in the case of darkness, and the Ti:Sa laser treatment in the regions A and B, respectively. Figure S23: Point-specific averaged Raman spectra recorded directly from nifedipine crystals within the tablet cores for the Fe_3_O_4_-coated tablet (formulation VII) in the case of darkness, and the ArF laser treatment in the regions A and B, respectively. Figure S24: Point-specific averaged Raman spectra recorded directly from nifedipine crystals within the tablet cores for the Fe_3_O_4_-coated tablet (formulation VII) in the case of darkness, and the KrF laser treatment in the regions A and B, respectively. Figure S25: Point-specific averaged Raman spectra recorded directly from nifedipine crystals within the tablet cores for the Fe_3_O_4_-coated tablet (formulation VII) in the case of darkness, and the Nd:YAG laser treatment in the regions A and B, respectively. Figure S26: Point-specific averaged Raman spectra recorded directly from nifedipine crystals within the tablet cores for the Fe_3_O_4_-coated tablet (formulation VII) in the case of darkness, and the Ti:Sa laser treatment in the regions A and B, respectively. Figure S27: Point-specific averaged Raman spectra recorded directly from nifedipine crystals within the tablet cores for the Opadry^®^ TC Brown-coated tablet (formulation VIII) in the case of darkness, and the KrF laser treatment in the regions A and B, respectively. Figure S28: Point-specific averaged Raman spectra recorded directly from nifedipine crystals within the tablet cores for the Opadry^®^ TC Brown-coated tablet (formulation VIII) in the case of darkness, and the Nd:YAG laser treatment in the regions A and B, respectively. Figure S29: Point-specific averaged Raman spectra recorded directly from nifedipine crystals within the tablet cores for the Opadry^®^ TC Brown-coated tablet (formulation VIII) in the case of darkness, and the Ti:Sa laser treatment in the regions A and B, respectively. Figure S30: Point-specific averaged Raman spectra recorded directly from nifedipine crystals within the tablet cores in the region (A) for a dark-stored, Ti:Sa laser-treated, Nd:YAG laser-treated, KrF laser-treated, and ArF laser-treated uncoated tablet (formulation I), respectively. Figure S31: Point-specific averaged Raman spectra recorded directly from nifedipine crystals within the tablet cores in the region (B) for a dark-stored, Ti:Sa laser-treated, Nd:YAG laser-treated, KrF laser-treated, and ArF laser-treated uncoated tablet (formulation I), respectively. Figure S32: HPLC chromatograms of nifedipine uncoated tablet (formulation I) before and after sunlight exposure over 6 months under the same studied conditions (HPLC conditions are seen in the text). Figure S33: HPLC chromatograms of formulation VII (optimum coating formulation) before and after prolonged exposure to sunlight over 6 months, and short-term Ti:Sa laser, Nd:YAG laser, KrF laser and ArF laser treatments under the same studied conditions, respectively (HPLC conditions are seen in the text). Table S3: Overall statistical results of sunlight exposure. Table S4: Post hoc comparison of sunlight exposure times. Table S5: Post hoc comparison of various coating formulations against sunlight exposure. Table S6: Post hoc comparison of sunlight exposure results. Table S7: Post hoc comparison of laser treatment methods; Table S8: Post hoc comparison of coating formulations against laser treatment; Table S9: Post hoc comparison of laser treatment results.

## Abbreviations

The following abbreviations are used in this manuscript:

TiO_2_	Titanium Dioxide
CaCO_3_	Calcium Carbonate
ZnO	Zinc Oxide
Fe_3_O_4_	Black Iron Oxide
TC	Titanium Containing
HPLC	High-Performance Liquid Chromatography
EU	European Union
API	Active Pharmaceutical Ingredient
Ph. Eur.	The European Pharmacopeia
EMA	The European Medicines Agency
FDA	Food and Drug Administration
EC	European Commission
TF	Titanium-Free
HPMC	Hydroxypropyl Methylcellulose
UV	Ultraviolet
IR	Infrared
Ti:Sa	Titanium–Sapphire
Nd:YAG	Neodymium-Doped Yttrium Aluminum Garnet
KrF	Krypton Fluoride
ArF	Argon Fluoride
MCC	Microcrystalline Cellulose
Mg	Magnesium
PEG	Polyethylene Glycol
WG	Weight Gain
NC	Negative Control
PC	Positive Control
QR	Quick Response
CPA	Chirped Pulse Amplification
n	Number of Pulses
F	Fluence
E	Energy
r	Radius
H	Hardness
F	Friability
DT	Disintegration Time
Vis	Visible
LOD	Limit of Detection
LOQ	Limit of Quantification
USP	The United States Pharmacopeia
PVA	Polyvinyl Alcohol
NIR	Near-Infrared
