# Supplementary material for: Investigation of the Photoprotective Effects of Various Pigments Against Laser-Marking of Pharmaceutical Tablets
Source: Pharmaceutics. 2026 Jun 21;18(6):758. doi: 10.3390/pharmaceutics18060758 (PMC13306581; doi:10.3390/pharmaceutics18060758)
Supplement: Supplementary file 1 [file pharmaceutics-18-00758-s001.zip › pharmaceutics-4356889-supplementary.pdf]

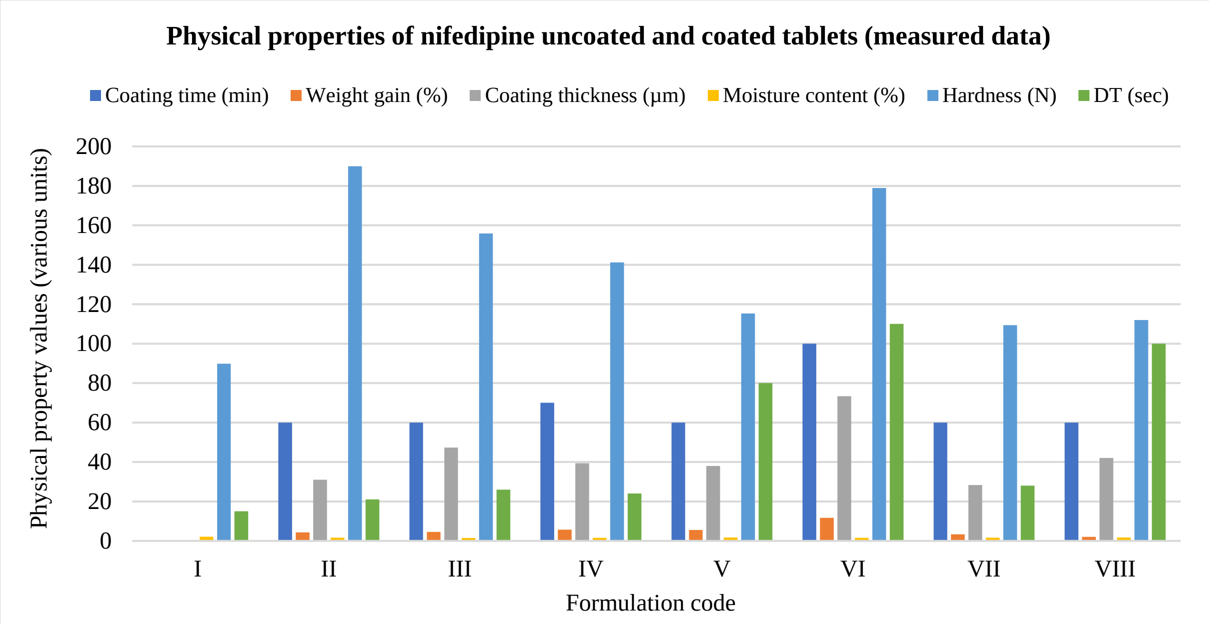

Figure S1. The measured values of physical properties of nifedipine uncoated and coated tablets (formulations I-VIII); coating time (min), weight gain (%), coating thickness (μm), moisture content (%), hardness (N), and DT (sec) respectively.

Table S1. Ablation depth values (μm) for different positions of the QR code region at 280 μJ, and N=100 for each pigmented coating (AV ± SD).

Formulation code	Ablation depths (μm)		
	A (center)	B (edge)	C (corner)
III	133.30 ± 7.62	130.63 ± 8.84	137.59 ± 8.31
IV	138.88 ± 9.60	134.55 ± 9.60	136.85 ± 8.89
V	137.51 ± 8.58	130.72 ± 3.10	131.05 ± 9.69
VI	129.01 ± 3.49	123.09 ± 9.77	126.27 ± 8.12
VII	176.75 ± 9.30	180.25 ± 7.02	176.14 ± 7.50
VIII	174.82 ± 8.07	169.29 ± 7.76	170.83 ± 5.28

Table S2. Ablation depth values (μm) for each pigmented coating under 3 different laser types and constant conditions (AV ± SD).

Formulation code	Ablation depths (μm) at different laser wavelengths		
	ArF laser (193 nm)	KrF laser (248 nm)	Nd:YAG laser (532 nm)
III	121.67 ± 2.20	114.99 ± 3.67	73.37 ± 4.84
IV	124.51 ± 3.23	115.87 ± 4.38	87.89 ± 2.66
V	66.74 ± 2.33	61.20 ± 2.42	67.84 ± 2.88
VI	49.32 ± 3.30	33.20 ± 5.26	25.63 ± 1.92
VII	80.22 ± 3.05	70.92 ± 2.91	111.80 ± 4.22
VIII	75.96 ± 2.87	74.21 ± 3.10	104.22 ± 2.00

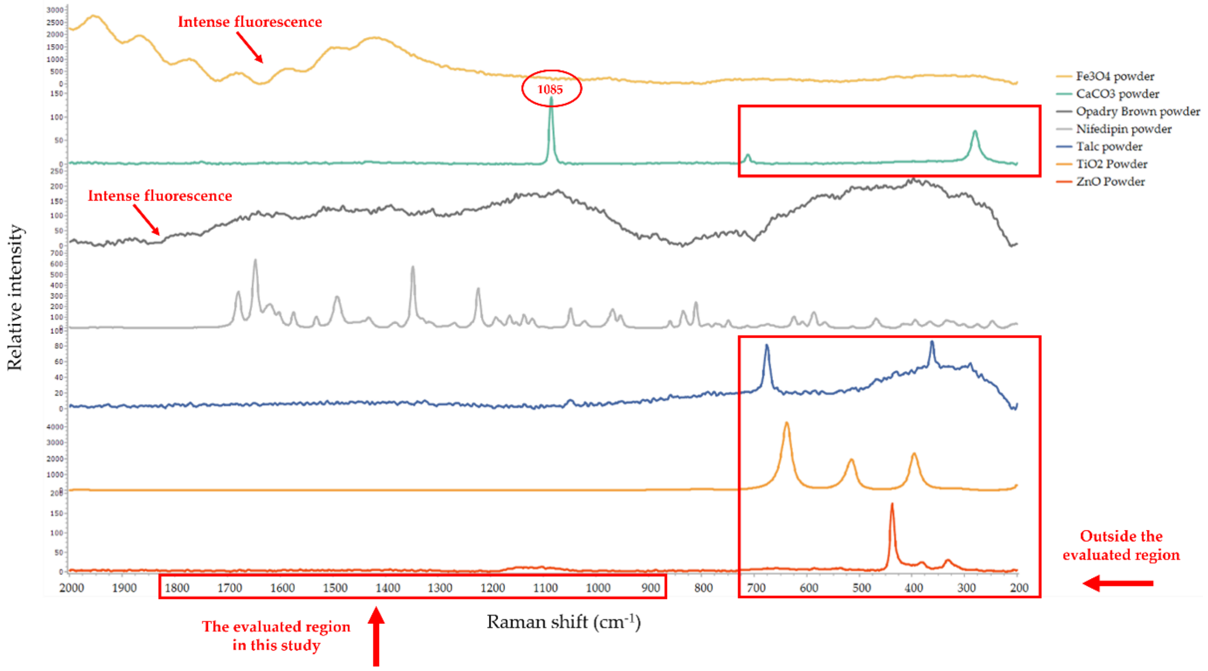

Figure S2. Raman spectra of pure Fe_3O_4, $CaCO_3$, Opadry® TC Brown, nifedipine, talc, TiO_2, and ZnO powders respectively.

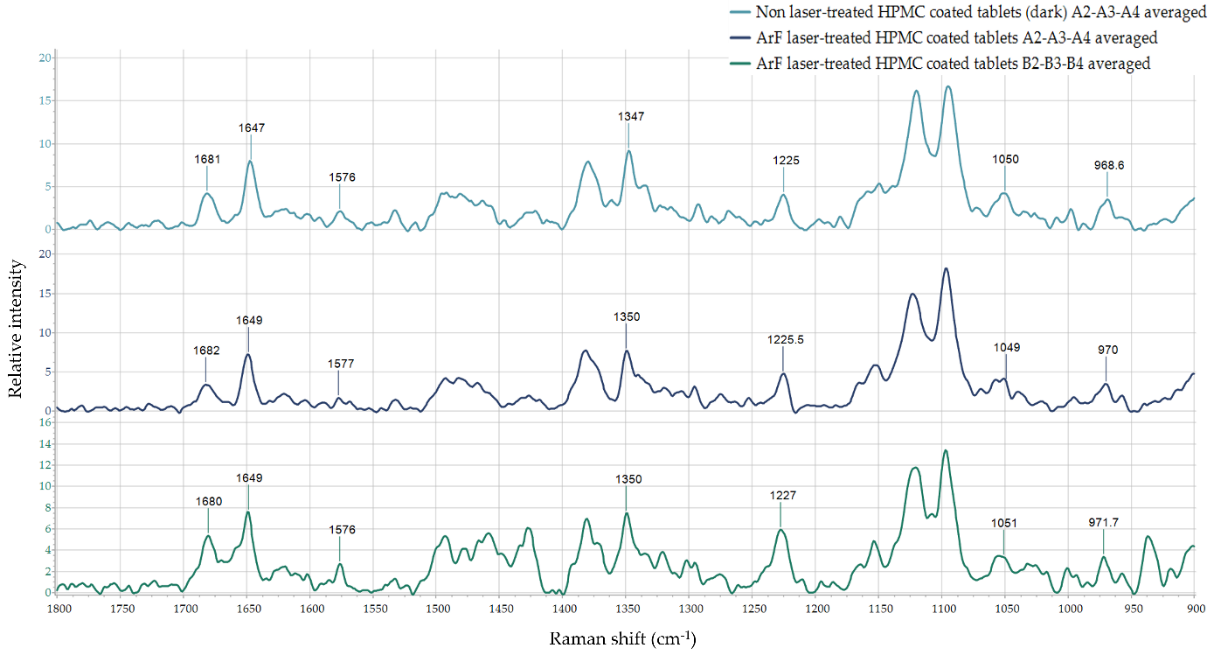

Figure S3. Point-specific averaged Raman spectra recorded directly from nifedipine crystals within the tablet cores for the HPMC coated tablet (formulation II) in the case of the darkness, and the ArF laser treatment in the regions A and B, respectively.

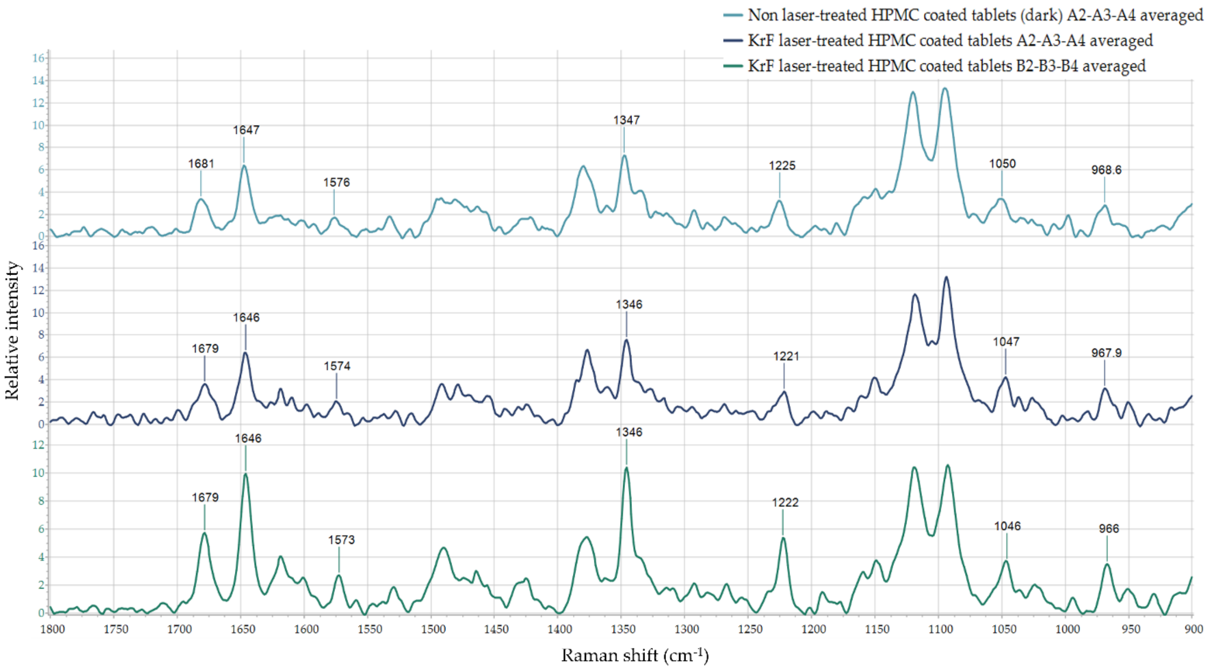

Figure S4. Point-specific averaged Raman spectra recorded directly from nifedipine crystals within the tablet cores for the HPMC coated tablet (formulation II) in the case of the darkness, and the KrF laser treatment in the regions A and B, respectively.

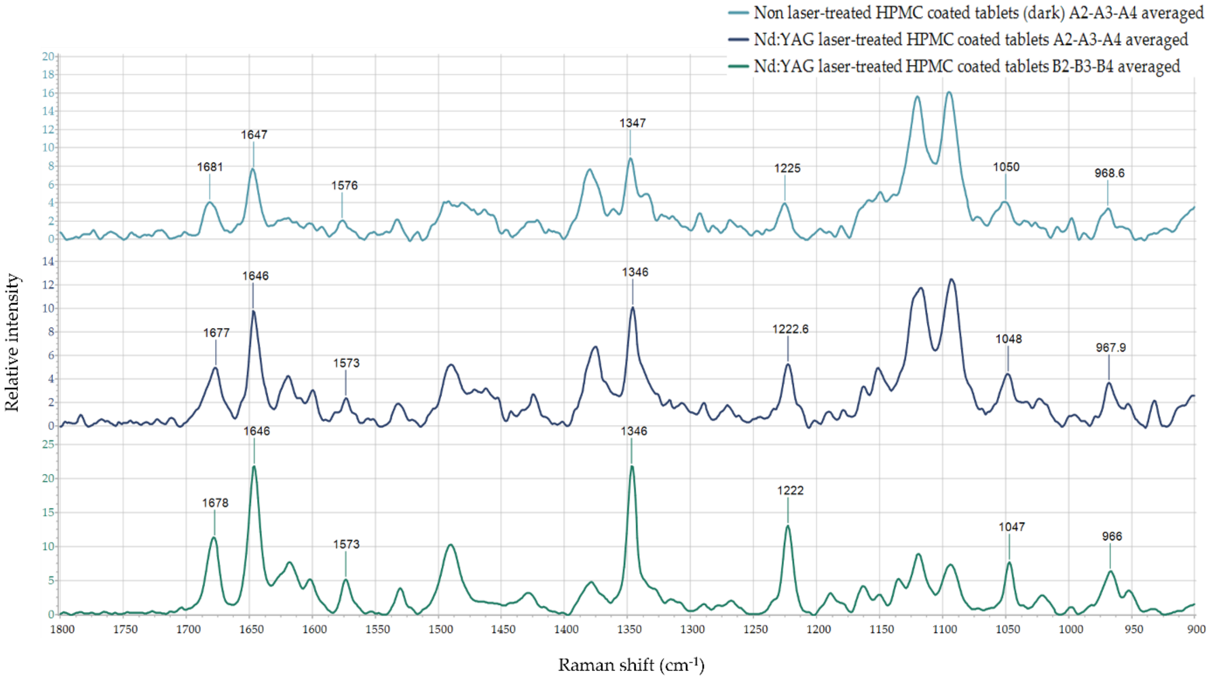

Figure S5. Point-specific averaged Raman spectra recorded directly from nifedipine crystals within the tablet cores for the HPMC coated tablet (formulation II) in the case of the darkness, and the Nd:YAG laser treatment in the regions A and B, respectively.

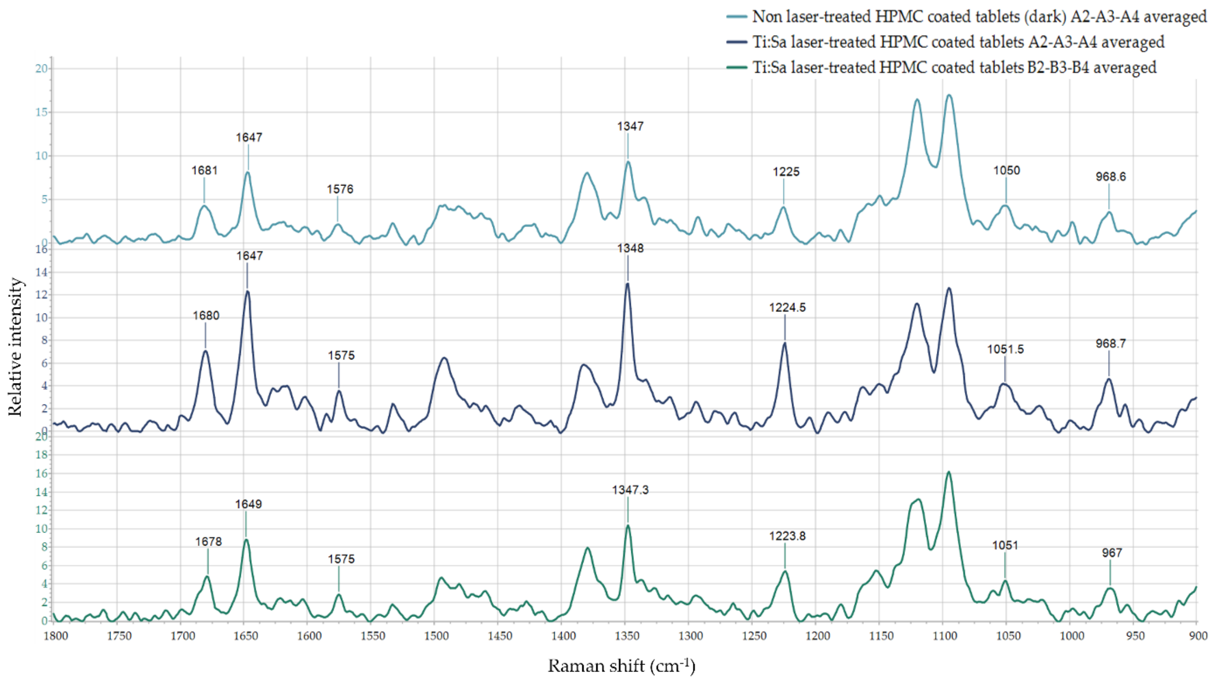

Figure S6. Point-specific averaged Raman spectra recorded directly from nifedipine crystals within the tablet cores for the HPMC coated tablet in (formulation II) the case of the darkness, and the Ti:Sa laser treatment in the regions A and B, respectively.

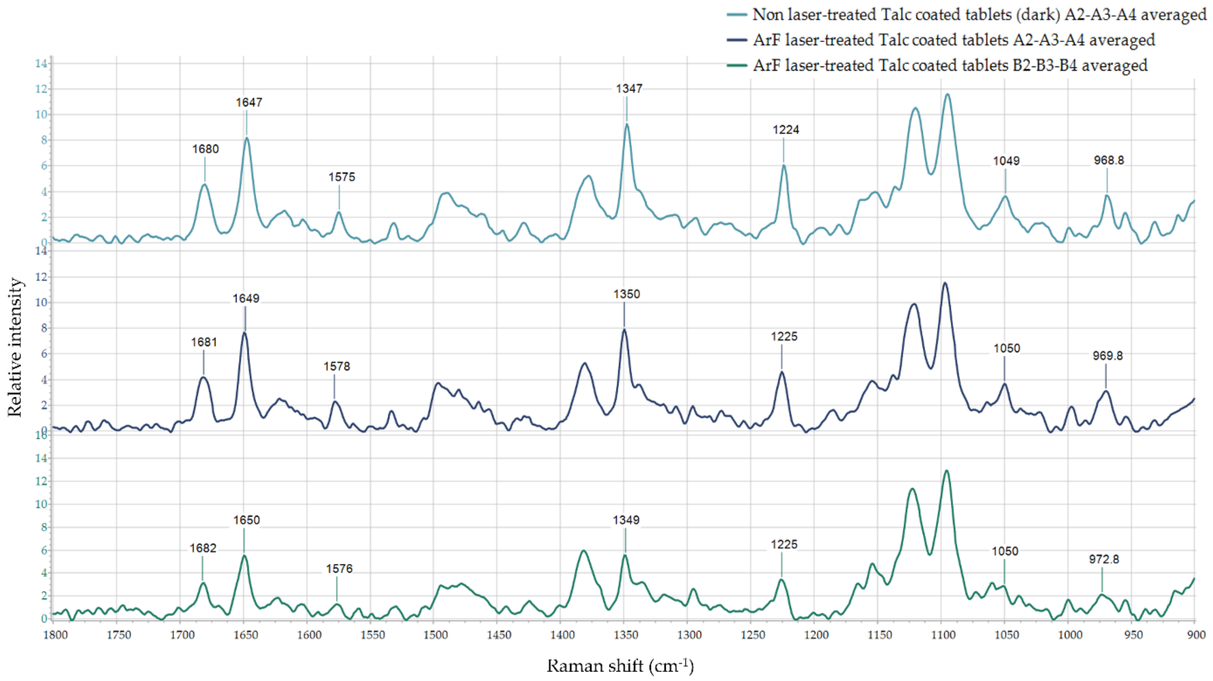

Figure S7. Point-specific averaged Raman spectra recorded directly from nifedipine crystals within the tablet cores for the talc coated tablet (formulation III) in the case of the darkness, and the ArF laser treatment in the regions A and B, respectively.

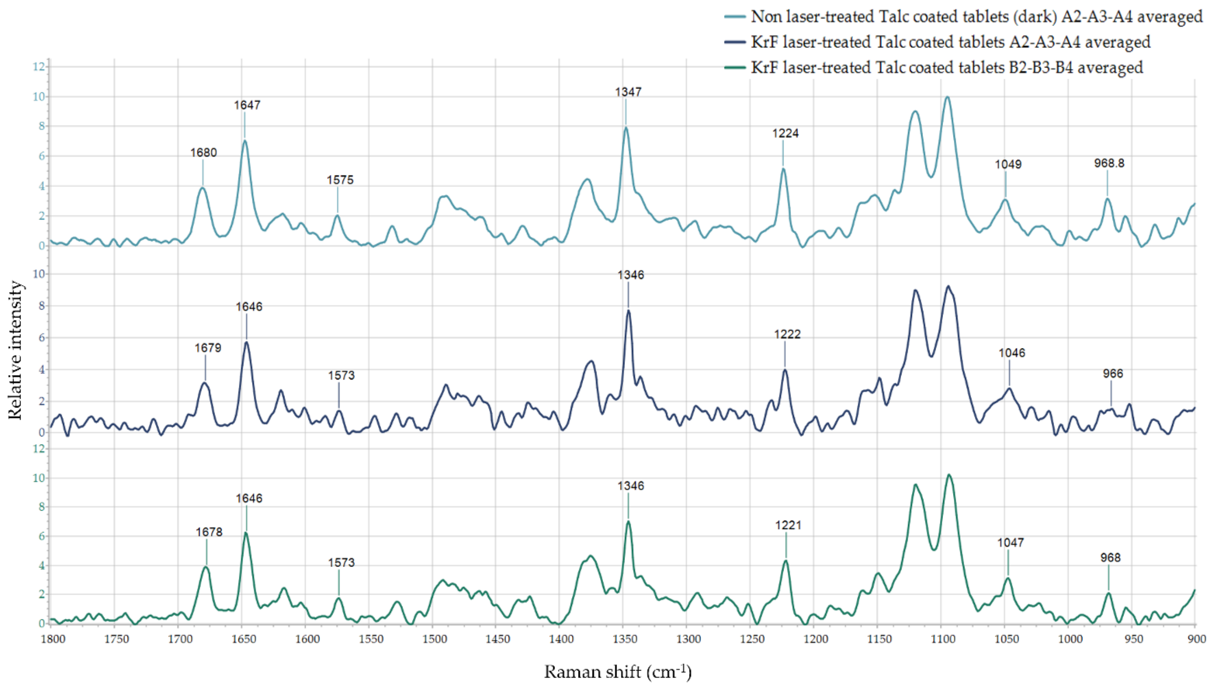

Figure S8. Point-specific averaged Raman spectra recorded directly from nifedipine crystals within the tablet cores for the talc coated tablet (formulation III) in the case of the darkness, and the KrF laser treatment in the regions A and B, respectively.

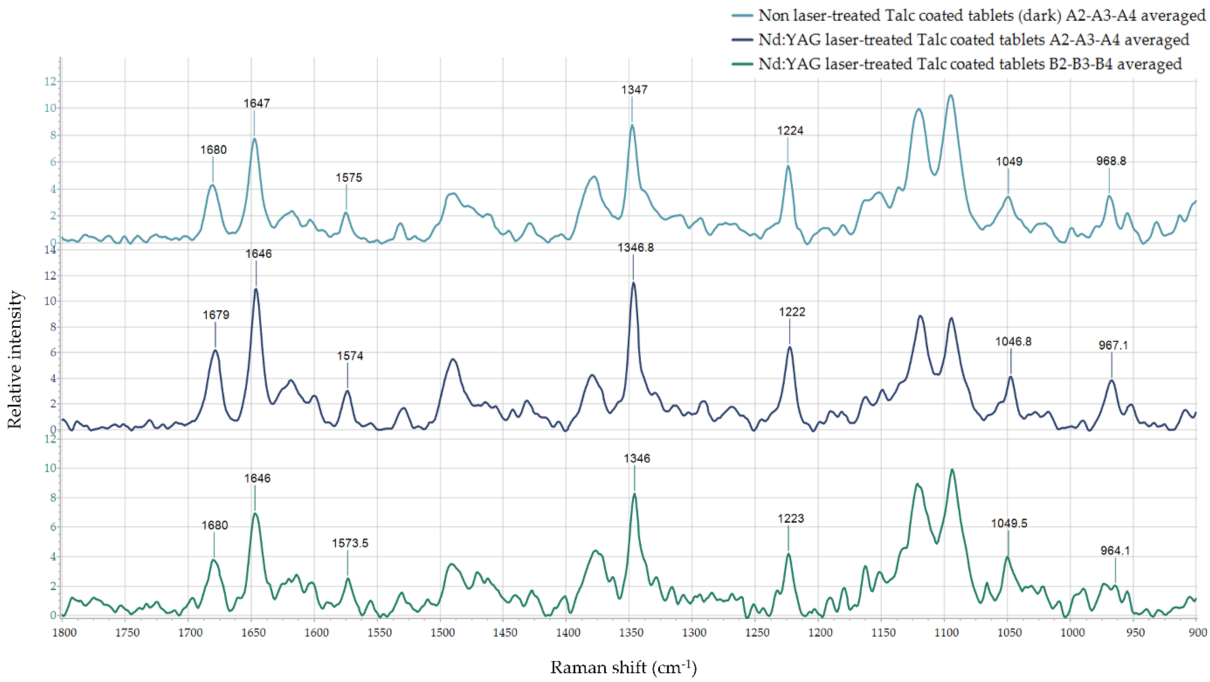

Figure S9. Point-specific averaged Raman spectra recorded directly from nifedipine crystals within the tablet cores for the talc coated tablet (formulation III) in the case of the darkness, and the Nd:YAG laser treatment in the regions A and B, respectively.

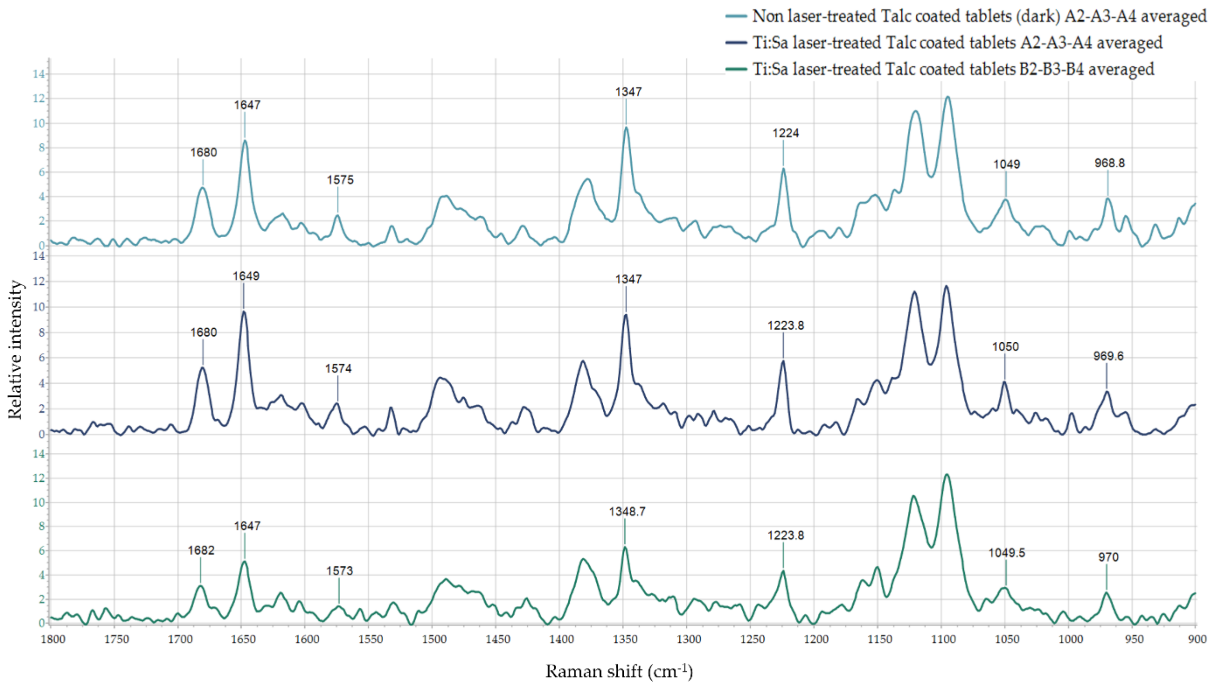

Figure S10. Point-specific averaged Raman spectra recorded directly from nifedipine crystals within the tablet cores for the talc coated tablet (formulation III) in the case of the darkness, and the Ti:Sa laser treatment in the regions A and B, respectively.

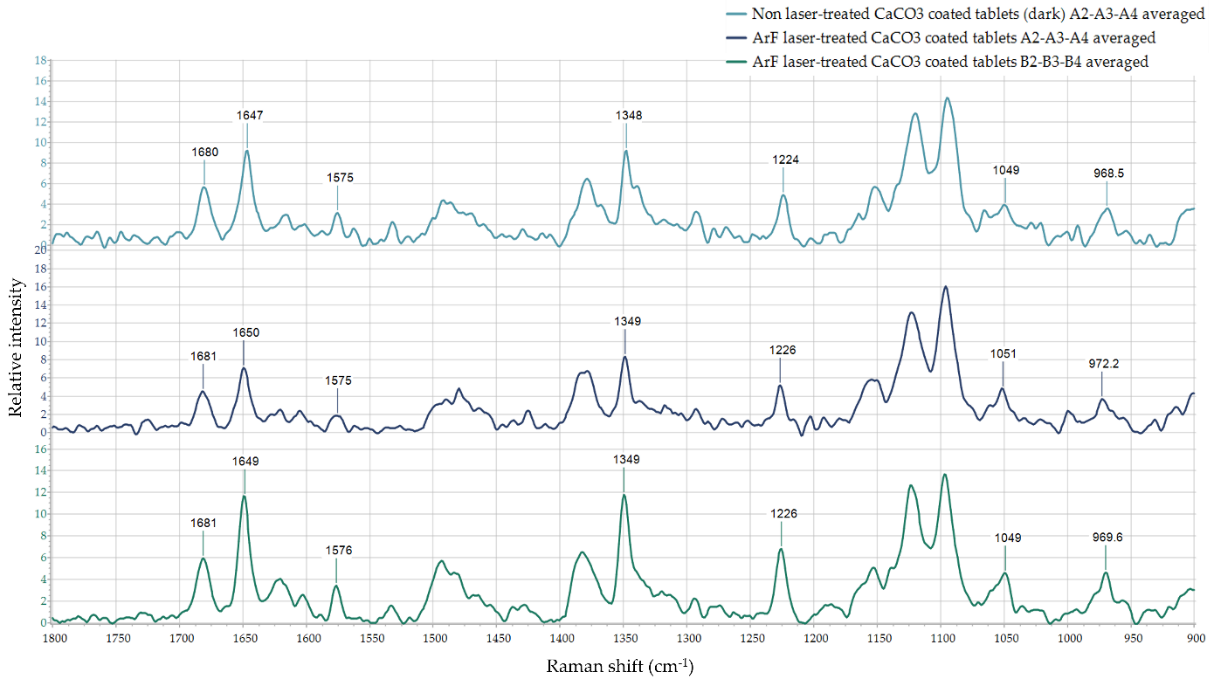

Figure S11. Point-specific averaged Raman spectra recorded directly from nifedipine crystals within the tablet cores for the $CaCO_3$ coated tablet (formulation IV) in the case of the darkness, and the ArF laser treatment in the regions A and B, respectively.

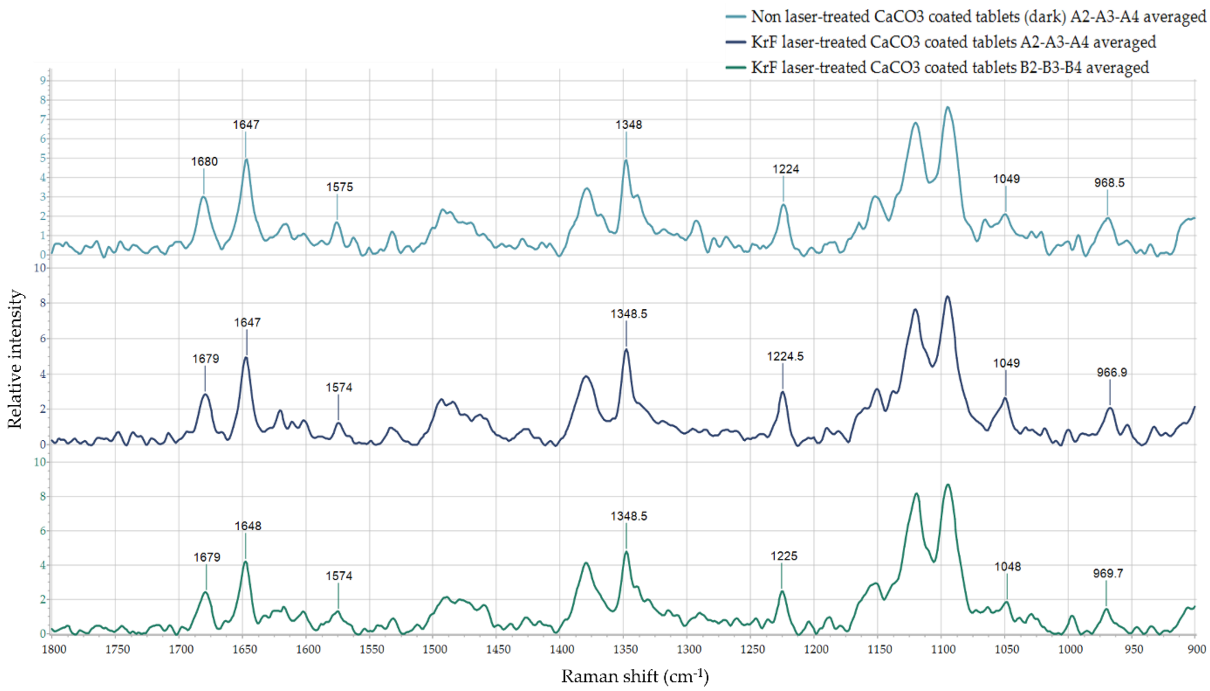

Figure S12. Point-specific averaged Raman spectra recorded directly from nifedipine crystals within the tablet cores for the $CaCO_3$ coated tablet (formulation IV) in the case of the darkness, and the KrF laser treatment in the regions A and B, respectively.

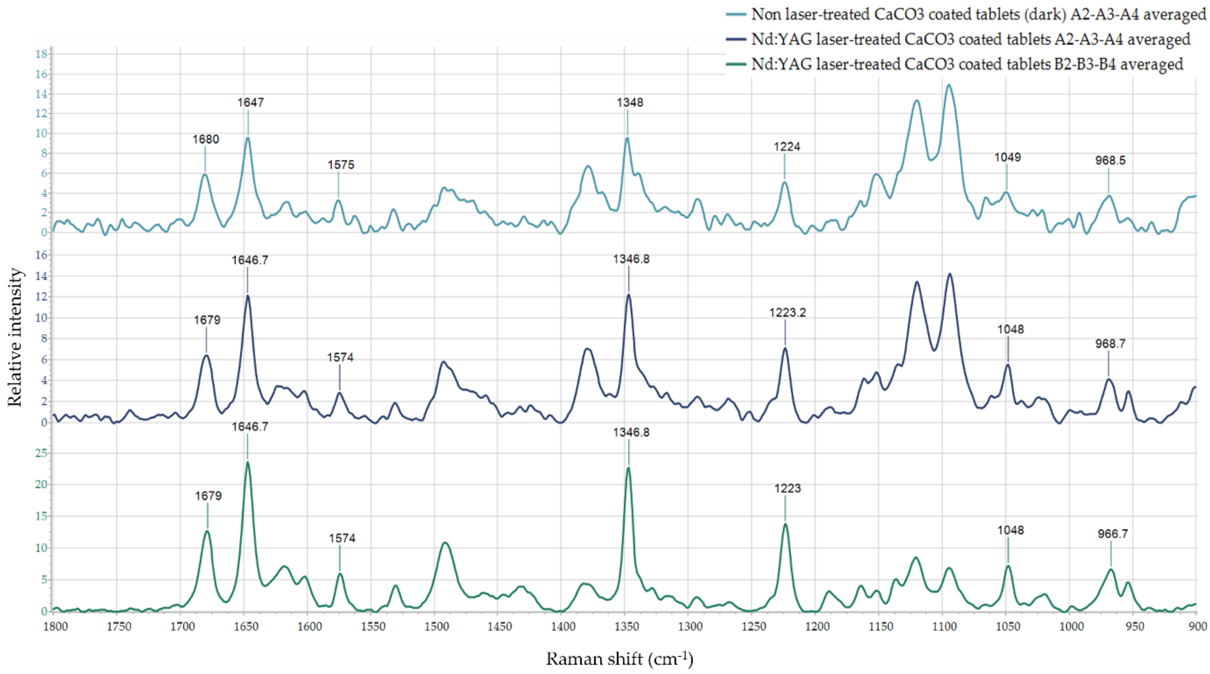

Figure S13. Point-specific averaged Raman spectra recorded directly from nifedipine crystals within the tablet cores for the $CaCO_3$ coated tablet (formulation IV) in the case of the darkness, and the Nd:YAG laser treatment in the regions A and B, respectively.

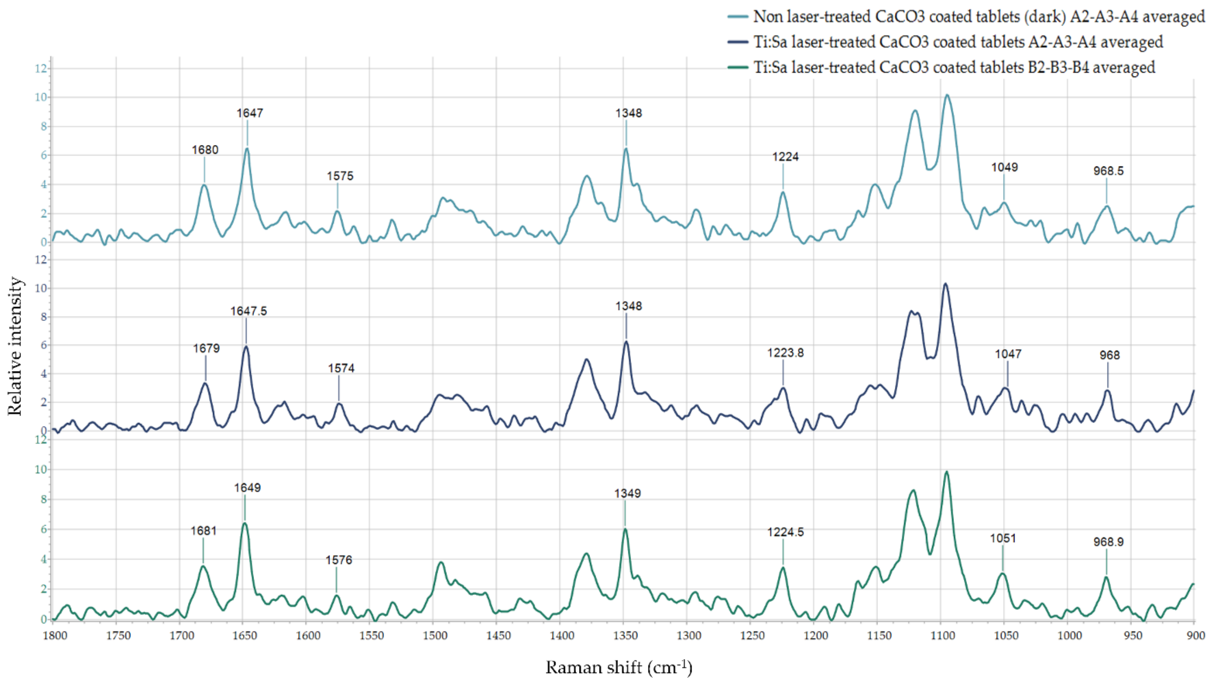

Figure S14. Point-specific averaged Raman spectra recorded directly from nifedipine crystals within the tablet cores for the $CaCO_3$ coated tablet (formulation IV) in the case of the darkness, and the Ti:Sa laser treatment in the regions A and B, respectively.

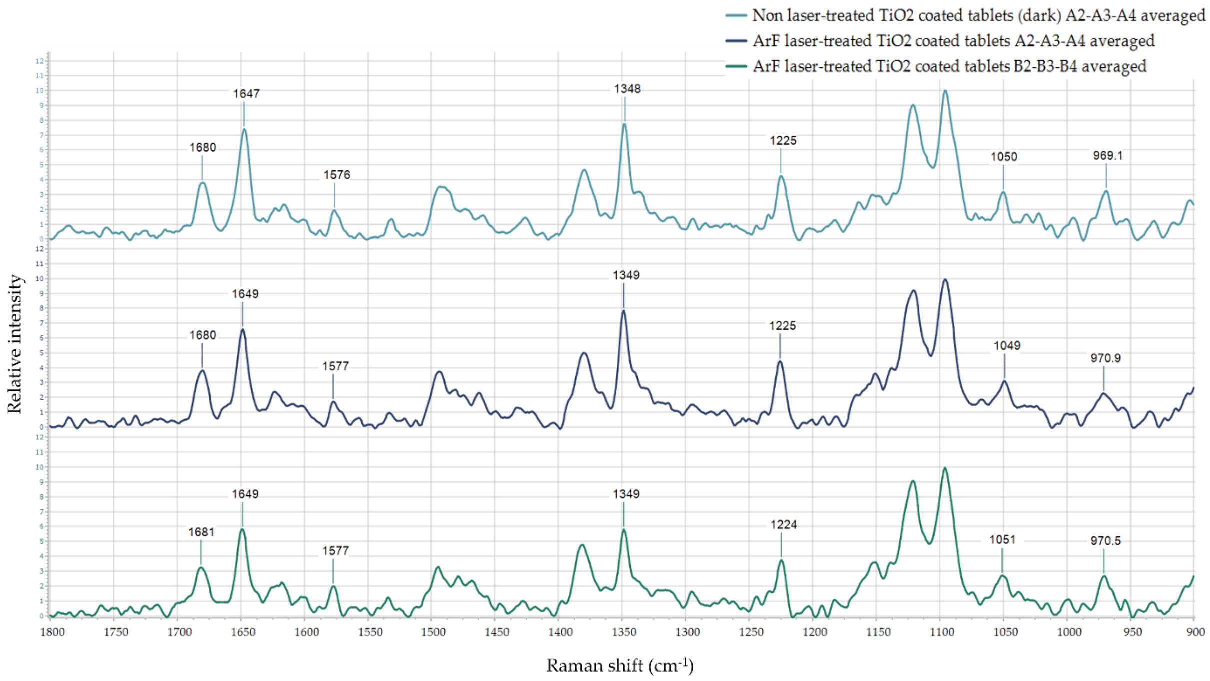

Figure S15. Point-specific averaged Raman spectra recorded directly from nifedipine crystals within the tablet cores for the TiO_2 coated tablet (formulation V) in the case of the darkness, and the ArF laser treatment in the regions A and B, respectively.

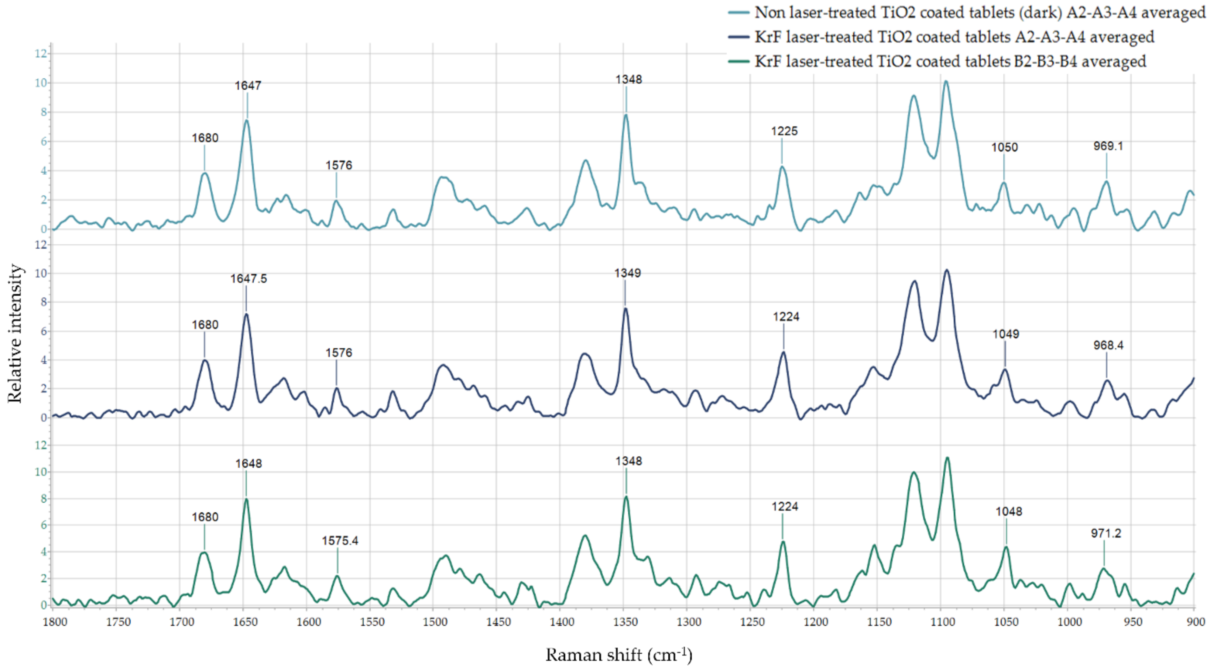

Figure S16. Point-specific averaged Raman spectra recorded directly from nifedipine crystals within the tablet cores for the TiO_2 coated tablet (formulation V) in the case of the darkness, and the KrF laser treatment in the regions A and B, respectively.

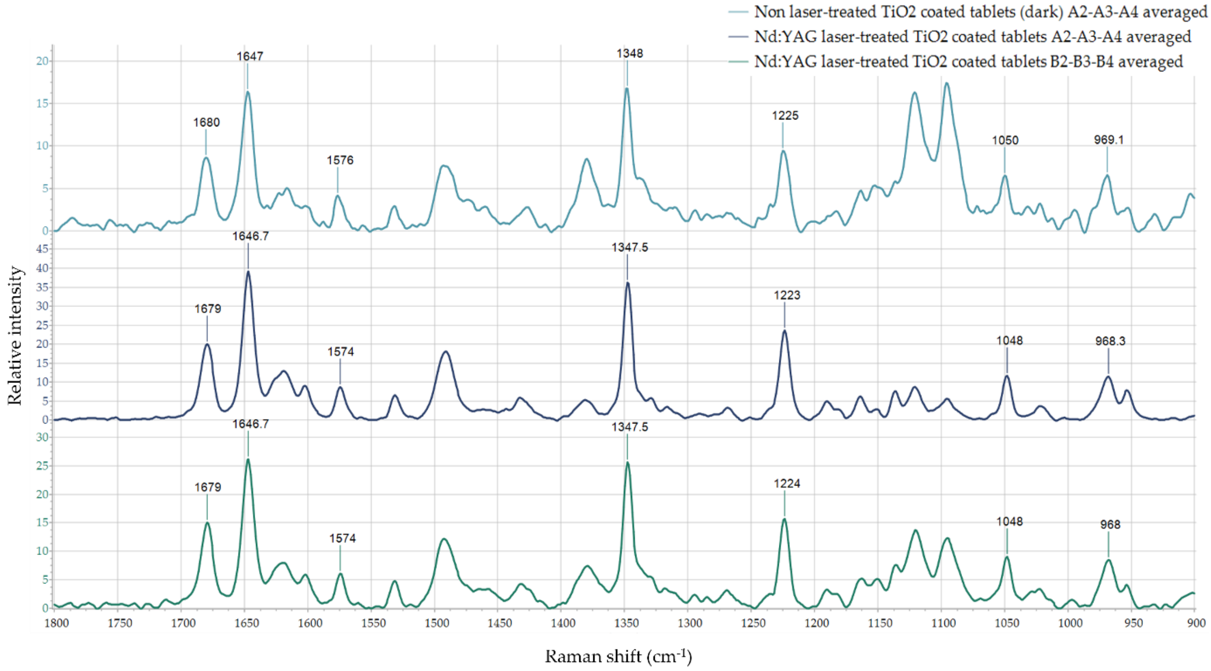

Figure S17. Point-specific averaged Raman spectra recorded directly from nifedipine crystals within the tablet cores for the TiO_2 coated tablet (formulation V) in the case of the darkness, and the Nd:YAG laser treatment in the regions A and B, respectively.

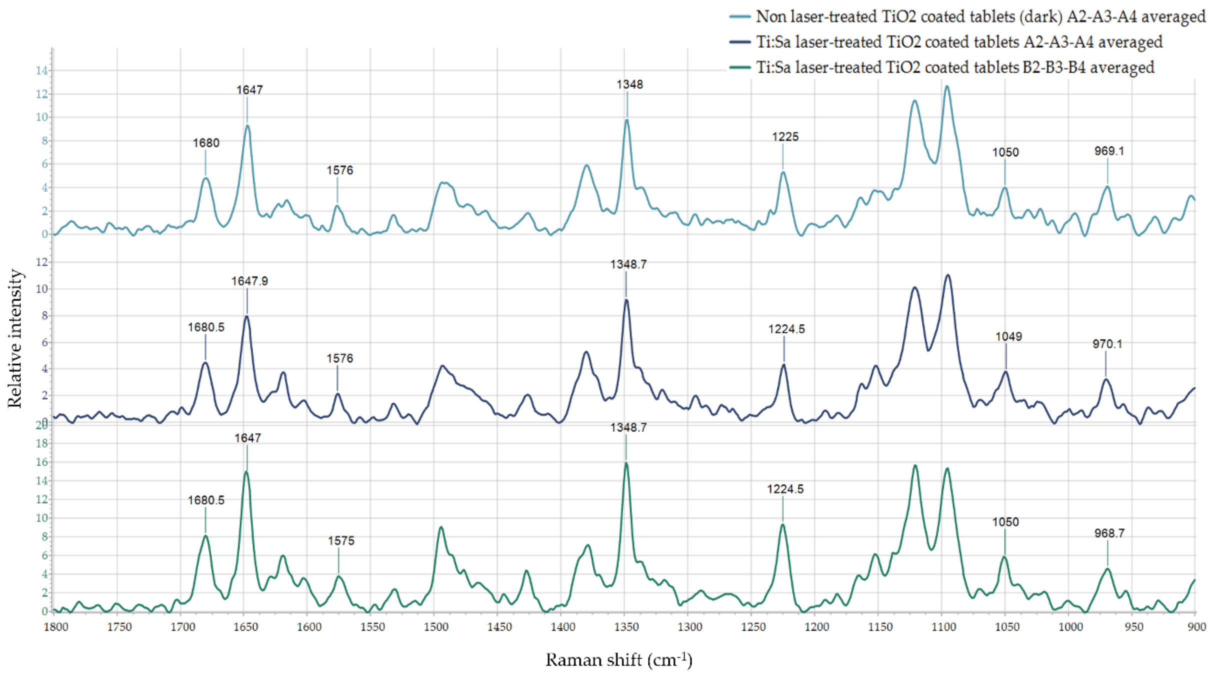

Figure S18. Point-specific averaged Raman spectra recorded directly from nifedipine crystals within the tablet cores for the TiO_2 coated tablet (formulation V) in the case of the darkness, and the Ti:Sa laser treatment in the regions A and B, respectively.

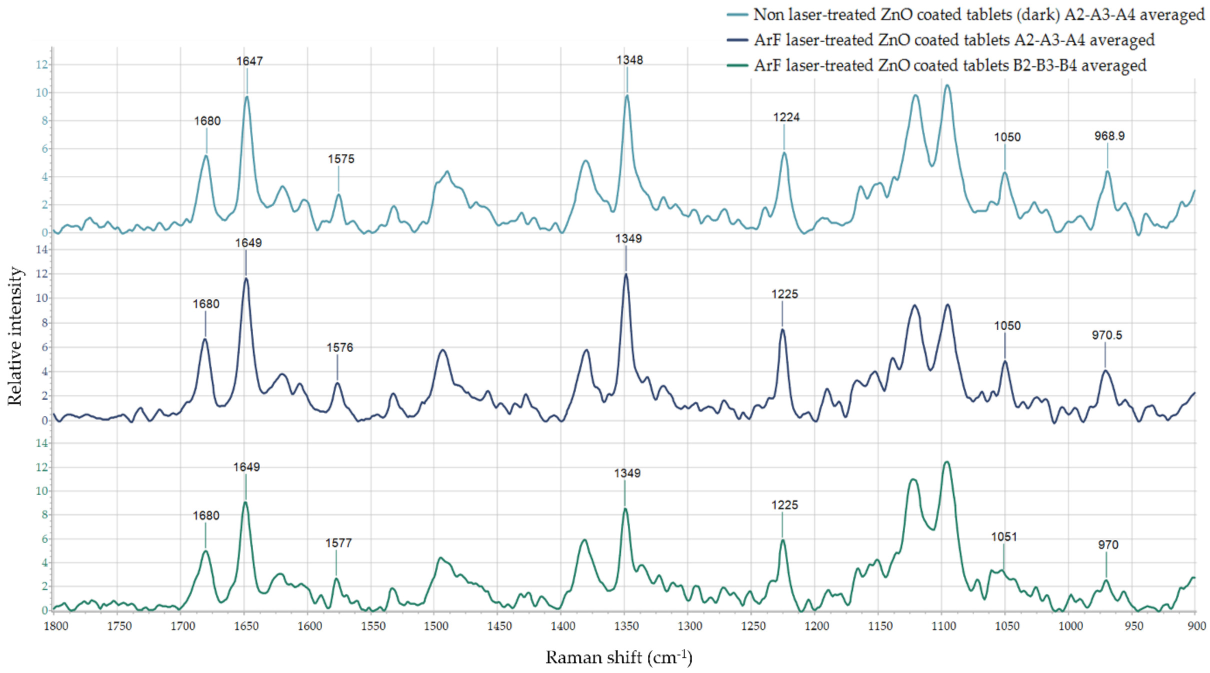

Figure S19. Point-specific averaged Raman spectra recorded directly from nifedipine crystals within the tablet cores for the ZnO coated tablet (formulation VI) in the case of the darkness, and the ArF laser treatment in the regions A and B, respectively.

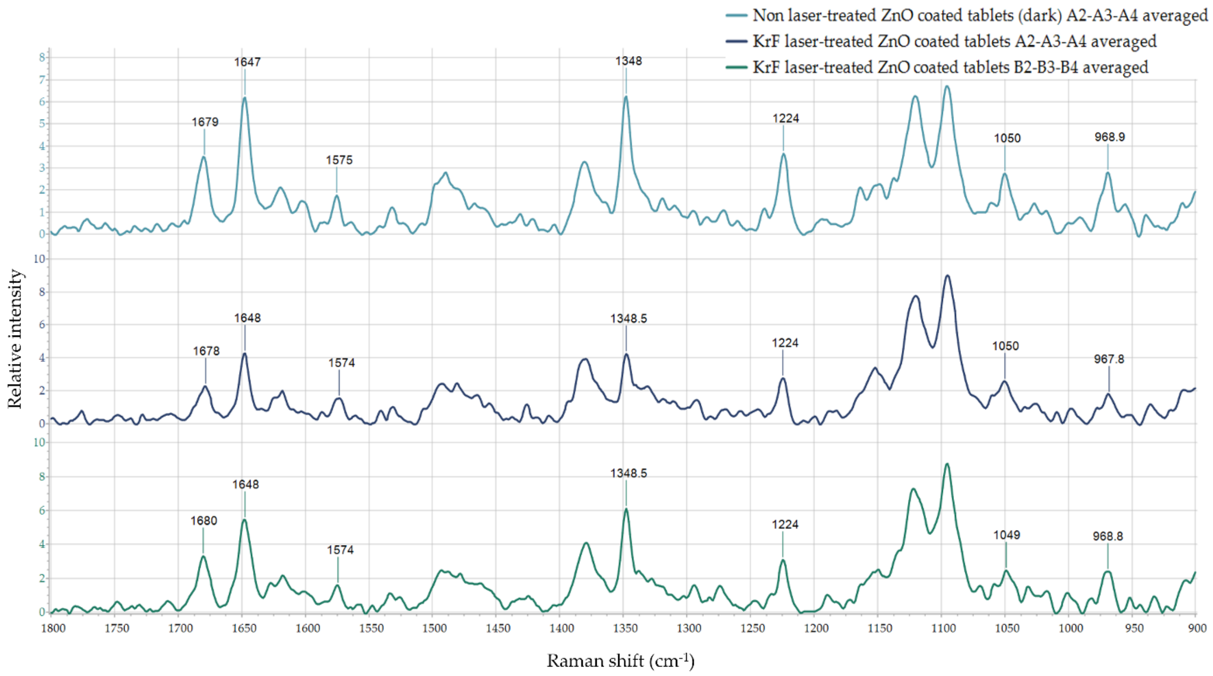

Figure S20. Point-specific averaged Raman spectra recorded directly from nifedipine crystals within the tablet cores for the ZnO coated tablet (formulation VI) in the case of the darkness, and the KrF laser treatment in the regions A and B, respectively.

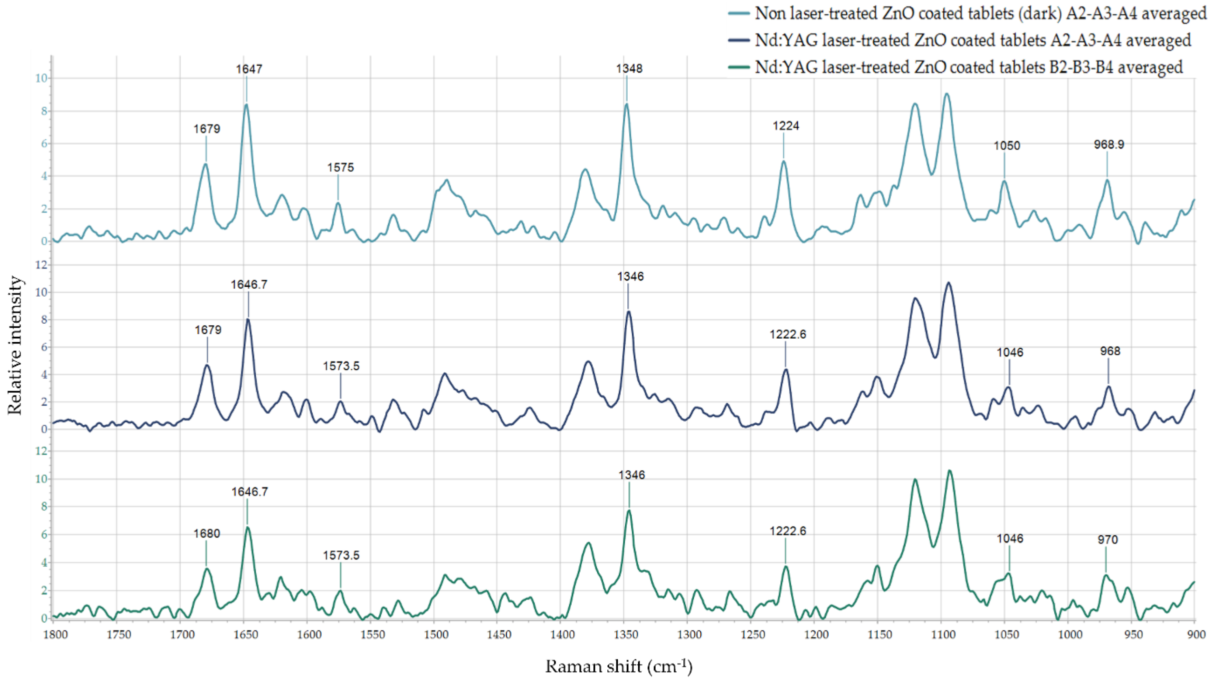

Figure S21. Point-specific averaged Raman spectra recorded directly from nifedipine crystals within the tablet cores for the ZnO coated tablet (formulation VI) in the case of the darkness, and the Nd:YAG laser treatment in the regions A and B, respectively.

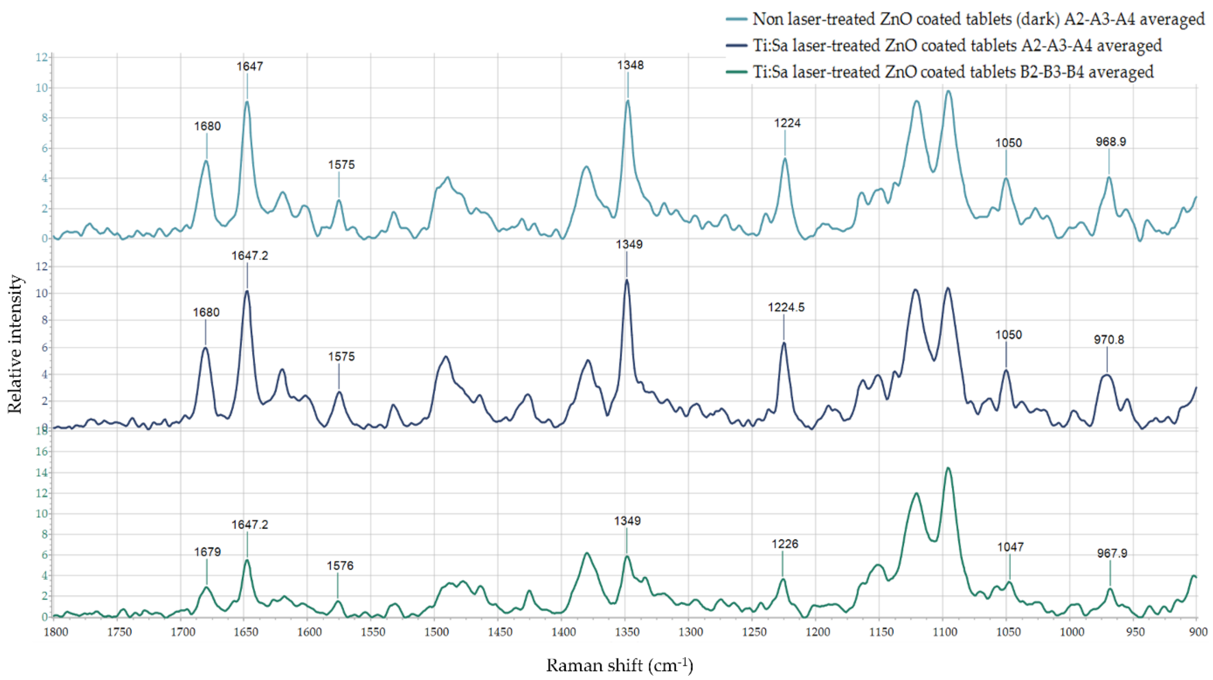

Figure S22. Point-specific averaged Raman spectra recorded directly from nifedipine crystals within the tablet cores for the ZnO coated tablet (formulation VI) in the case of the darkness, and the Ti:Sa laser treatment in the regions A and B, respectively.

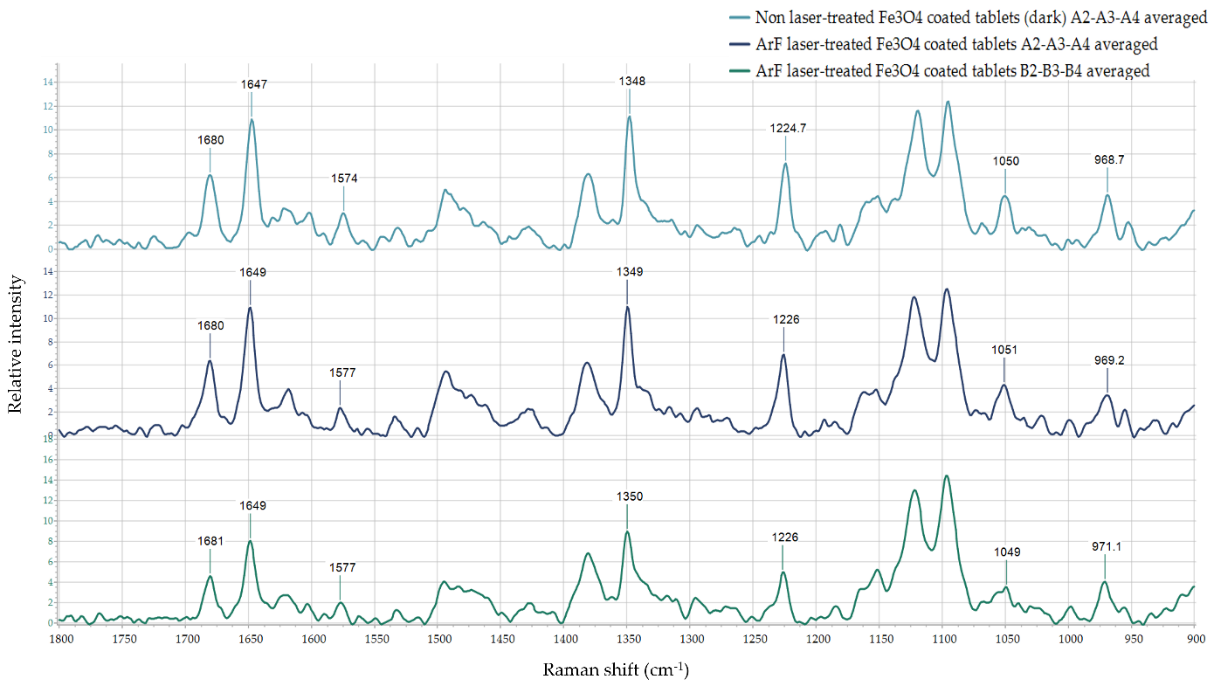

Figure S23. Point-specific averaged Raman spectra recorded directly from nifedipine crystals within the tablet cores for the Fe_3O_4 coated tablet (formulation VII) in the case of the darkness, and the ArF laser treatment in the regions A and B, respectively.

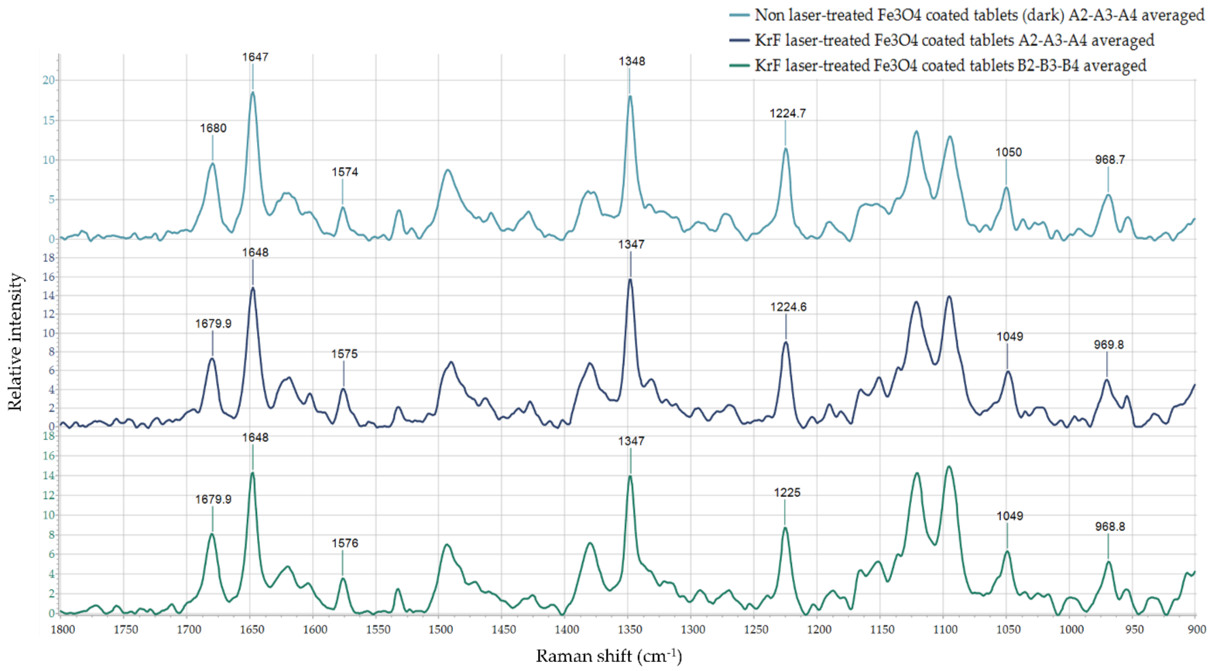

Figure S24. Point-specific averaged Raman spectra recorded directly from nifedipine crystals within the tablet cores for the Fe_3O_4 coated tablet (formulation VII) in the case of the darkness, and the KrF laser treatment in the regions A and B, respectively.

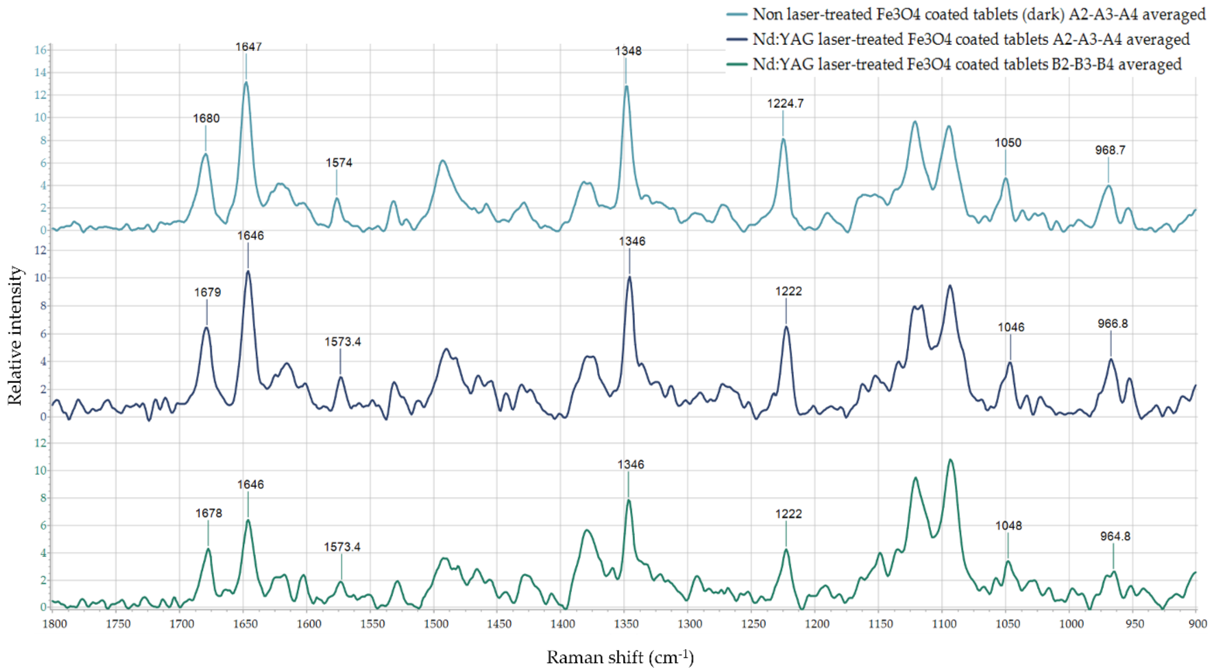

Figure S25. Point-specific averaged Raman spectra recorded directly from nifedipine crystals within the tablet cores for the Fe_3O_4 coated tablet (formulation VII) in the case of the darkness, and the Nd:YAG laser treatment in the regions A and B, respectively.

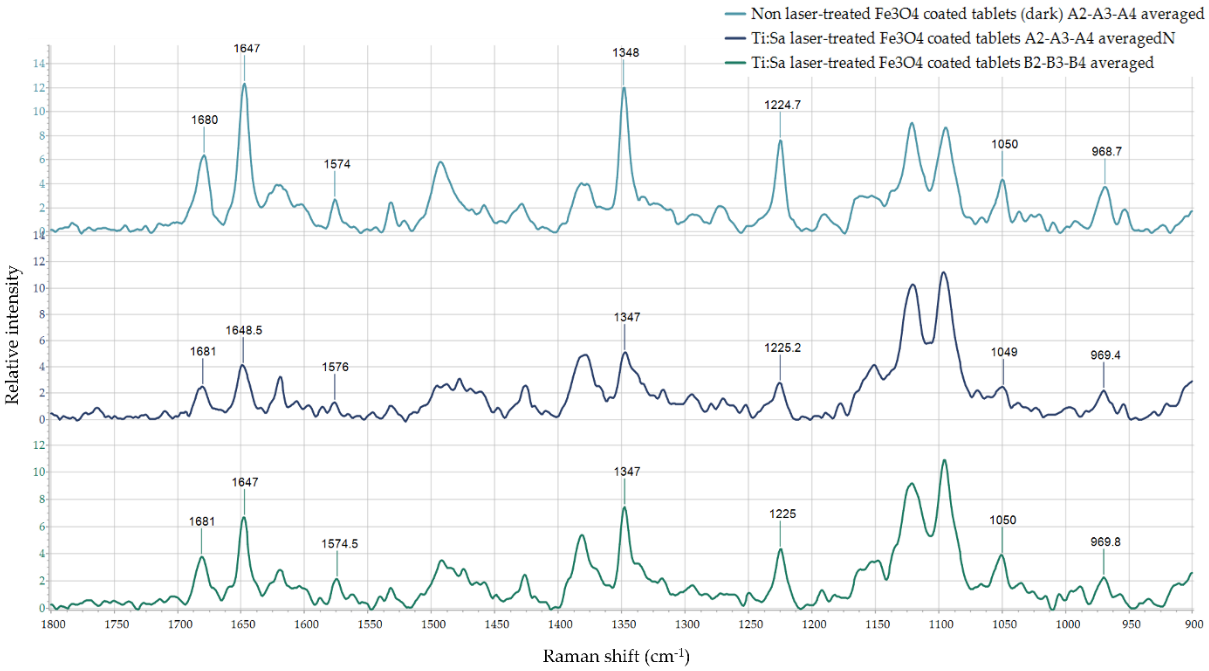

Figure S26. Point-specific averaged Raman spectra recorded directly from nifedipine crystals within the tablet cores for the Fe_3O_4 coated tablet (formulation VII) in the case of the darkness, and the Ti:Sa laser treatment in the regions A and B, respectively.

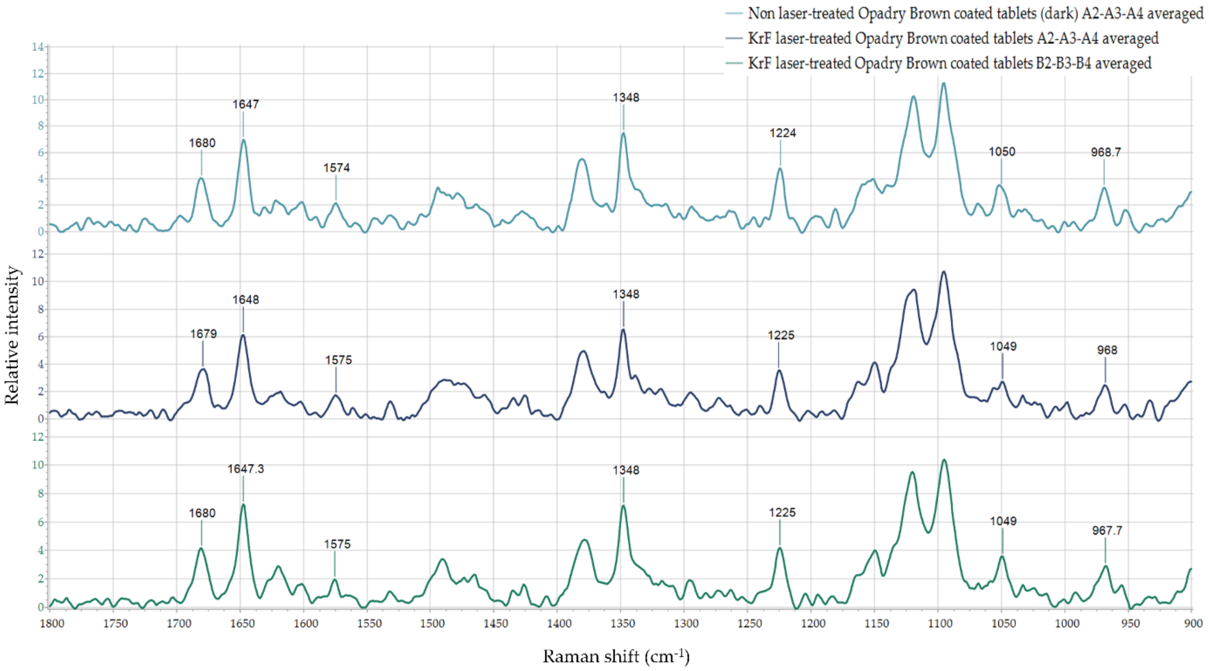

Figure S27. Point-specific averaged Raman spectra recorded directly from nifedipine crystals within the tablet cores for the Opadry® TC Brown coated tablet (formulation VIII) in the case of the darkness, and the KrF laser treatment in the regions A and B, respectively.

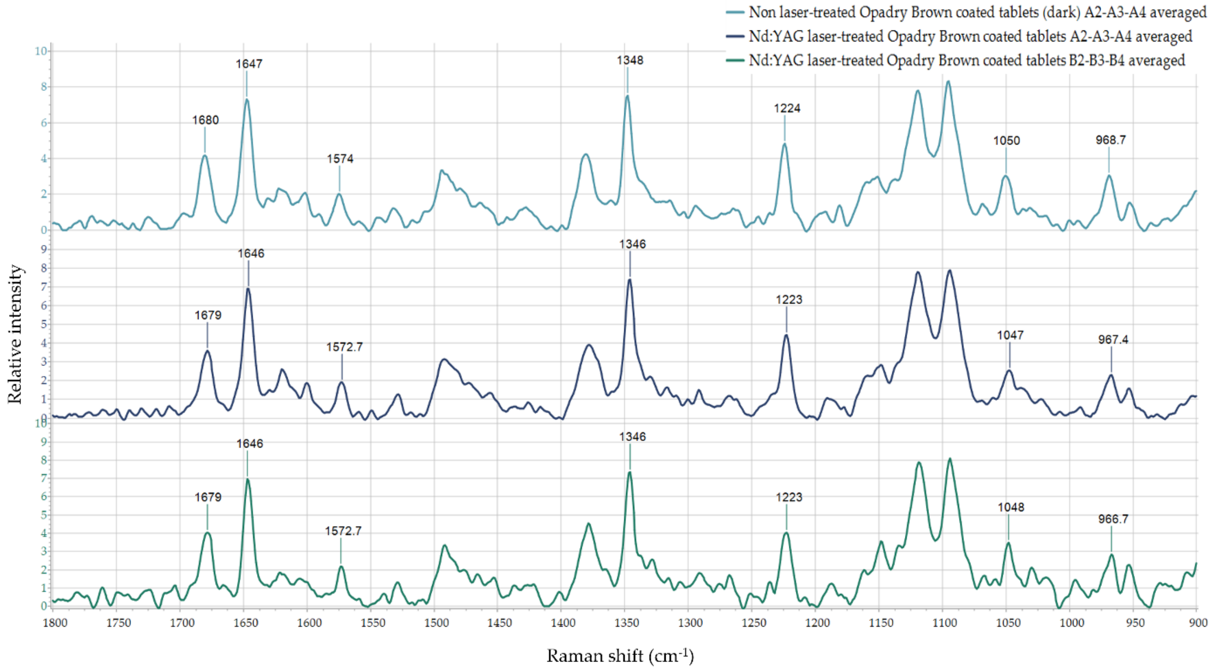

Figure S28. Point-specific averaged Raman spectra recorded directly from nifedipine crystals within the tablet cores for the Opadry® TC Brown coated tablet (formulation VIII) in the case of the darkness, and the Nd:YAG laser treatment in the regions A and B, respectively.

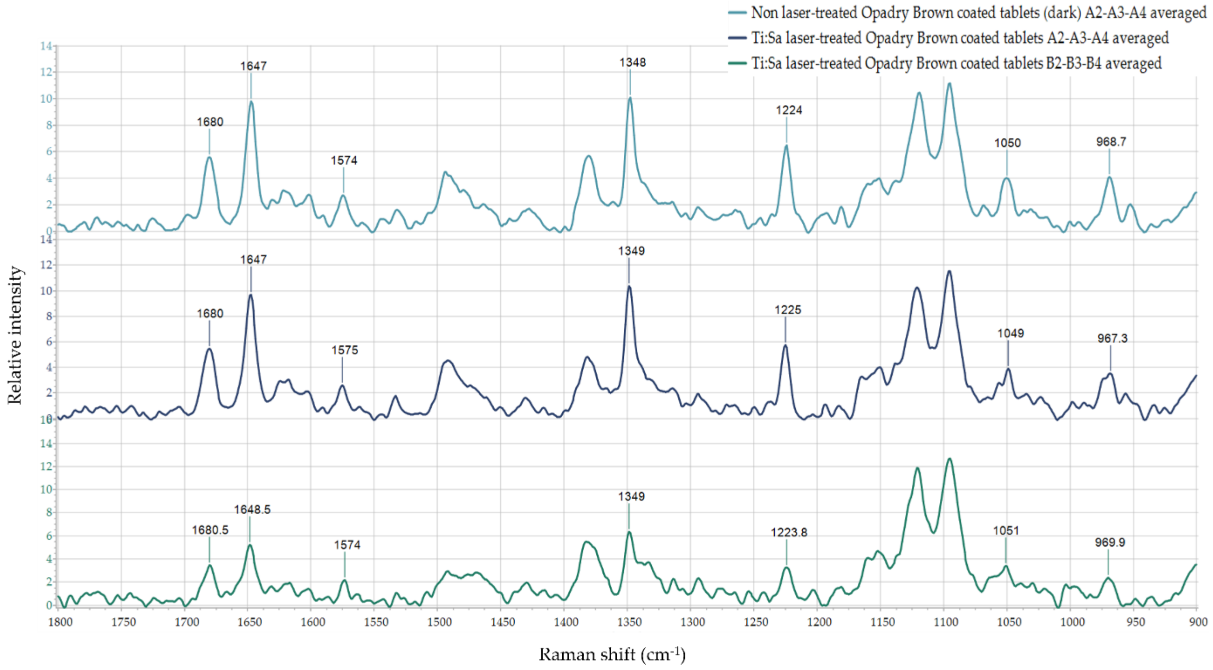

Figure S29. Point-specific averaged Raman spectra recorded directly from nifedipine crystals within the tablet cores for the Opadry® TC Brown coated tablet (formulation VIII) in the case of the darkness, and the Ti:Sa laser treatment in the regions A and B, respectively.

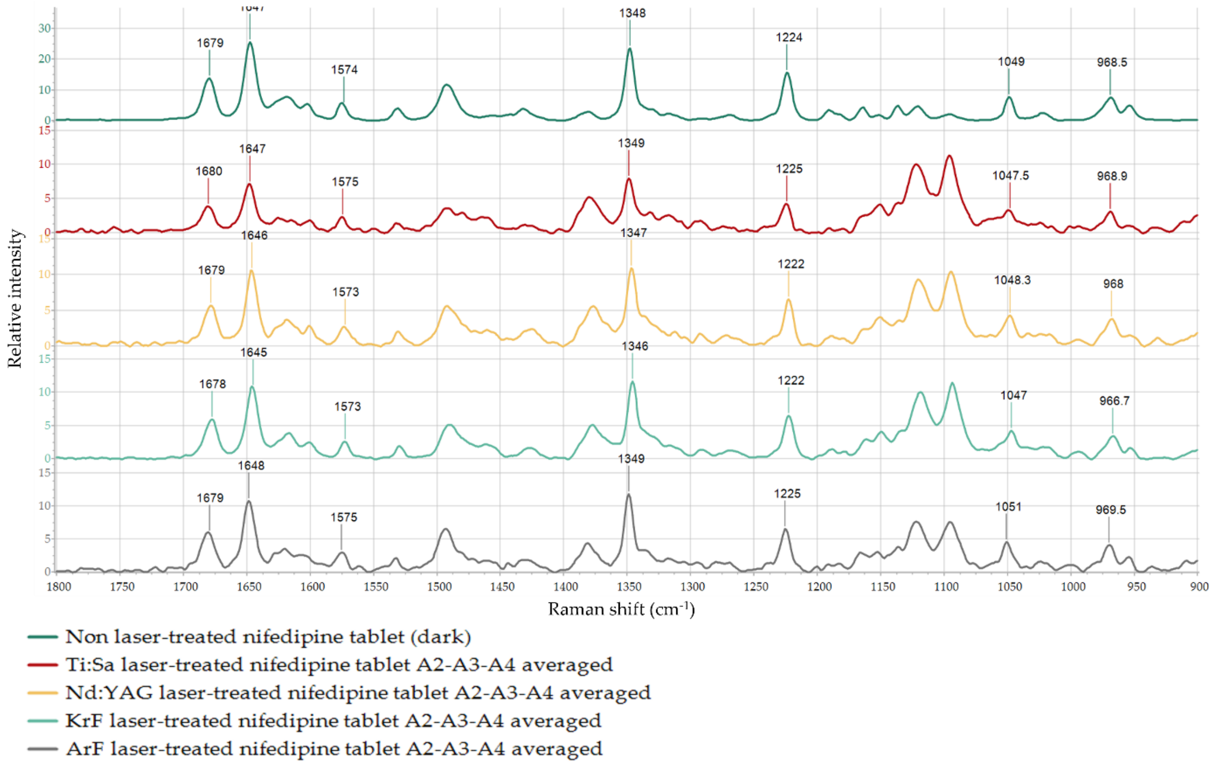

Figure S30. Point-specific averaged Raman spectra recorded directly from nifedipine crystals within the tablet cores in the region (A) for a dark-stored, Ti:Sa laser-treated, Nd:YAG laser-treated, KrF laser-treated, and ArF laser-treated uncoated tablet (formulation I), respectively.

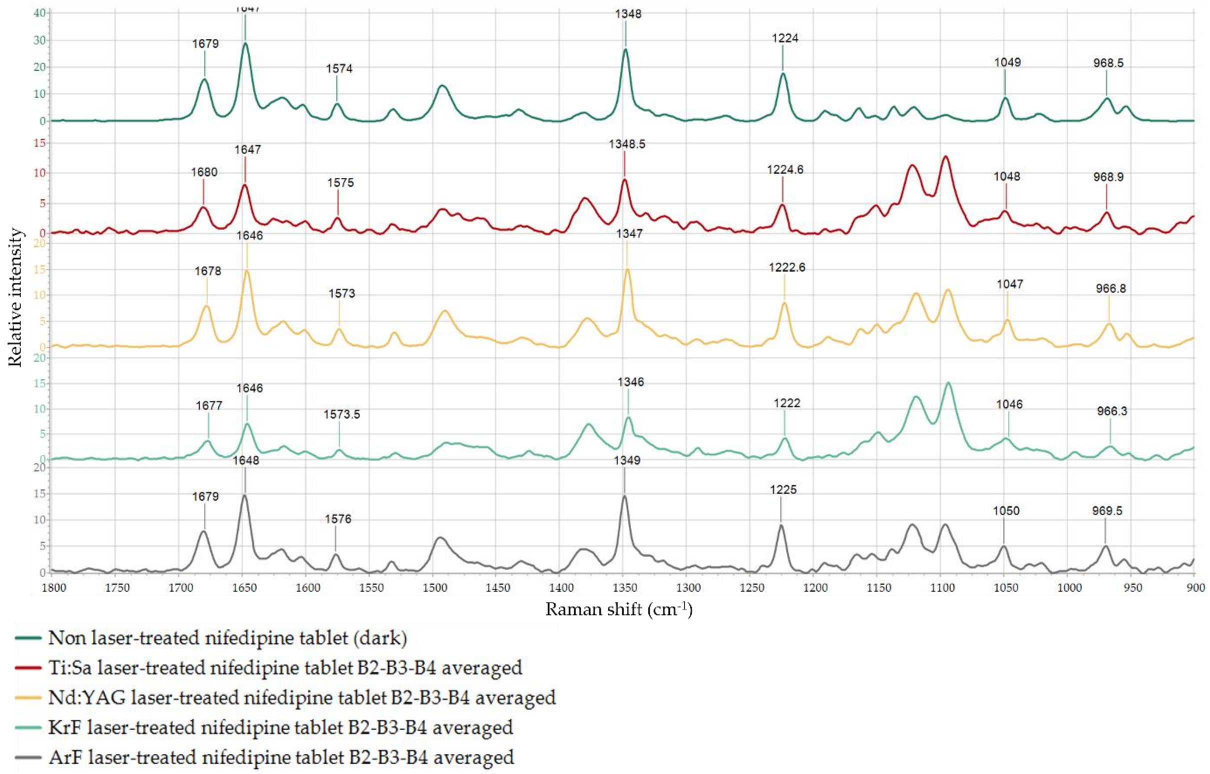

Figure S31. Point-specific averaged Raman spectra recorded directly from nifedipine crystals within the tablet cores in the region (B) for a dark-stored, Ti:Sa laser-treated, Nd:YAG laser-treated, KrF laser-treated, and ArF laser-treated uncoated tablet (formulation I), respectively.

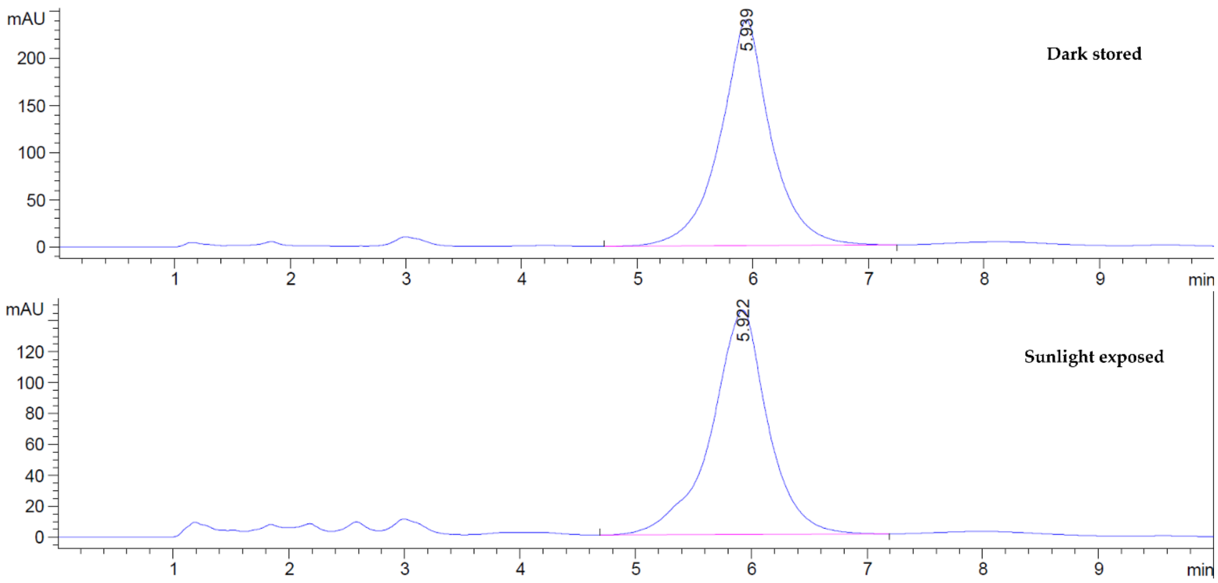

Figure S32. HPLC chromatograms of nifedipine uncoated tablet (formulation I) before and after sunlight exposure over 6 months under the same studied conditions (HPLC conditions are seen in the text).

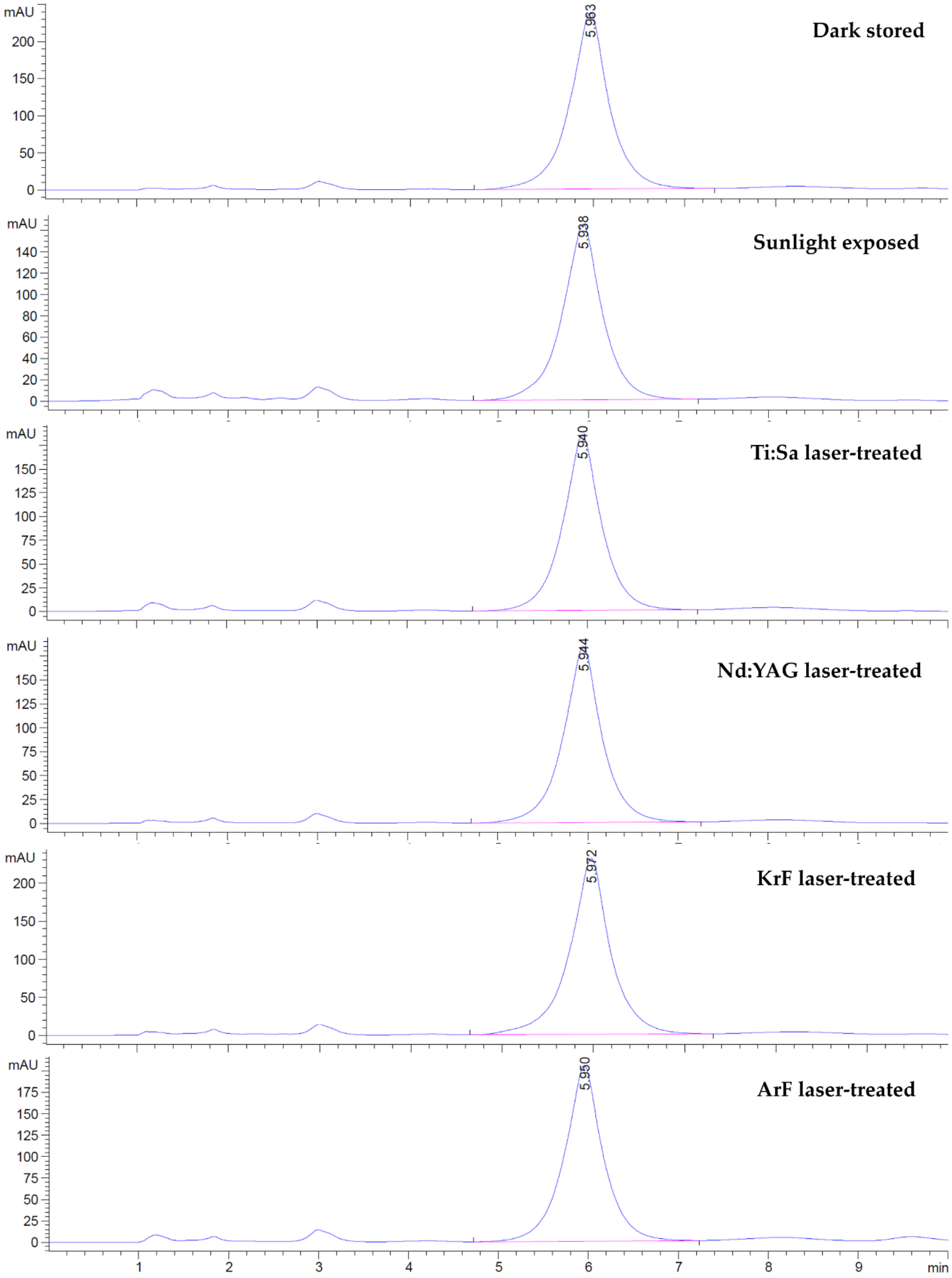

Figure S33. HPLC chromatograms of formulation VII (optimum coating formulation) before, after prolonged exposure of sunlight over 6 months, and short-term of Ti:Sa laser, Nd:YAG laser, KrF laser and ArF laser treatments under the same studied conditions, respectively (HPLC conditions are seen in the text).

Table S3: Overall statistical results of sunlight exposure

Effect	Univariate Tests of Significance for Drug content (%) (Sunlight in statistics) Sigma-restricted parameterization Effective hypothesis decomposition				
	SS	Degr. of	MS	F	p
Intercept	789681,4	1	789681,4	30802,90	0,000000
Sample	2649,2	3	883,1	34,45	0,000000
Formulation	1581,6	7	225,9	8,81	0,000000
Sample*Formulation	339,7	21	16,2	0,63	0,879855
Error	1640,7	64	25,6		

Table S4: Post-hoc comparison of sunlight exposure times

Cell No.	LSD test; variable Drug content (%) (Sunlight in statistics) Probabilities for Post Hoc Tests Error: Between MS = 25,637, df = 64,000				
	Sample	{1}	{2}	{3}	{4}
1	NC		0,000000	0,000000	0,000000
2	4 months	0,000000		0,265297	0,000672
3	5 months	0,000000	0,265297		0,016958
4	6 months	0,000000	0,000672	0,016958	

Table S5: Post-hoc comparison of various coating formulations against sunlight exposure

Cell No.	LSD test; variable Drug content (%) (Sunlight in statistics) Probabilities for Post Hoc Tests Error: Between MS = 25,637, df = 64,000								
	Formulation	{1}	{2}	{3}	{4}	{5}	{6}	{7}	{8}
1	I		0,225175	0,011760	0,003730	0,000160	0,004618	0,000000	0,000000
2	II	0,225175		0,175825	0,078870	0,006979	0,091921	0,000029	0,000005
3	III	0,011760	0,175825		0,678130	0,160778	0,733363	0,002623	0,000548
4	IV	0,003730	0,078870	0,678130		0,320125	0,940624	0,008525	0,002001
5	V	0,000160	0,006979	0,160778	0,320125		0,285629	0,091675	0,029946
6	VI	0,004618	0,091921	0,733363	0,940624	0,285629		0,006954	0,001597
7	VII	0,000000	0,000029	0,002623	0,008525	0,091675	0,006954		0,613223
8	VIII	0,000000	0,000005	0,000548	0,002001	0,029946	0,001597	0,613223	

Table S6: Post-hoc comparison of sunlight exposure results

LSD test; variable Drug content (%) (Sunlight in statistics)
Probabilities for Post Hoc Tests
Error: Between MS = 25,637, df = 64,000

Cell No.	Sample	Formulation	{1}	{2}	{3}	{4}	{5}	{6}	{7}	{8}	{9}	{10}	{11}	{12}	{13}	{14}	{15}	{16}	{17}	{18}	{19}	{20}	{21}	{22}	{23}	{24}	{25}	{26}	{27}	{28}	{29}	{30}	{31}	{32}
1	NC	I		0,940368	0,310354	0,749417	0,261066	0,459733	0,462570	0,014781	0,000301	0,010614	0,044932	0,249935	0,597442	0,070271	0,348110	0,996006	0,000355	0,006764	0,022655	0,050130	0,227395	0,050130	0,996677	0,890466	0,000270	0,000862	0,005008	0,011721	0,006528	0,015480	0,097231	0,178803
2	NC	II	0,940368		0,276455	0,693504	0,231110	0,415894	0,418569	0,012165	0,000385	0,012928	0,053135	0,281576	0,650198	0,082198	0,311426	0,936386	0,000453	0,008303	0,027195	0,059129	0,257010	0,059129	0,943682	0,949835	0,000346	0,001090	0,006178	0,014251	0,008018	0,018723	0,112761	0,203681
3	NC	III	0,310354	0,276455		0,485350	0,911713	0,781293	0,777694	0,142996	0,000008	0,000521	0,003158	0,032662	0,125279	0,005659	0,938562	0,312719	0,000010	0,000302	0,001325	0,003638	0,028477	0,003638	0,308396	0,250016	0,000007	0,000027	0,000211	0,000588	0,000290	0,000826	0,008727	0,020205
4	NC	IV	0,749417	0,693504	0,485350		0,419168	0,673720	0,677144	0,032576	0,000102	0,004394	0,021012	0,143291	0,397656	0,034388	0,534575	0,753211	0,000121	0,002712	0,009958	0,023695	0,128592	0,023695	0,746265	0,647753	0,000091	0,000307	0,001968	0,004888	0,002611	0,006596	0,049320	0,097804
5	NC	V	0,261066	0,231110	0,911713	0,419168		0,697742	0,694277	0,174968	0,000006	0,000363	0,002276	0,025025	0,100875	0,004132	0,850926	0,263164	0,000007	0,000209	0,000938	0,002630	0,021724	0,002630	0,259329	0,207918	0,000005	0,000018	0,000145	0,000410	0,000200	0,000580	0,006439	0,015254
6	NC	VI	0,459733	0,415894	0,781293	0,673720	0,697742		0,996254	0,082886	0,000023	0,001254	0,006953	0,061298	0,207091	0,012037	0,841006	0,462758	0,000028	0,000745	0,003054	0,007945	0,054046	0,007945	0,457224	0,381055	0,000020	0,000073	0,000528	0,001407	0,000715	0,001948	0,018056	0,039390
7	NC	VII	0,462570	0,418569	0,777694	0,677144	0,694277	0,996254		0,082086	0,000023	0,001272	0,007043	0,061925	0,208755	0,012187	0,837338	0,465606	0,000028	0,000756	0,003096	0,008047	0,054609	0,008047	0,460052	0,383590	0,000021	0,000074	0,000536	0,001427	0,000726	0,001976	0,018272	0,039819
8	NC	VIII	0,014781	0,012165	0,142996	0,032576	0,174968	0,082886	0,082086		0,000000	0,000003	0,000024	0,000502	0,003462	0,000051	0,123613	0,014973	0,000000	0,000002	0,000009	0,000029	0,000416	0,000029	0,014623	0,010299	0,000000	0,000000	0,000001	0,000003	0,000001	0,000005	0,000088	0,000262
9	4 months	I	0,000301	0,000385	0,000008	0,000102	0,000006	0,000023	0,000023	0,000000		0,238079	0,080157	0,009803	0,001619	0,051724	0,000011	0,000296	0,959996	0,309604	0,141738	0,072392	0,011424	0,072392	0,000305	0,000473	0,973864	0,744838	0,362930	0,223743	0,315676	0,186329	0,036148	0,016436
10	4 months	II	0,010614	0,012928	0,000521	0,004394	0,000363	0,001254	0,001272	0,000003	0,238079		0,559232	0,146084	0,039520	0,431555	0,000667	0,010473	0,258297	0,868096	0,767595	0,527121	0,162312	0,527121	0,010732	0,015218	0,225507	0,390778	0,784514	0,970116	0,858020	0,885229	0,346010	0,207594
11	4 months	III	0,044932	0,053135	0,003158	0,021012	0,002276	0,006953	0,007043	0,000024	0,080157	0,559232		0,379823	0,134783	0,838627	0,003949	0,044424	0,088878	0,453733	0,772544	0,961210	0,411540	0,961210	0,045358	0,060995	0,074854	0,151630	0,392098	0,584612	0,446094	0,659882	0,718320	0,495174
12	4 months	IV	0,249935	0,281576	0,032662	0,143291	0,025025	0,061298	0,061925	0,000502	0,009803	0,146084	0,379823		0,530732	0,499045	0,039110	0,247912	0,011204	0,106300	0,244501	0,406537	0,954128	0,406537	0,251627	0,310260	0,008976	0,022666	0,085612	0,156501	0,103635	0,189397	0,603447	0,843428
13	4 months	V	0,597442	0,650198	0,125279	0,397656	0,100875	0,207091	0,208755	0,003462	0,001619	0,039520	0,134783	0,530732		0,194819	0,144865	0,593983	0,001885	0,026683	0,075790	0,147590	0,493898	0,147590	0,600326	0,696017	0,001464	0,004240	0,020490	0,043067	0,025865	0,054738	0,253462	0,410393
14	4 months	VI	0,070271	0,082198	0,005659	0,034388	0,004132	0,012037	0,012187	0,000051	0,051724	0,431555	0,838627	0,499045	0,194819		0,007011	0,069528	0,057775	0,341527	0,622467	0,876795	0,536080	0,876795	0,070895	0,093487	0,048071	0,102698	0,290371	0,453659	0,335127	0,520193	0,875094	0,631771
15	4 months	VII	0,348110	0,311426	0,938562	0,534575	0,850926	0,841006	0,837338	0,123613	0,000011	0,000667	0,003949	0,039110	0,144865	0,007011		0,350662	0,000013	0,000390	0,001678	0,004540	0,034203	0,004540	0,345997	0,282667	0,000010	0,000036	0,000273	0,000752	0,000374	0,001052	0,010731	0,024446
16	4 months	VIII	0,996006	0,936386	0,312719	0,753211	0,263164	0,462758	0,465606	0,014973	0,000296	0,010473	0,044424	0,247912	0,593983	0,069528	0,350662		0,000349	0,006670	0,022377	0,049572	0,225505	0,049572	0,992682	0,886512	0,000265	0,000849	0,004938	0,011567	0,006438	0,015282	0,096258	0,177224
17	5 months	I	0,000355	0,000453	0,000010	0,000121	0,000007	0,000028	0,000028	0,000000	0,959996	0,258297	0,088878	0,011204	0,001885	0,057775	0,000013	0,000349		0,333808	0,155489	0,080411	0,013033	0,080411	0,000360	0,000556	0,933916	0,783049	0,389735	0,243085	0,340191	0,203251	0,040603	0,018666
18	5 months	II	0,006764	0,008303	0,000302	0,002712	0,000209	0,000745	0,000756	0,000002	0,309604	0,868096	0,453733	0,106300	0,026683	0,341527	0,000390	0,006670	0,333808		0,644560	0,425161	0,118947	0,425161	0,006842	0,009839	0,294452	0,488127	0,914455	0,838724	0,989770	0,756307	0,268551	0,154790
19	5 months	III	0,022655	0,027195	0,001325	0,009958	0,000938	0,003054	0,003096	0,000009	0,141738	0,767595	0,772544	0,244501	0,075790	0,622467	0,001678	0,022377	0,155489	0,644560		0,735644	0,268227	0,735644	0,022889	0,031618	0,133286	0,250016	0,569748	0,796336	0,635413	0,879776	0,516365	0,332591
20	5 months	IV	0,050130	0,059129	0,003638	0,023695	0,002630	0,007945	0,008047	0,000029	0,072392	0,527121	0,961210	0,406537	0,147590	0,876795	0,004540	0,049572	0,080411	0,425161	0,735644		0,439573	1,000000	0,050598	0,067727	0,067525	0,138545	0,365989	0,551771	0,417811	0,625134	0,754938	0,526267
21	5 months	V	0,227395	0,257010	0,028477	0,128592	0,021724	0,054046	0,054609	0,000416	0,011424	0,162312	0,411540	0,954128	0,493898	0,536080	0,034203	0,225505	0,013033	0,118947	0,268227	0,439573		0,439573	0,228975	0,283960	0,010473	0,026093	0,096233	0,173607	0,116028	0,209134	0,644017	0,888656
22	5 months	VI	0,050130	0,059129	0,003638	0,023695	0,002630	0,007945	0,008047	0,000029	0,072392	0,527121	0,961210	0,406537	0,147590	0,876795	0,004540	0,049572	0,080411	0,425161	0,735644	1,000000	0,439573		0,050598	0,067727	0,067525	0,138545	0,365989	0,551771	0,417811	0,625134	0,754938	0,526267
23	5 months	VII	0,996677	0,943682	0,308396	0,746265	0,259329	0,457224	0,460052	0,014623	0,000305	0,010732	0,045358	0,251627	0,600326	0,070895	0,345997	0,992682	0,000360	0,006842	0,022889	0,050598	0,228975	0,050598		0,893759	0,000274	0,000874	0,005067	0,011850	0,006603	0,015646	0,098047	0,180124
24	5 months	VIII	0,890466	0,949835	0,250016	0,647753	0,207918	0,381055	0,383590	0,010299	0,000473	0,015218	0,060995	0,310260	0,696017	0,093487	0,282667	0,886512	0,000556	0,009839	0,031618	0,067727	0,283960	0,067727	0,893759		0,000425	0,001325	0,007352	0,016750	0,009507	0,021909	0,127320	0,226528
25	6 months	I	0,000270	0,000346	0,000007	0,000091	0,000005	0,000020	0,000021	0,000000	0,973864	0,225507	0,074854	0,008976	0,001464	0,048071	0,000010	0,000265	0,933916	0,294452	0,133286	0,067525	0,010473	0,067525	0,000274	0,000425		0,720216	0,346076	0,211732	0,300320	0,175866	0,033475	0,015113
26	6 months	II	0,000862	0,001090	0,000027	0,000307	0,000018	0,000073	0,000074	0,000000	0,744838	0,390778	0,151630	0,022666	0,004240	0,102698	0,000036	0,000849	0,783049	0,488127	0,250016	0,138545	0,026093	0,138545	0,000874	0,001325	0,720216		0,557618	0,370609	0,496163	0,316780	0,074455	0,036413

27	6 months	III	0,005008	0,006178	0,000211	0,001968	0,000145	0,000528	0,000536	0,000001	0,362930	0,784514	0,392098	0,085612	0,020490	0,290371	0,000273	0,004938	0,389735	0,914455	0,569748	0,365989	0,096233	0,365989	0,005067	0,007352	0,346076	0,557618		0,755902	0,924632	0,676246	0,225458	0,126632
28	6 months	IV	0,011721	0,014251	0,000588	0,004888	0,000410	0,001407	0,001427	0,000003	0,223743	0,970116	0,584612	0,156501	0,043067	0,453659	0,000752	0,011567	0,243085	0,838724	0,796336	0,551771	0,173607	0,551771	0,011850	0,016750	0,211732	0,370609	0,755902		0,828721	0,914878	0,365323	0,221171
29	6 months	V	0,006528	0,008018	0,000290	0,002611	0,000200	0,000715	0,000726	0,000001	0,315676	0,858020	0,446094	0,103635	0,025865	0,335127	0,000374	0,006438	0,340191	0,989770	0,635413	0,417811	0,116028	0,417811	0,006603	0,009507	0,300320	0,496163	0,924632	0,828721		0,746588	0,263122	0,151192
30	6 months	VI	0,015480	0,018723	0,000826	0,006596	0,000580	0,001948	0,001976	0,000005	0,186329	0,885229	0,659882	0,189397	0,054738	0,520193	0,001052	0,015282	0,203251	0,756307	0,879776	0,625134	0,209134	0,625134	0,015646	0,021909	0,175866	0,316780	0,676246	0,914878	0,746588		0,424123	0,263463
31	6 months	VII	0,097231	0,112761	0,008727	0,049320	0,006439	0,018056	0,018272	0,000088	0,036148	0,346010	0,718320	0,603447	0,253462	0,875094	0,010731	0,096258	0,040603	0,268551	0,516365	0,754938	0,644017	0,754938	0,098047	0,127320	0,033475	0,074455	0,225458	0,365323	0,263122	0,424123		0,747203
32	6 months	VIII	0,178803	0,203681	0,020205	0,097804	0,015254	0,039390	0,039819	0,000262	0,016436	0,207594	0,495174	0,843428	0,410393	0,631771	0,024446	0,177224	0,018666	0,154790	0,332591	0,526267	0,888656	0,526267	0,180124	0,226528	0,015113	0,036413	0,126632	0,221171	0,151192	0,263463	0,747203	

Table S7: Post-hoc comparison of laser treatment methods

Cell No.	LSD test; variable Drug content (%) (Spreadsheet1 in statistics) Probabilities for Post Hoc Tests Error: Between MS = 21,272, df = 72,000						
	Sample	{1}	{2}	{3}	{4}	{5}	{6}
1	NC		0,000000	0,000000	0,000000	0,000000	0,000000
2	PC	0,000000		0,000008	0,010442	0,000226	0,005867
3	Ti:Sa	0,000000	0,000008		0,100535	0,683692	0,150365
4	Nd:YAG	0,000000	0,010442	0,100535		0,255906	0,848595
5	KrF	0,000000	0,000226	0,683692	0,255906		0,343462
6	ArF	0,000000	0,005867	0,150365	0,848595	0,343462	

Table S8: Post hoc comparison of coating formulations against laser treatment

Cell No.	LSD test; variable Drug content (%) (Spreadsheet1 in statistics) Probabilities for Post Hoc Tests Error: Between MS = 21,272, df = 72,000								
	Formulation	{1}	{2}	{3}	{4}	{5}	{6}	{7}	{8}
1	I		0,141459	0,008583	0,001788	0,000065	0,000001	0,000004	0,000000
2	II	0,141459		0,228115	0,083099	0,007390	0,000158	0,000812	0,000042
3	III	0,008583	0,228115		0,589612	0,127642	0,007068	0,025514	0,002375
4	IV	0,001788	0,083099	0,589612		0,320940	0,028790	0,086293	0,011050

5	V	0,000065	0,007390	0,127642	0,320940		0,222075	0,461903	0,111966
6	VI	0,000001	0,000158	0,007068	0,028790	0,222075		0,624208	0,706960
7	VII	0,000004	0,000812	0,025514	0,086293	0,461903	0,624208		0,387491
8	VIII	0,000000	0,000042	0,002375	0,011050	0,111966	0,706960	0,387491	

Table S9: Post-hoc comparison of laser treatment results

LSD test; variable Drug content (%) (Spreadsheet1 in statistics)
Probabilities for Post Hoc Tests
Error: Between MS = 21,272, df = 72,000

Cell No.	Sample	Formulation	{1}	{2}	{3}	{4}	{5}	{6}	{7}	{8}	{9}	{10}	{11}	{12}	{13}	{14}	{15}	{16}
1	NC	I		0,934521	0,265343	0,725748	0,217246	0,416899	0,419844	0,007519	0,000067	0,000264	0,002099	0,005721	0,002870	0,007939	0,068756	0,139990
2	NC	II	0,934521		0,232137	0,665149	0,188624	0,371670	0,374414	0,005977	0,000090	0,000348	0,002689	0,007202	0,003657	0,009933	0,081764	0,162888
3	NC	III	0,265343	0,232137		0,443567	0,903079	0,760434	0,756513	0,107899	0,000001	0,000005	0,000050	0,000168	0,000073	0,000251	0,004041	0,010865
4	NC	IV	0,725748	0,665149	0,443567		0,375028	0,643793	0,647486	0,019060	0,000019	0,000078	0,000698	0,002040	0,000974	0,002905	0,031029	0,069230
5	NC	V	0,217246	0,188624	0,903079	0,375028		0,669730	0,665985	0,136501	0,000001	0,000003	0,000032	0,000110	0,000047	0,000165	0,002824	0,007803
6	NC	VI	0,416899	0,371670	0,760434	0,643793	0,669730		0,995886	0,057038	0,000003	0,000014	0,000148	0,000470	0,000211	0,000689	0,009518	0,023827
7	NC	VII	0,419844	0,374414	0,756513	0,647486	0,665985	0,995886		0,056394	0,000003	0,000015	0,000150	0,000478	0,000215	0,000701	0,009652	0,024133
8	NC	VIII	0,007519	0,005977	0,107899	0,019060	0,136501	0,057038	0,056394		0,000000	0,000000	0,000000	0,000000	0,000000	0,000001	0,000018	0,000065
9	PC	I	0,000067	0,000090	0,000001	0,000019	0,000001	0,000003	0,000003	0,000000		0,694059	0,300872	0,170393	0,255459	0,137317	0,019679	0,007718
10	PC	II	0,000264	0,000348	0,000005	0,000078	0,000003	0,000014	0,000015	0,000000	0,694059		0,519611	0,325559	0,454871	0,271695	0,050302	0,021725
11	PC	III	0,002099	0,002689	0,000050	0,000698	0,000032	0,000148	0,000150	0,000000	0,300872	0,519611		0,732797	0,917252	0,646517	0,183282	0,093631
12	PC	IV	0,005721	0,007202	0,000168	0,002040	0,000110	0,000470	0,000478	0,000000	0,170393	0,325559	0,732797		0,812192	0,906550	0,320221	0,179244
13	PC	V	0,002870	0,003657	0,000073	0,000974	0,000047	0,000211	0,000215	0,000000	0,255459	0,454871	0,917252	0,812192		0,722675	0,219228	0,115145
14	PC	VI	0,007939	0,009933	0,000251	0,002905	0,000165	0,000689	0,000701	0,000001	0,137317	0,271695	0,646517	0,906550	0,722675		0,380118	0,219558
15	PC	VII	0,068756	0,081764	0,004041	0,031029	0,002824	0,009518	0,009652	0,000018	0,019679	0,050302	0,183282	0,320221	0,219228	0,380118		0,723343
16	PC	VIII	0,139990	0,162888	0,010865	0,069230	0,007803	0,023827	0,024133	0,000065	0,007718	0,021725	0,093631	0,179244	0,115145	0,219558	0,723343	
17	Ti:Sa	I	0,007751	0,009702	0,000243	0,002830	0,000160	0,000670	0,000681	0,000001	0,139581	0,275460	0,652767	0,913448	0,729196	0,993056	0,375450	0,216358
18	Ti:Sa	II	0,040395	0,048733	0,001984	0,017172	0,001363	0,004891	0,004963	0,000007	0,035242	0,084190	0,273284	0,449004	0,320801	0,521991	0,811260	0,553668
19	Ti:Sa	III	0,123587	0,144389	0,009101	0,060079	0,006503	0,020233	0,020497	0,000051	0,009237	0,025551	0,106794	0,201019	0,130662	0,244799	0,772993	0,947694

Cell No.	{17}	{18}	{19}	{20}	{21}	{22}	{23}	{24}	{25}	{26}	{27}	{28}	{29}	{30}	{31}	{32}
1	0,007751	0,040395	0,123587	0,397531	0,477628	0,809567	0,373445	0,546845	0,007192	0,028094	0,052389	0,031577	0,092137	0,562507	0,529533	0,204160
2	0,009702	0,048733	0,144389	0,444608	0,529776	0,873779	0,418804	0,602641	0,008804	0,033504	0,061598	0,037562	0,106787	0,612954	0,578552	0,231126
3	0,000243	0,001984	0,009101	0,052254	0,070421	0,176673	0,047297	0,088285	0,000331	0,001781	0,003967	0,002066	0,008381	0,117147	0,106236	0,025231
4	0,002830	0,017172	0,060079	0,232815	0,289982	0,554353	0,216158	0,341511	0,002916	0,012691	0,025106	0,014416	0,046898	0,372816	0,346929	0,114781
5	0,000160	0,001363	0,006503	0,039607	0,054029	0,141470	0,035710	0,068395	0,000229	0,001271	0,002879	0,001479	0,006194	0,094360	0,085225	0,019234
6	0,000670	0,004891	0,020233	0,099735	0,130308	0,293417	0,091178	0,159380	0,000810	0,004026	0,008570	0,004633	0,017270	0,193644	0,177415	0,047992
7	0,000681	0,004963	0,020497	0,100771	0,131592	0,295765	0,092140	0,160883	0,000822	0,004080	0,008679	0,004695	0,017475	0,195202	0,178874	0,048494
8	0,000001	0,000007	0,000051	0,000578	0,000899	0,003789	0,000500	0,001266	0,000002	0,000012	0,000033	0,000015	0,000085	0,003278	0,002828	0,000365
9	0,139581	0,035242	0,009237	0,001165	0,000754	0,000156	0,001339	0,000530	0,311255	0,126680	0,073857	0,115325	0,041135	0,002016	0,002345	0,014507
10	0,275460	0,084190	0,025551	0,003846	0,002566	0,000588	0,004374	0,001849	0,507208	0,237188	0,148455	0,218737	0,088593	0,005676	0,006530	0,034767
11	0,652767	0,273284	0,106794	0,022048	0,015579	0,004291	0,024607	0,011733	0,930344	0,541700	0,380781	0,510137	0,254998	0,026037	0,029382	0,120104
12	0,913448	0,449004	0,201019	0,049552	0,036186	0,011104	0,054709	0,027959	0,827400	0,760023	0,566868	0,723354	0,403194	0,053150	0,059325	0,209435
13	0,729196	0,320801	0,130662	0,028455	0,020306	0,005776	0,031655	0,015410	0,995598	0,604722	0,432925	0,571354	0,295323	0,032579	0,036643	0,143305
14	0,993056	0,521991	0,244799	0,064198	0,047432	0,015126	0,070610	0,036982	0,746727	0,841074	0,639809	0,803300	0,464412	0,066874	0,074357	0,249445
15	0,375450	0,811260	0,772993	0,322309	0,260626	0,112693	0,344247	0,218149	0,268952	0,557952	0,749954	0,590948	0,956804	0,287836	0,310749	0,711577
16	0,216358	0,553668	0,947694	0,523434	0,438855	0,215192	0,552587	0,378064	0,156489	0,367699	0,525637	0,393883	0,710825	0,453943	0,484338	0,957609
17		0,516374	0,241336	0,062999	0,046506	0,014788	0,069311	0,036236	0,752623	0,834995	0,634266	0,797289	0,459699	0,065763	0,073143	0,246304
18	0,516374		0,598274	0,220382	0,173855	0,069078	0,237273	0,142703	0,371280	0,709315	0,916236	0,745746	0,873312	0,202850	0,220766	0,559990
19	0,241336	0,598274		0,481834	0,401309	0,192262	0,509753	0,343893	0,173996	0,399463	0,564461	0,427000	0,754932	0,419572	0,448633	0,910960

Cell No.	{33}	{34}	{35}	{36}	{37}	{38}	{39}	{40}	{41}	{42}	{43}	{44}	{45}	{46}	{47}	{48}
1	0,000334	0,060828	0,085441	0,289288	0,525192	0,810105	0,755066	0,529533	0,000928	0,009174	0,009747	0,066815	0,404114	0,754137	0,393662	0,456005
2	0,000426	0,071261	0,099226	0,323643	0,574012	0,753735	0,811463	0,578552	0,001171	0,011183	0,011868	0,078091	0,446479	0,699066	0,435379	0,501367
3	0,000009	0,004824	0,007573	0,041884	0,104838	0,448017	0,191938	0,106236	0,000030	0,000444	0,000478	0,005459	0,069400	0,492609	0,066654	0,083762
4	0,000114	0,029598	0,043122	0,171081	0,343551	0,941371	0,531901	0,346929	0,000332	0,003785	0,004040	0,032833	0,252228	0,999528	0,244614	0,290674
5	0,000006	0,003517	0,005582	0,032456	0,084058	0,386000	0,158037	0,085225	0,000020	0,000309	0,000333	0,003992	0,054754	0,426926	0,052508	0,066561
6	0,000026	0,010298	0,015710	0,076390	0,175322	0,626230	0,300221	0,177415	0,000080	0,001074	0,001153	0,011566	0,120904	0,678700	0,116565	0,143289
7	0,000026	0,010427	0,015898	0,077134	0,176767	0,629495	0,302354	0,178874	0,000081	0,001090	0,001170	0,011708	0,121986	0,682075	0,117616	0,144526
8	0,000000	0,000042	0,000074	0,000734	0,002772	0,029639	0,007060	0,002828	0,000000	0,000002	0,000002	0,000049	0,001508	0,035262	0,001423	0,001986
9	0,985552	0,063897	0,044761	0,008202	0,002393	0,000138	0,000874	0,002345	0,740930	0,271265	0,261780	0,058133	0,004319	0,000107	0,004560	0,003327
10	0,738548	0,130740	0,095490	0,020693	0,006653	0,000455	0,002601	0,006530	0,983032	0,452459	0,439220	0,120305	0,011487	0,000357	0,012076	0,009028
11	0,363834	0,345382	0,270483	0,078170	0,029857	0,002798	0,013200	0,029382	0,550293	0,860303	0,842806	0,323878	0,047621	0,002247	0,049687	0,038788
12	0,226287	0,522094	0,424079	0,143155	0,060197	0,006759	0,028541	0,059325	0,367592	0,896984	0,914670	0,494430	0,091829	0,005505	0,095408	0,076310
13	0,317247	0,394528	0,312448	0,094664	0,037220	0,003681	0,016804	0,036643	0,490289	0,933792	0,916060	0,371083	0,058574	0,002969	0,061037	0,047998
14	0,189100	0,592305	0,487071	0,173415	0,075411	0,009029	0,036647	0,074357	0,314896	0,814645	0,832010	0,562782	0,113257	0,007389	0,117497	0,094778
15	0,037833	0,801778	0,927504	0,560335	0,313910	0,062270	0,184635	0,310749	0,075741	0,308714	0,319330	0,835453	0,418682	0,053037	0,429530	0,369469
16	0,017436	0,570561	0,683631	0,790080	0,488495	0,119426	0,310393	0,484338	0,037470	0,183479	0,190806	0,600276	0,621745	0,103476	0,635082	0,560199
17	0,191688	0,586952	0,482231	0,171016	0,074183	0,008839	0,035984	0,073143	0,318620	0,820689	0,838085	0,557559	0,111541	0,007232	0,115730	0,093293
18	0,061266	0,970111	0,902394	0,426666	0,223253	0,038498	0,124609	0,220766	0,116767	0,420154	0,433054	0,995302	0,307506	0,032422	0,316425	0,267494
19	0,020233	0,610945	0,727147	0,745309	0,452615	0,106454	0,283536	0,448633	0,042924	0,203205	0,211110	0,641590	0,581025	0,091941	0,593959	0,521527

20	Ti:Sa	IV	0,3 975 31	0,4 446 08	0,0 522 54	0,2 328 15	0,0 396 07	0,0 997 35	0,1 007 71	0,0 005 78	0,0 011 65	0,0 038 46	0,0 220 48	0,0 495 52	0,0 284 55	0,0 641 98	0,3 223 09	0,5 234 34	0,0 629 99	0,2 203 82	0,4 818 34		0,8 912 10	0,5 442 86	0,9 646 26	0,8 065 75	0,0 486 75	0,1 432 38	0,2 297 47	0,1 566 88	0,3 474 57	0,8 580 99	0,8 973 42	0,6 045 48	0,0 036 31	0,2 566 86	0,3 290 62	0,7 602 60	0,9 026 22	0,3 194 94	0,6 554 30	0,8 973 42	0,0 087 96	0,0 592 66	0,0 622 24	0,2 750 70	0,9 380 58	0,2 856 79	0,9 230 85	0,9 906 45
21	Ti:Sa	V	0,4 776 28	0,5 297 76	0,0 704 21	0,2 899 82	0,0 540 29	0,1 303 08	0,1 315 92	0,0 008 99	0,0 007 54	0,0 025 66	0,0 155 79	0,0 361 86	0,0 203 06	0,0 474 32	0,2 606 26	0,4 388 55	0,0 465 06	0,1 738 55	0,4 013 09	0,8 912 10		0,6 383 70	0,8 562 81	0,9 139 08	0,0 367 44	0,1 133 66	0,1 863 91	0,1 245 68	0,2 888 72	0,9 549 66	0,9 946 65	0,5 223 14	0,0 025 27	0,2 095 57	0,2 726 52	0,6 691 55	0,9 999 89	0,3 819 69	0,7 458 76	0,9 946 65	0,0 062 68	0,0 450 91	0,0 474 36	0,2 254 69	0,8 414 60	0,3 438 43	0,8 267 61	0,9 119 24
22	Ti:Sa	VI	0,8 095 67	0,8 737 79	0,1 766 73	0,5 543 53	0,1 414 70	0,2 934 17	0,2 957 65	0,0 037 89	0,0 001 56	0,0 005 88	0,0 042 91	0,0 111 04	0,0 057 76	0,0 151 26	0,1 126 93	0,2 151 92	0,0 147 88	0,0 690 78	0,1 922 62	0,5 442 86	0,6 383 70		0,5 153 63	0,7 173 07	0,0 128 89	0,0 465 86	0,0 833 06	0,0 519 69	0,1 404 26	0,7 158 25	0,6 790 55	0,2 904 00	0,0 006 79	0,0 956 90	0,1 309 97	0,3 974 71	0,6 741 84	0,6 486 80	0,9 231 38	0,6 790 55	0,0 018 20	0,0 162 34	0,0 171 92	0,1 043 82	0,5 353 18	0,5 972 44	0,5 230 39	0,5 955 72
23	Ti:Sa	VII	0,3 734 45	0,4 188 04	0,0 472 97	0,2 161 58	0,0 357 10	0,0 911 78	0,0 921 40	0,0 005 00	0,0 013 39	0,0 043 74	0,0 246 07	0,0 547 09	0,0 316 55	0,0 706 10	0,3 442 47	0,5 525 87	0,0 693 11	0,2 372 73	0,5 097 53	0,9 646 26	0,8 562 81	0,5 153 63		0,7 724 57	0,0 531 98	0,1 541 49	0,2 452 68	0,1 683 78	0,3 680 05	0,8 270 82	0,8 660 50	0,6 324 32	0,0 040 77	0,2 734 71	0,3 489 05	0,7 906 20	0,8 712 99	0,3 007 90	0,6 271 03	0,8 660 50	0,0 097 96	0,0 646 07	0,0 677 88	0,2 926 76	0,9 696 51	0,2 683 69	0,9 546 38	0,9 590 13
24	Ti:Sa	VIII	0,5 468 45	0,6 026 41	0,0 882 85	0,3 415 11	0,0 683 95	0,1 593 80	0,1 608 83	0,0 012 66	0,0 005 30	0,0 018 49	0,0 117 33	0,0 279 59	0,0 154 10	0,0 369 82	0,2 181 49	0,3 780 64	0,0 362 36	0,1 427 03	0,3 438 93	0,8 065 75	0,9 139 08	0,7 173 07	0,7 724 57		0,0 291 98	0,0 934 86	0,1 567 31	0,1 030 85	0,2 476 55	0,9 679 11	0,9 282 77	0,4 617 27	0,0 018 85	0,1 770 85	0,2 331 20	0,6 004 32	0,9 229 77	0,4 364 14	0,8 200 68	0,9 282 77	0,0 047 63	0,0 360 51	0,0 379 85	0,1 911 38	0,7 667 22	0,3 949 28	0,7 523 93	0,8 357 86
25	Nd:YAG	I	0,0 071 92	0,0 088 04	0,0 003 31	0,0 029 16	0,0 002 29	0,0 008 10	0,0 008 22	0,0 000 02	0,3 112 55	0,5 072 08	0,9 303 44	0,8 274 00	0,9 955 98	0,7 467 27	0,2 689 52	0,1 564 89	0,7 526 23	0,3 712 80	0,1 739 96	0,0 486 75	0,0 367 44	0,0 128 89	0,0 531 98	0,0 291 98		0,6 329 21	0,4 708 27	0,6 017 71	0,3 365 88	0,0 498 83	0,0 552 04	0,1 793 79	0,3 635 99	0,4 338 73	0,3 536 02	0,1 252 64	0,0 559 52	0,0 076 19	0,0 281 95	0,0 552 04	0,5 320 36	0,9 355 43	0,9 193 51	0,4 111 37	0,0 827 38	0,0 063 29	0,0 857 33	0,0 696 75
26	Nd:YAG	II	0,0 280 94	0,0 335 04	0,0 017 81	0,0 126 91	0,0 012 71	0,0 040 26	0,0 040 80	0,0 000 12	0,1 266 80	0,2 371 88	0,5 417 00	0,7 600 23	0,6 047 22	0,8 410 74	0,5 579 52	0,3 676 99	0,8 349 95	0,7 093 15	0,3 994 63	0,1 432 38	0,1 133 66	0,0 465 86	0,1 541 49	0,0 934 86	0,6 329 21		0,8 069 26	0,9 646 24	0,6 272 26	0,1 341 90	0,1 461 24	0,3 838 34	0,1 676 09	0,7 594 85	0,6 511 99	0,2 875 55	0,1 477 85	0,0 264 50	0,0 826 39	0,1 461 24	0,2 717 23	0,6 914 35	0,7 065 01	0,7 295 74	0,2 047 05	0,0 224 48	0,2 108 09	0,1 775 12
27	Nd:YAG	III	0,0 523 89	0,0 615 98	0,0 039 67	0,0 251 06	0,0 028 79	0,0 085 70	0,0 086 79	0,0 000 33	0,0 738 57	0,1 484 55	0,3 807 81	0,5 668 68	0,4 329 25	0,6 398 09	0,7 499 54	0,5 256 37	0,6 342 66	0,9 162 36	0,5 644 61	0,2 297 47	0,1 863 91	0,0 833 06	0,2 452 68	0,1 567 31	0,4 708 27	0,8 069 26		0,8 414 29	0,8 091 36	0,2 083 34	0,2 249 64	0,5 300 98	0,1 055 17	0,9 507 13	0,8 352 73	0,4 114 57	0,2 272 65	0,0 470 16	0,1 342 01	0,2 249 64	0,1 803 24	0,5 217 49	0,5 350 23	0,9 192 43	0,3 043 38	0,0 403 67	0,3 124 17	0,2 679 26
28	Nd:YAG	IV	0,0 315 77	0,0 375 62	0,0 020 66	0,0 144 16	0,0 014 79	0,0 046 33	0,0 046 95	0,0 000 15	0,1 153 25	0,2 187 37	0,5 101 37	0,7 233 54	0,5 713 54	0,8 033 00	0,5 909 48	0,3 938 83	0,7 972 89	0,7 457 46	0,4 270 00	0,1 566 88	0,1 245 68	0,0 519 69	0,1 683 78	0,1 030 85	0,6 017 71	0,9 646 24	0,8 414 29		0,6 589 36	0,1 458 27	0,1 585 55	0,4 083 30	0,1 546 27	0,7 934 42	0,6 833 98	0,3 078 94	0,1 603 24	0,0 294 53	0,0 905 38	0,1 585 55	0,2 530 91	0,6 590 84	0,6 738 74	0,7 631 18	0,2 207 17	0,0 250 48	0,2 271 66	0,1 919 23
29	Nd:YAG	V	0,0 921 37	0,1 067 87	0,0 083 81	0,0 468 98	0,0 061 94	0,0 172 70	0,0 174 75	0,0 000 85	0,0 411 35	0,0 885 93	0,2 549 98	0,4 031 94	0,2 953 23	0,4 644 12	0,9 568 04	0,7 108 25	0,4 596 99	0,8 733 12	0,7 549 32	0,3 474 57	0,2 888 72	0,1 404 26	0,3 680 05	0,2 476 55	0,3 365 88	0,6 272 26	0,8 091 36	0,6 589 36		0,3 078 03	0,3 296 27	0,6 988 09	0,0 639 17	0,8 573 43	0,9 731 84	0,5 612 44	0,3 326 28	0,0 795 59	0,2 073 36	0,3 296 27	0,1 150 21	0,3 784 42	0,3 894 86	0,8 885 11	0,4 308 88	0,0 691 15	0,4 409 53	0,3 849 93
30	Nd:YAG	VI	0,5 625 07	0,6 129 54	0,1 171 47	0,3 728 16	0,0 943 60	0,1 936 44	0,1 952 02	0,0 032 78	0,0 020 16	0,0 056 76	0,0 260 37	0,0 531 50	0,0 325 79	0,0 668 74	0,2 878 36	0,4 539 43	0,0 657 63	0,2 028 50	0,4 195 72	0,8 580 99	0,9 549 66	0,7 158 25	0,8 270 82	0,9 679 11	0,0 498 83	0,1 341 90	0,2 083 34	0,1 458 27	0,3 078 03		0,9 637 50	0,5 250 83	0,0 048 22	0,2 311 83	0,2 923 03	0,6 587 95	0,9 588 96	0,4 549 29	0,8 069 60	0,9 637 50	0,0 106 48	0,0 596 79	0,0 623 90	0,2 467 38	0,8 148 83	0,4 159 89	0,8 015 68	0,8 787 78
31	Nd:YAG	VII	0,5 295 33	0,5 785 52	0,1 062 36	0,3 469 29	0,0 852 25	0,1 774 15	0,1 788 74	0,0 028 28	0,0 023 45	0,0 065 30	0,0 293 82	0,0 593 25	0,0 366 43	0,0 743 57	0,3 107 49	0,4 843 38	0,0 731 43	0,2 207 66	0,4 486 33	0,8 973 42	0,9 946 65	0,6 790 55	0,8 660 50	0,9 282 77	0,0 552 04	0,1 461 24	0,2 249 64	0,1 585 55	0,3 296 27	0,9 637 50		0,5 550 17	0,0 054 89	0,2 491 07	0,3 133 89	0,6 919 49	0,9 951 40	0,4 281 33	0,7 719 96	1,0 000 00	0,0 120 20	0,0 658 67	0,0 688 13	0,2 655 07	0,8 503 25	0,3 906 07	0,8 368 73	0,9 147 27
32	Nd:YAG	VIII	0,2 041 60	0,2 311 26	0,0 252 31	0,1 147 81	0,0 192 34	0,0 479 92	0,0 484 94	0,0 003 65	0,0 145 07	0,0 347 67	0,1 201 04	0,2 094 35	0,1 433 05	0,2 494 45	0,7 115 77	0,9 576 09	0,2 463 04	0,5 599 90	0,9 109 60	0,6 045 48	0,5 223 14	0,2 904 00	0,6 324 32	0,4 617 27	0,1 793 79	0,3 838 34	0,5 300 98	0,4 083 30	0,6 988 09	0,5 250 83	0,5 550 17		0,0 261 90	0,5 712 07	0,6 741 30	0,8 457 66	0,5 590 92	0,1 688 34	0,3 796 97	0,5 550 17	0,0 510 92	0,2 065 10	0,2 138 04	0,5 983 19	0,6 876 66	0,1 495 30	0,7 003 45	0,6 287 40
33	KrF	I	0,0 003 34	0,0 004 26	0,0 000 09	0,0 001 14	0,0 000 06	0,0 000 26	0,0 000 26	0,0 000 00	0,9 855 52	0,7 385 48	0,3 638 34	0,2 262 87	0,3 172 47	0,1 891 00	0,0 378 33	0,0 174 36	0,1 916 88	0,0 612 66	0,0 202 33	0,0 036 31	0,0 025 27	0,0 006 79	0,0 040 77	0,0 018 85	0,3 635 99	0,1 676 09	0,1 055 17	0,1 546 27	0,0 639 17	0,0 048 22	0,0 054 89	0,0 261 90		0,0 932 00	0,0 687 12	0,0 160 70	0,0 055 85	0,0 004 77	0,0 023 49	0,0 054 89	0,7 754 01	0,3 228 38	0,3 130 65	0,0 859 51	0,0 092 73	0,0 003 83	0,0 097 15	0,0 074 12
34	KrF	II	0,0 608 28	0,0 712 61	0,0 048 24	0,0 295 98	0,0 035 17	0,0 102 98	0,0 104 27	0,0 000 42	0,0 638 97	0,1 307 40	0,3 453 82	0,5 220 94	0,3 945 28	0,5 923 05	0,8 017 78	0,5 705 61	0,5 869 52	0,9 701 11	0,6 109 45	0,2 566 86	0,2 095 57	0,0 956 90	0,2 734 71	0,1 770 85	0,4 338 73	0,7 594 85	0,9 507 13	0,7 934 42	0,8 573 43	0,2 311 83	0,2 491 07	0,5 712 07	0,0 932 00		0,8 838 02	0,4 473 01	0,2 515 83	0,0 540 09	0,1 506 31	0,2 491 07	0,1 614 03	0,4 825 71	0,4 953 05	0,9 684 29	0,3 340 43	0,0 465 09	0,3 426 36	0,2 951 98
35	KrF	III	0,0 854 41	0,0 992 26	0,0 075 73	0,0 431 22	0,0 055 82	0,0 157 10	0,0 158 98	0,0 000 74	0,0 447 61	0,0 954 90	0,2 704 83	0,4 240 79	0,3 124 48	0,4 870 71	0,9 275 04	0,6 836 31	0,4 822 31	0,9 023 94	0,7 271 47	0,3 290 62	0,2 726 52	0,1 309 97	0,3 489 05	0,2 331 20	0,3 536 02	0,6 511 99	0,8 352 73	0,6 833 98	0,9 731 84	0,2 923 03	0,3 133 89	0,6 741 30	0,0 687 12	0,8 838 02		0,5 388 95	0,3 162 91	0,0 741 35	0,1 956 75	0,3 133 89	0,1 227 70	0,3 967 63	0,4 081 32	0,9 151 23	0,4 116 12	0,0 642 96	0,4 214 08	0,3 670 18
36	KrF	IV	0,2 892 88	0,3 236 43	0,0 418 84	0,1 710 81	0,0 324 56	0,0 763 90	0,0 771 34	0,0 007 34	0,0 082 02	0,0 206 93	0,0 781 70	0,1 431 55	0,0 946 64	0,1 734 15	0,5 603 35	0,7 900 80	0,1 710 16	0,4 266 66	0,7 453 09	0,7 602 60	0,6 691 55	0,3 974 71	0,7 906 20	0,6 004 32	0,1 252 64	0,2 875 55	0,4 114 57	0,3 078 94	0,5 612 44	0,6 587 95	0,6 919 49	0,8 457 66	0,0 160 70	0,4 473 01	0,5 388 95		0,6 964 40	0,2 361 25	0,4 932 47	0,6 919 49	0,0 326 03	0,1 459 35	0,1 515 44	0,4 711 74	0,8 354 86	0,2 111 89	0,8 489 33	0,7 723 80
37	KrF	V	0,5 251 92	0,5 740 12	0,1 048 38	0,3 435 51	0,0 840 58	0,1 753 22	0,1 767 67	0,0 027 72	0,0 023 93	0,0 066 53	0,0 298 57	0,0 601 97	0,0 372 20	0,0 754 11	0,3 139 10	0,4 884 95	0,0 741 83	0,2 232 53	0,4 526 15	0,9 026 22	0,9 999 89	0,6 741 84	0,8 712 99	0,9 229 77	0,0 559 52	0,1 477 85	0,2 272 65	0,1 603 24	0,3 326 28	0,9 588 96	0,9 951 40	0,5 590 92	0,0 055 85	0,2 515 83	0,3 162 91	0,6 964 40		0,4 246 15	0,7 673 45	0,9 951 40	0,0 122 16	0,0 667 36	0,0 697 14	0,2 680 96	0,8 551 00	0,3 872 80	0,8 416 32	0,9 195 60
38	KrF	VI	0,8 101 05	0,7 537 35	0,4 480 17	0,9 413 71	0,3 860 00	0,6 262 30	0,6 294 95	0,0 296 39	0,0 001 38	0,0 004 55	0,0 027 98	0,0 067 59	0,0 036 81	0,0 090 29	0,0 622 70	0,1 194 26	0,0 088 39	0,0 384 98	0,1 064 54	0,3 194 94	0,3 819 69	0,6 486 80	0,3 007 90	0,4 364 14	0,0 076 19	0,0 264 50	0,0 470 16	0,0 294 53	0,0 795 59	0,4 549 29	0,4 281 33	0,1 688 34	0,0 004 77	0,0 540 09	0,0 741 35	0,2 361 25	0,4 246 15		0,6 143 78	0,4 281 33	0,0 011 97	0,0 095 06	0,0 100 45	0,0 589 37	0,3 273 00	0,9 469 01	0,3 189 64	0,3 688 27
39	KrF	VII	0,7 550 66	0,8 114 63	0,1 919 38	0,5 319 01	0,1 580 37	0,3 002 21	0,3 023 54	0,0 070 60	0,0 008 74	0,0 026 01	0,0 132 00	0,0 285 41	0,0 168 04	0,0 366 47	0,1 846 35	0,3 103 93	0,0 359 84	0,1 246 09	0,2 835 36	0,6 554 30	0,7 458 76	0,9 231 38	0,6 271 03	0,8 200 68	0,0 281 95	0,0 826 39	0,1 342 01	0,0 905 38	0,2 073 36	0,8 069 60	0,7 719 96	0,3 796 97	0,0 023 49	0,1 506 31	0,1 956 75	0,4 932 47	0,7 673 45	0,6 143 78		0,7 719 96	0,0 054 20	0,0 342 15	0,0 358 99	0,1 619 48	0,6 325 11	0,5 685 19	0,6 203 53	0,6 915 79
40	KrF	VIII	0,5 295 33	0,5 785 52	0,1 062 36	0,3 469 29	0,0 852 25	0,1 774 15	0,1 788 74	0,0 028 28	0,0 023 45	0,0 065 30	0,0 293 82	0,0 593 25	0,0 366 43	0,0 743 57	0,3 107 49	0,4 843 38	0,0 731 43	0,2 207 66	0,4 486 33	0,8 973 42	0,9 946 65	0,6 790 55	0,8 660 50	0,9 282 77	0,0 552 04	0,1 461 24	0,2 249 64	0,1 585 55	0,3 296 27	0,9 637 50	1,0 000 00	0,5 550 17	0,0 054 89	0,2 491 07	0,3 133 89	0,6 919 49	0,9 951 40	0,4 281 33	0,7 719 96		0,0 120 20	0,0 658 67	0,0 688 13	0,2 655 07	0,8 503 25	0,3 906 07	0,8 368 73	0,9 147 27
41	ArF	I	0,0 009 28	0,0 011 71	0,0 000 30	0,0 003 32	0,0 000 20	0,0 000 80	0,0 000 81	0,0 000 00	0,7 409 30	0,9 830 32	0,5 502 93	0,3 675 92	0,4 902 89	0,3 148 96	0,0 757 41	0,0 374 70	0,3 186 20	0,1 167 67	0,0 429 24	0,0 087 96	0,0 062 68	0,0 018 20	0,0 097 96	0,0 047 63	0,5 320 36	0,2 717 23	0,1 803 24	0,2 530 91	0,1 150 21	0,0 106 48	0,0 120 20	0,0 510 92	0,7 754 01	0,1 614 03	0,1 227 70	0,0 326 03	0,0 122 16	0,0 011 97	0,0 054 20	0,0 120 20		0,4 805 60	0,4 680 39	0,1 501 17	0,0 195 93	0,0 009 70	0,0 204 60	0,0 159 07
42	ArF	II	0,0 091 74	0,0 111 83	0,0 004 44	0,0 037 85	0,0 003 09	0,0 010 74	0,0 010 90	0,0 000 02	0,2 712 65	0,4 524 59	0,8 603 03	0,8 969 84	0,9 337 92	0,8 146 45	0,3 087 14	0,1 834 79	0,8 206 89	0,4 201 54	0,2 032 05	0,0 592 66	0,0 450 91	0,0 162 34	0,0 646 07	0,0 360 51	0,9 355 43	0,6 914 35	0,5 217 49	0,6 590 84	0,3 784 42	0,0 596 79	0,0 658 67	0,2 065 10	0,3 228 38	0,4 825 71	0,3 967 63	0,1 459 35	0,0 667 36	0,0 095 06	0,0 342 15	0,0 658 67	0,4 805 60		0,9 837 40	0,4 583 68	0,0 976 12	0,0 079 25	0,1 010 41	0,0 826 05
43	ArF	III	0,0 097 47	0,0 118 68	0,0 004 78	0,0 040 40	0,0 003 33	0,0 011 53	0,0 011 70	0,0 000 02	0,2 617 80	0,4 392 20	0,8 428 06	0,9 146 70	0,9 160 60	0,8 320 10	0,3 193 30	0,1 908 06	0,8 380 85	0,4 330 54	0,2 111 10	0,0 622 24	0,0 474 36	0,0 171 92	0,0 677 88	0,0 379 85	0,9 193 51	0,7 065 01	0,5 350 23	0,6 738 74	0,3 894 86	0,0 623 90	0,0 688 13	0,2 138 04	0,3 130 65	0,4 953 05	0,4 081 32	0,1 515 44	0,0 697 14	0,0 100 45	0,0 358 99	0,0 688 13	0,4 680 39	0,9 837 40		0,4 707 43	0,1 016 87	0,0 083 81	0,1 052 31	0,0 861 60
44	ArF	IV	0,0 668 15	0,0 780 91	0,0 054 59	0,0 328 33	0,0 039 92	0,0 115 66	0,0 117 08	0,0 000 49	0,0 581 33	0,1 203 05	0,3 238 78	0,4 944 30	0,3 710 83	0,5 627 82	0,8 354 53	0,6 002 76	0,5 575 59	0,9 953 02	0,6 415 90	0,2 750 70	0,2 254 69	0,1 043 82	0,2 926 76	0,1 911 38	0,4 111 37	0,7 295 74	0,9 192 43	0,7 631 18	0,8 885 11	0,2 467 38	0,2 655 07	0,5 983 19	0,0 859 51	0,9 684 29	0,9 151 23	0,4 711 74	0,2 680 96	0,0 589 37	0,1 619 48	0,2 655 07	0,1 501 17	0,4 583 68	0,4 707 43		0,3 540 34	0,0 508 50	0,3 629 56	0,3 136 26
45	ArF	V	0,4 041 14	0,4 464 79	0,0 694 00	0,2 522 28	0,0 547 54	0,1 209 04	0,1 219 86	0,0 015 08	0,0 043 19	0,0 114 87	0,0 476 21	0,0 918 29	0,0 585 74	0,1 132 57	0,4 186 82	0,6 217 45	0,1 115 41	0,3 075 06	0,5 810 25	0,9 380 58	0,8 414 60	0,5 353 18	0,9 696 51	0,7 667 22	0,0 827 38	0,2 047 05	0,3 043 38	0,2 207 17	0,4 308 88	0,8 148 83	0,8 503 25	0,6 876 66	0,0 092 73	0,3 340 43	0,4 116 12	0,8 354 86	0,8 551 00	0,3 273 00	0,6 325 11	0,8 503 25	0,0 195 93	0,0 976 12	0,1 016 87	0,3 540 34		0,2 958 12	0,9 862 79	0,9 349 30
46	ArF	VI	0,7 541 37	0,6 990 66	0,4 926 09	0,9 995 28	0,4 269 26	0,6 787 00	0,6 820 75	0,0 352 62	0,0 001 07	0,0 003 57	0,0 022 47	0,0 055 05	0,0 029 69	0,0 073 89	0,0 530 37	0,1 034 76	0,0 072 32	0,0 324 22	0,0 919 41	0,2 856 79	0,3 438 43	0,5 972 44	0,2 683 69	0,3 949 28	0,0 063 29	0,0 224 48	0,0 403 67	0,0 250 48	0,0 691 15	0,4 159 89	0,3 906 07	0,1 495 30	0,0 003 83	0,0 465 09	0,0 642 96	0,2 111 89	0,3 872 80	0,9 469 01	0,5 685 19	0,3 906 07	0,0 009 70	0,0 079 25	0,0 083 81	0,0 508 50	0,2 958 12		0,2 880 29	0,3 347 10
47	ArF	VII	0,3 936 62	0,4 353 79	0,0 666 54	0,2 446 14	0,0 525 08	0,1 165 65	0,1 176 16	0,0 014 23	0,0 045 60	0,0 120 76	0,0 496 87	0,0 954 08	0,0 610 37	0,1 174 97	0,4 295 30	0,6 350 82	0,1 157 30	0,3 164 25	0,5 939 59	0,9 230 85	0,8 267 61	0,5 230 39	0,9 546 38	0,7 523 93	0,0 857 33	0,2 108 09	0,3 124 17	0,2 271 66	0,4 409 53	0,8 015 68	0,8 368 73	0,7 003 45	0,0 097 15	0,3 426 36	0,4 214 08	0,8 489 33	0,8 416 32	0,3 189 64	0,6 203 53	0,8 368 73	0,0 204 60	0,1 010 41	0,1 052 31	0,3 629 56	0,9 862 79	0,2 880 29		0,9 212 65
48	ArF	VIII	0,4 560 05	0,5 013 67	0,0 837 62	0,2 906 74	0,0 665 61	0,1 432 89	0,1 445 26	0,0 019 86	0,0 033 27	0,0 090 28	0,0 387 88	0,0 763 10	0,0 479 98	0,0 947 78	0,3 694 69	0,5 601 99	0,0 932 93	0,2 674 94	0,5 215 27	0,9 906 45	0,9 119 24	0,5 955 72	0,9 590 13	0,8 357 86	0,0 696 75	0,1 775 12	0,2 679 26	0,1 919 23	0,3 849 93	0,8 787 78	0,9 147 27	0,6 287 40	0,0 074 12	0,2 951 98	0,3 670 18	0,7 723 80	0,9 195 60	0,3 688 27	0,6 915 79	0,9 147 27	0,0 159 07	0,0 826 05	0,0 861 60	0,3 136 26	0,9 349 30	0,3 347 10	0,9 212 65	
